# Genome and transcriptome sequencing identifies breeding targets in the orphan crop tef (*Eragrostis tef*)

**DOI:** 10.1186/1471-2164-15-581

**Published:** 2014-07-09

**Authors:** Gina Cannarozzi, Sonia Plaza-Wüthrich, Korinna Esfeld, Stéphanie Larti, Yi Song Wilson, Dejene Girma, Edouard de Castro, Solomon Chanyalew, Regula Blösch, Laurent Farinelli, Eric Lyons, Michel Schneider, Laurent Falquet, Cris Kuhlemeier, Kebebew Assefa, Zerihun Tadele

**Affiliations:** Institute of Plant Sciences, University of Bern, Altenbergrain 21, Bern, CH-3013 Switzerland; Swiss Institute of Bioinformatics, Vital-IT, Quartier Sorge - Batiment Genopode, Lausanne, 1015 Switzerland; Ethiopian Institute of Agricultural Research, National Biotechnology Laboratory (Holetta), P.O. Box 2003, Addis Ababa, Ethiopia; Swiss Institute of Bioinformatics, Rue Michel-Servet 1, 1211 Geneva 4, Switzerland; Ethiopian Institute of Agricultural Research, Debre Zeit Agricultural Research Center, P.O. Box 32, Debre Zeit, Ethiopia; Fasteris SA, Ch. du Pont-du-Centenaire 109, P.O. Box 28, Plan-les-Ouates, CH-1228 Switzerland; School of Plant Sciences, Univerisity of Arizona, 1140 E. South Campus Drive, 303 Forbes Building, P.O. Box 210036, Tucson, AZ 85721-0036 USA; Clinic for Parodontology, University of Bern, Freiburgstrasse 7, Bern, CH-3010 Switzerland; Faculty of Science, University of Fribourg, Ch. du Musée 10, Fribourg, CH-1700 Switzerland

**Keywords:** Tef, *Eragrostis tef*, Genome, Transcriptome, Abiotic stress, Prolamin

## Abstract

**Background:**

Tef (*Eragrostis tef*), an indigenous cereal critical to food security in the Horn of Africa, is rich in minerals and protein, resistant to many biotic and abiotic stresses and safe for diabetics as well as sufferers of immune reactions to wheat gluten. We present the genome of tef, the first species in the grass subfamily Chloridoideae and the first allotetraploid assembled *de novo*. We sequenced the tef genome for marker-assisted breeding, to shed light on the molecular mechanisms conferring tef’s desirable nutritional and agronomic properties, and to make its genome publicly available as a community resource.

**Results:**

The draft genome contains 672 Mbp representing 87% of the genome size estimated from flow cytometry. We also sequenced two transcriptomes, one from a normalized RNA library and another from unnormalized RNASeq data. The normalized RNA library revealed around 38000 transcripts that were then annotated by the SwissProt group. The CoGe comparative genomics platform was used to compare the tef genome to other genomes, notably sorghum. Scaffolds comprising approximately half of the genome size were ordered by syntenic alignment to sorghum producing tef pseudo-chromosomes, which were sorted into A and B genomes as well as compared to the genetic map of tef. The draft genome was used to identify novel SSR markers, investigate target genes for abiotic stress resistance studies, and understand the evolution of the prolamin family of proteins that are responsible for the immune response to gluten.

**Conclusions:**

It is highly plausible that breeding targets previously identified in other cereal crops will also be valuable breeding targets in tef. The draft genome and transcriptome will be of great use for identifying these targets for genetic improvement of this orphan crop that is vital for feeding 50 million people in the Horn of Africa.

**Electronic supplementary material:**

The online version of this article (doi:10.1186/1471-2164-15-581) contains supplementary material, which is available to authorized users.

## Background

The increase in the global population, competition of food and biofuel for available land resources, and climate change are all threatening food security. One avenue to alleviate these pressures on our food supply is through better utilization of indigenous or ‘orphan’ crops. These crops have the advantages that they are already well-integrated in the socio-economics of the region, they are the preferred crops for both farmers and consumers, and they provide more stability under rapidly changing environmental conditions and demand. However, they have long been neglected both by commercial breeders and non-profit institutions. Tef [*Eragrostis tef* (Zucc.) Trotter] (family Poaceae, subfamily Chloridoideae) is an orphan cereal that is a staple food for over 70% of the 80 million people in Ethiopia where it grows annually on about 3 million hectares of land [1].

Tef is a prime candidate for genetic improvement both because of its nutritional and health benefits, and because of its tolerance to biotic and abiotic stresses, particularly drought and waterlogging. It can be cultivated in a wide range of ecological niches, including semi-arid areas prone to drought where maize, rice and wheat do not survive. While tef is the most important crop in Ethiopia, it is gaining in popularity as a life-style food in the developed world because it is gluten-free and high in protein, vitamins, and minerals such as calcium, iron and zinc [2–4].

Sensitivity to wheat, barley and rye gluten is related to the presence of specific epitopes in the prolamin gene family [5]. Tef has been proposed as a valuable addition to the diets of celiac patients [6] due to the absence of these epitopes as determined by antibody-based assays [7]. In addition, tef contains a high amount of ‘slowly-digestible’ starch conferring it with a low glycemic index (GI) and is considered a suitable food for Type 2 diabetics [3].

Relatively little sequence data are available for tef. A genetic map was constructed from 151 recombinant inbred lines (*Eragrostis tef* cv. Kaye Murri x *Eragrostis pilosa*) [8]. This data set includes 496 amplifiable simple sequence repeats (SSRs) of which 262 had at least one polymorphism and 192 were placed on 30 linkage groups. In addition, a collection of 3603 EST sequences also from the Kaye Murri cultivar are available [9]. The homologs of two genes involved in plant height (*rht1* and *sd1*) have been cloned and sequenced for 31 cultivars [10].

Tef (2C = 2n = 4x = 40) is an allotetraploid, the result of a genome duplication by hybridization between two diploid progenitors. Tef’s closest relatives within the *Eragrostis* genus are thought to be *E. pilosa* and *E. heteromera* [11], although *E. aethiopica*, *E. barrelieri, E. curvula* and *E. cilianensis* may also be involved in the evolution of tef [12]. As both *E. pilosa* and *E. heteromera* are tetraploid species, the true diploid progenitors of tef remain unknown. The genome size of the Tsedey cultivar (DZ-Cr-37) sequenced here has been estimated as 772 Mbp by flow cytometry [13].

Whole genome duplication events create another copy of all nuclear genes and regulatory sequences at once, providing redundant gene copies for subsequent selection and adaptation. Ancient whole genome duplications are suggested to be associated with adaptive radiations [14] and contemporaneous with extinction events [15], supporting the idea that polyploidy is a driving force of plant evolution. Allotetraploidy, in particular, may contribute to the adaptability to novel and extreme environments [16] and may also increase the fitness of the polyploid in a given environment compared to its diploid progenitors [17]. As the tef genome is relatively small compared to other polyploid crop species, there is considerable interest in tef as both a model plant for polyploid genome evolution as well as for polyploid sequence assembly and analysis.

The Tef Improvement Project at the University of Bern in collaboration with the Ethiopian Institute of Agricultural Research has taken the initiative to support the conventional breeding efforts in Ethiopia using modern molecular techniques. The overall objective of the project is to provide new cultivars improved in traits such as plant architecture, abiotic stress tolerance, and increased yield to subsistence farmers in Ethiopia in a timely manner. The genome and transcriptome sequences reported here reveal genes that have shaped a plant resilient to environmental stresses while also producing nutritious food.

## Results and discussion

### Genome sequencing and assembly

**Figure 1:**
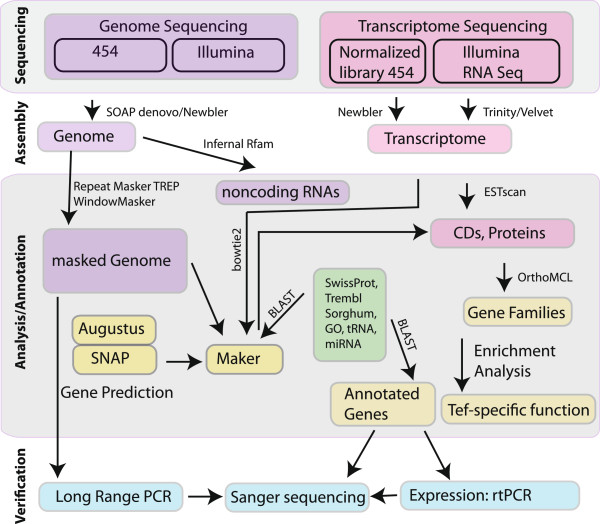
**Overview of the tef sequencing project.** Both the genome and transcriptome of tef were sequenced, annotated, analyzed and verified. The genome was assembled using SOAPdenovo and was then analyzed for transposable elements using WindowMasker, RepeatMasker and TREP. Non-coding RNAs were found with Infernal with the Rfam dataset and genes were predicted using the evidence combiner, Maker. One normalized transcriptome library was produced using 454 pyrosequencing, assembled using Newbler and the genes predicted using ESTscan. Another transcriptome was produced using RNASeq data collected from tef seedlings subjected to various moisture regimes. The sequences were assembled using both Trinity and Oases/Velvet and the coding regions predicted using ESTscan.

The general strategy of the tef genome and transcriptome sequencing, annotation and analysis is shown in Figure 1. The early-maturing improved variety of tef, Tsedey (DZ-Cr-37), was selected for genome sequencing as it can adapt to a wide variety of climates.

Homoeologous genomes in polyploids such as tef have high levels of sequence identity that present enormous challenges to assembly. Currently available genome assemblers are not designed to assemble polyploids and the resulting assemblies are often fragmented [18], chimeric [19] and/or contain false segmental duplications [20].

Often, strategies are applied to reduce the ploidy of the genome in order to simplify the assembly process. These include sequencing the diploid progenitors of a polyploid as was done for tobacco [21] and cotton [22], obtaining a haploid (the drone in ants) [23] or generating a doubled haploid. In plants, generating a double haploid can be done by producing a haploid genome from pollen or seeds and then doubling it to form a homozygous diploid, as done for the potato [24]. Alternatively, BAC libraries can be used to sequence and assemble the entire genome although this is time consuming and expensive.

Our attempts at obtaining a haploid tef for sequencing were unsuccessful. Hence, we sequenced allotetraploid tissue and expected to find a mixture of the A and B genomes. Multiple sequence alignments between the homeologous tef scaffolds and the few Sanger sequences for which we have both an A and a B copy do show some chimerism, for example, the *KO2* gene (Additional file 1: Figure S1). However, the homeolog specificity is intact for large regions of the genes. A two-stage assembly strategy has been successful in separating homeologous sequences from the three genomes of wheat and could be applied to separate the homeologs for regions in which homeolog specificity is required [25].

We generated a total of 40 Gbp from single- and paired-end reads resulting in 44-fold coverage with Illumina HiSeq2000 and seven-fold coverage with 454-FLX pyrosequencing. The libraries had insert sizes of 300 bp, 3 kb, 6.5 kb and 13 kb (Additional file 2: Table S1). The Illumina and the single-end 454-FLX sequences were assembled into contigs using SOAPdenovo [26]. The paired-end and mate-pair Illumina and 454-FLX sequences were then used to link the contigs into scaffolds. GapCloser from SOAPdenovo was then used to close the gaps in the assembly. The assemblies were performed with k-mer values of 25, 29, 33, 37, 41, 45 and 49, and the assembly with k-mer value 41 was chosen based on assembly statistics and the presence of known sequences.

Approximately 80% of the tef genome was represented in scaffolds greater than 1000 bp. Around half of the tef genome was contained in the 3165 scaffolds that were aligned to sorghum following the requirement that at least three syntenic genes be identified. Re-sequencing the tef genome using advanced sequencing technologies with long reads such as Pacific Biosystems RS II technology and Illumina’s Moleculo will contribute to the further improvement of the current assembly.

### Transcriptome sequencing and assembly

A normalized transcriptome library prepared from roots and shoots of tef seedlings generated a total of 350 Mbp of sequence reads using the 454-FLX technology (Additional file 2: Table S2). The reads were assembled with Newbler [27] and resulted in a transcriptome (the ‘454Isotigs’ transcriptome) with 27756 gene clusters and 38333 transcripts (Additional file 2: Table S3).

A second non-normalized library was obtained from various tef tissues subjected to drought and water-logging for the purpose of determining expression. It was then sequenced with the Illumina HighSeq 2000 to obtain 17 Gbp after trimming. The reads were assembled using Trinity [28] and Oases/Velvet [29]. These two assemblies were combined with the 454Isotigs and then clustered with TGICL, resulting in the ‘Extended’ transcriptome containing 28113 gene clusters and 88078 transcripts. Thus the two assemblies revealed substantial differences in the numbers of transcripts but similar numbers of gene clusters.

The large variation in the numbers of transcripts generated from the two assemblies stems from differences in the sequencing data and assembly procedures used. The 454Isotigs transcriptome was created from a normalized library using the Newbler assembler on long reads. On the other hand, the Extended database was constructed from merging three datasets, the 454Isotigs, a Trinity assembly made from short read data and an Oases/Velvet assembly made from short read data, a procedure which created much redundancy. To decrease the redundancy, the resulting transcripts were clustered with TGICL producing the 88,000 transcripts. Interestingly, this approach was also recently used by Nakasugi *et al.* [30] who concluded that it is advantageous to combine the output from many different *de nov*o assemblers. A disadvantage is that this procedure increases the probability of chimeric sequences. The distribution of sequence lengths in the extended dataset has longer sequences than that of the 454Isotigs and is closer to the distribution of sequence lengths in sorghum and to the genomic predictions (Additional file 2: Figure S2). While 99% of the 454Isotigs transcripts can be found in the Extended dataset, only 72% of the Extended can be found in the 454Isotigs (Additional file 2: Table S4). The number of clusters is remarkably similar between the two data sets and sorghum.

### Genome quality assessment

Assembly statistics show that the quality of the tef genome is comparable to that of foxtail millet (*Setaria italica*) [31, 32] and other recently reported genomes [33–36] (Additional file 2: Table S5). The assembled tef genome has a size of 672 Mbp, equivalent to 87% of the estimated size of the sequenced genotype [13]. The distribution of k-mer frequencies was estimated with jellyfish [37] using 100 bp reads from the 300 bp insert-size library. K-mer statistics were used to estimate the genome size and varied from 650 to 700 Mbp depending on the value of k (Additional file 2: Figure S3) [38, 39]. The number of scaffolds greater than 1000 bp in size was 14000 and the scaffold N50 was 85000. The percentages of reads mapped to genomic scaffolds greater than 1000 bp in length are compared in Additional file 2: Table S6. Of the single reads, 84% could be mapped back onto scaffolds. For the 300 bp insert-size library, an average of 74% was mapped with the proper paired-end relationship.

To assess the quality of the genome, four sets of known tef sequences were sought in the genome. First, of the 496 pairs of primers reported for SSR genetic markers [8], 77% were found in the current genome with a distance between them comparable to that expected (Additional file 3: Table S7). Second, as described in Additional file 2: Note 1, two regions, one approximately 10 kbp and the other approximately 8 kbp, were amplified, sequenced with the Sanger method and aligned to the genome. Percentage identities of 99 and 97%, respectively, were obtained for the two constructs (Additional file 2: Table S8; S9). Third, from our previous efforts at genetic improvement, complete or partial sequences of several genes were made by Sanger sequencing. All of the genes and more than 92% of the bases in the genes totaling 31 kbp were found in the draft genome (Additional file 2: Table S10; S11). Fourth, of the 3603 tef ESTs available in the NCBI database, approximately 99% were found. Therefore, the content of the draft genome is sufficient for the intended application to genetic improvement.

### Transcriptome quality assessment

The distribution of lengths of proteins predicted from the Extended and the 454Isotigs transcriptomes were compared to that of sorghum (Additional file 2: Figure S2). While the distribution of protein lengths predicted from the 454Isotigs appears to be skewed toward shorter proteins, the distribution of protein lengths of the Extended dataset is similar to that of sorghum. In addition, the transcriptomes of the 454Isotigs and the Extended were compared to each other and to the sequenced sorghum genome [40]. The percentage of genes and bases aligned are tabulated in Additional file 2: Table S4. For each transcriptome, approximately 99% of the genes had at least a partial hit in the tef genome while 96-99% of the bases in the transcriptomes could be aligned to the genome. Over 90% of the sorghum genes had at least a partial match in the tef genome but many sequences were incomplete or substantially different as the number of bases of sorghum genes aligned to tef was less than 60%. The 454Isotigs appeared to be a subset of the Extended transcriptome as almost all of the 454Isotigs were found in the Extended set while only 23% of the latter were present in the 454Isotigs.

### Comparison to other grasses

**Figure 2:**
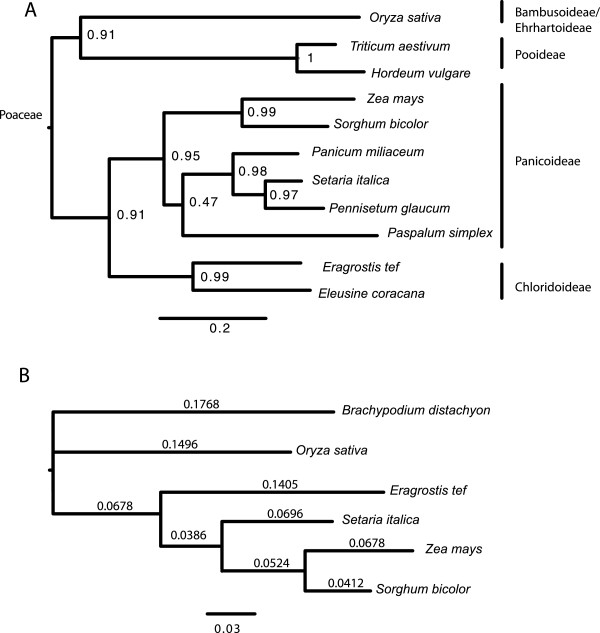
**Phylogenetic tree for selected cereals from the grass (Poaceae) family including tef (** ***Eragrostis tef*** **). A)** Partial sequences of the WAXY gene from barley (*Hordeum vulgare*, X07931), bread wheat (*Triticum aestivum*, KF861808), finger millet (*Eleusine coracana*, AY508652), foxtail millet (*Setaria italica*, AB089143), maize (Zea m*ays*, EU041692), *Paspalum simplex* (AF318770), pearl millet (*Pennisetum glaucum*, AF488414), proso millet (*Panicum miliaceum*, GU199268), rice (*Oryza sativa*, FJ235770.1), sorghum (*Sorghum bicolor*, EF089839), and tef (*Eragrostis tef*, AY136939) were obtained from the NCBI database. The maximum likelihood tree was inferred using PhyML and the default model of HKY85 + G. The scale bar reflects evolutionary distance, measured in units of substitution per nucleotide site. Branch support was inferred using the Shimodaira–Hasegawa-like (SH) aLRT provided by PhyML. **B)** Phylogenetic Tree of the Complete Grass Genomes. Protein supergenes with an aligned length of 260398 amino acids and constructed from orthologous sequences were used to infer a maximum-likelihood tree using PhyML with the WAG substitution matrix and a gamma model with four classes and an alpha parameter value estimated to be 0.489. Branch lengths reflect the estimated number of amino acid substitutions per site. ML bootstrap values were all 100%.

Tef belongs to the grass family Poaceae which includes all cereal crops, and to the subfamily Chloridoideae and is the first sequenced member of this subfamily. The evolutionary relationships between the grasses are shown by the phylogenetic tree constructed from the waxy gene (Figure 2A). The closest cultivated species to tef is finger millet (*Eleusine coracana*), the genome of which is not yet sequenced. The closest subfamily to the Chloridoideae is the subfamily Panicoideae that includes sorghum (*Sorghum bicolor*) and maize (*Zea mays*). A phylogenetic tree constructed from orthologous genes between the five grasses which have been heretofore sequenced shows the evolutionary distances between the grasses (Figure 2B). Of the sequenced genomes, the closest to tef are foxtail millet (*Setaria italica*) and *Sorghum bicolor*.

**Figure 3:**
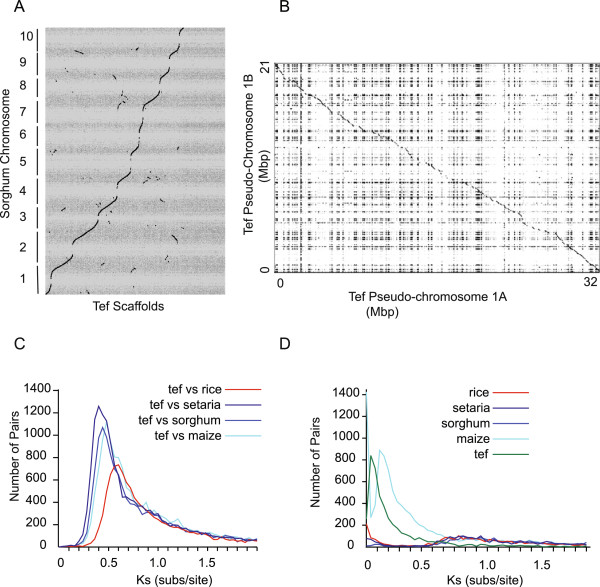
**Comparison of tef to other grasses. A)** Syntenic dotplot between tef scaffolds (x-axis) and sorghum chromosomes (y-axis) produced by CoGe. Scaffolds of tef have been ordered and oriented based on synteny to sorghum (minimum of 3 syntenic genes, see: http://goo.gl/ECKmA9) and joined to create a pseudo-assembly. Each dots represents a syntenic gene pair between tef and sorghum. For each sorghum position, two tef scaffolds, one from the A genome and one from the B genome, are expected. The sinusoidal shape is a result of very few tef scaffolds aligning to the gene-poor centromeric regions of sorghum. **B)** Tef pseudo-chromosomes were then sorted into tef A and tef B pseudo-chromosomes. A dot plot of the 1A and 1B pseudo-chromosomes shows the correspondence between the A and B pseudo-chromosomes. **C)** The distributions of pairwise synonymous substitutions per synonymous site estimated between tef and other genomes. The corresponding dates can be found in Additional file 5: Table S13. **D)** Histogram of synonymous rate values (Ks) for all syntenic gene-pairs within the tef and within the maize genomes. The dates estimated from modes of the peaks using a molecular clock rate of 6.5 × 10^-9^ substitutions per synonymous site per year can be found in Additional file 5: Table S13.

The tef genome and the genes predicted in the genome from the Maker genome annotation pipeline combiner [41, 42] were uploaded to CoGe [43], a platform containing a wealth of genomes and providing interfaces to numerous tools for genome alignment, comparison, and visualization. CoGe’s tool SynMap [44] identifies orthologous genes between two genomes using sequence homology and synteny. SynMap was used to identify syntenic tef scaffolds with at least five (alternatively three) genes having a collinear genomic relationship to sorghum genes [45]. Alignment of these scaffolds to the 10 sorghum chromosomes produced 10 artificial tef ‘pseudo-chromosomes’. A total of 2468 scaffolds containing approximately 346 Mbp were ordered by this alignment of tef scaffolds to sorghum chromosomes (Figure 3A; Additional file 2: Table S5; Additional file 2: Note 2). These pseudo-chromosomes were constructed from a mixture of the A and B genomes with the two homeologous scaffolds close to each other in the tef pseudo-chromosome. The scaffolds aligning to each sorghum chromosome were further sorted into A and B pseudo-chromosomes, by sequentially placing them into two groups based on overlap. The first scaffold was placed in A, if the next scaffold had more than a 75% overlap with one in A, it was put in B. This was then repeated for all scaffolds in order, resulting in random placement of each scaffold into the A and B pseudo-chromosomes. Dotplots [46] of the A and B pseudo-chromosome show that they are homologous over large areas and also show the potential of syntenic mapping in sorting polyploid genome assemblies (Figure 3B; Additional file 4: Figure S4). A more sophisticated algorithm to identify which scaffolds belong to the A and B genomes is under investigation.

Genetic maps show the relative positions of loci with a distance based on the amount of recombination between them and are developed through markers which sort together based on phenotype. Physical maps give the distance between any two loci in units of base pairs. Establishing the relationship between the genetic and physical maps reveals the location of and distance between genes that are recombining together.

**Figure 4:**
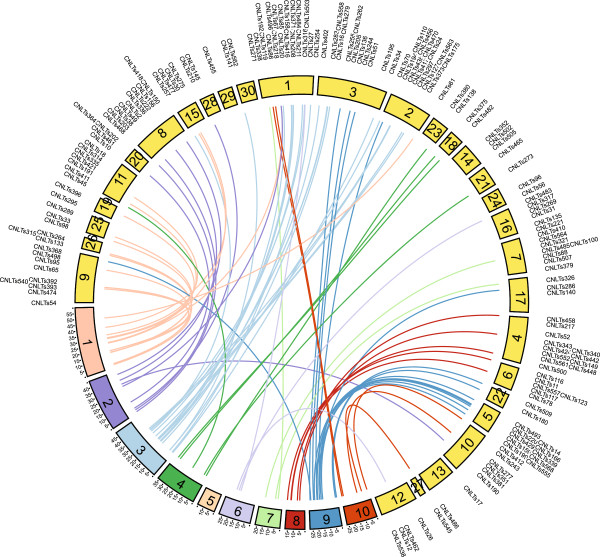
**Relationship between tef genetic map and tef pseudo-chromosomes.** The 30 linkage groups from the genetic map of Zeid *et al.* are depicted in yellow with labels corresponding to the location of their CNLT SSR markers. The ten tef pseudo-chromosomes are colored in various colors with colored lines connecting the physical location of each CNLT marker on the tef pseudo-chromosomes to its location on the genetic map. Lines depicting mapping of genetic markers to tef pseudo-chromosomes are shown with the color of the pseudo-chromosome with the most overlap. The linkage groups of the genetic map have been ordered to minimize overlap of the connections and thus indicate which of the 30 linkage groups are homeologous. A translocation between sorghum and tef can be seen between linkage group 3 and tef pseudo-chromosomes 3 and 9. The units of the tef pseudo-chromosomes are Mbp.

The available genetic map consists of 30 linkage groups instead of the 20 expected based on the chromosome number because there are not enough markers to join all of the linkage groups into chromosomes. By connecting the physical and genetic maps, we can establish which linkage groups correspond to each chromosome and which linkage groups are homeologous. The tef genome is not assembled into the 20 chromosomes so the pseudo-chromosomes created by mapping to sorghum were aligned to the genetic map of tef and used to suggest a putative order of the linkage groups [47] (Figure 4; Additional file 2: Table S12). For example, it can be seen that linkage groups 23, 18 and 21 all map to tef pseudo-chromosome number 4 (dark green lines) while linkage groups 1, 2 and 3 all map to pseudo-chromosome number 3. These could either be homeologous linkage groups or linkage groups that did not have enough markers recombining together to be joined.

Inversions and translocations between the tef and sorghum genomes could be seen such as the chromosomal translocation between tef linkage group 3 and tef pseudo-chromosomes 3 and 9 as well as an inversion between linkage group 3 and 5 that correspond to pseudo-chromosome 9. The physical distance and genetic distance were compared for 33 marker pairs and ranged between 0.003 and 15 cM/Mbp (Additional file 2: Table S12). The variation in the estimates might be related to the sparsely covered genetic map or because of differences in tef recombination rates.

Identification of the set of orthologous genes between two genomes enabled genome-wide comparisons. Codeml of PAML [48] is integrated into CoGe. Codeml is a program for the reconstruction of ancestral sequences using a collection of codon models which in turn allows estimation of synonymous (Ks) and non-synonymous (Ka) substitution rates over trees, branches or sites. The ratio of Ka to Ks is useful for the detection of selection in protein-coding DNA sequences while Ks alone can be used as a molecular clock. CoGe first identifies orthologous genes based on collinearity and then returns Ka and Ks values for the complete set of orthologous genes between two genomes. The Ks distribution between two genomes can be used to estimate the divergence date between the two species; the mode(s) of the distribution represents either a speciation or duplication event. The divergence date corresponding to the mode can then be estimated using a substitution rate, for plants a substitution rate of 6.5 × 10^-9^ synonymous substitutions per synonymous site per year has been proposed [49].

The SynMap function of CoGe was used to do pairwise comparisons of the following genomes: *Eragrostis tef* (Coge id 38364), *Sorghum bicolor* (Coge id 38364), *Zea mays* (Coge id 333, B73 refgen_v2 assembly, working gene set annotations 5a), *Oryza sativa japonica* (Coge id 3) and *Setaria italica* (Coge id 32546, with CNS PL2.0 l v2.1, id2240) (Figure 3C; Additional file 5: Table S13). From the mode of the distribution at 0.47 substitutions per site, the divergence date between sorghum and tef was estimated to be around 36 million years ago (MYA). All genome-wise estimates of Ks between tef and other Poaceae are in Additional file 5: Table S13 and are comparable to those found by Smith *et al.* [10].

### Allotetraploidy

Tef is an allotetraploid, the result of genome duplication by hybridization between two diploid progenitors. The whole genome duplication of tef is relatively recent in the history of the grasses and therefore it provides a unique snapshot into the consequences of such an event at this time point.

Whole genome duplications in grasses can be revealed by the distribution of Ks values within a genome. If a genome has undergone a recent whole genome duplication, the distribution of Ks values computed between the genome and itself will show a peak whose Ks value is an estimate of the evolutionary distance between the homeologs. In order to determine the evolutionary distance between the two sub-genomes in the grasses, the distribution of Ks values between homeologous genes within each genome was computed using CoGe. Maize and tef had evident peaks corresponding to recent whole genome duplications while the other grasses did not (Figure 3D). The mode of the tef Ks distribution was 0.05 substitutions per codon, while that of the maize distribution was 0.15, corresponding to genome duplications for tef and maize occurring approximately 4 and 12 MYA, respectively, using a substitution rate of 6.5 × 10^-9^ synonymous substitutions per synonymous site per year [49] (Additional file 5: Table S13). These divergence dates are in agreement with previous estimates for tef [10] and maize [50]. A small broad peak centered around 0.9 substitutions per site is present in all genomes and corresponds to the rho whole genome duplication, a duplication that occurred prior to the divergence of the grasses [51].

Alignment of the tef A and B pseudo-chromosomes showed that the average sequence identity between the aligned nucleotides of the A and B pseudo-chromosomes over the entire genome was 93% not counting the frequent indels (Additional file 2: Table S14). Eight genomic sequences for which Sanger sequences were available for both homeologous copies had an average of 96% sequence identity in the coding region and 82% in the non-coding region (Additional file 2: Table S15).

In addition, as there are two homoeologs expected for each gene in the genome, two copies of each transcript of the 454Isotigs and the Extended transcriptomes were sought in the genome. Statistics were tabulated for longest aligning copy, ‘copy 1’ and the second-longest aligning copy, ‘copy 2’ (Additional file 2: Table S16). For the 454Isotigs, 96% of the transcripts were found and 86% of the transcripts had a second copy, indicating that the majority of the genes are present with two copies.

### Discovery of novel SSR markers

Simple Sequence Repeats (SSRs) or microsatellites are sequences of 2–6 nucleotides that repeat from 3 to 100 times. They are highly polymorphic and therefore often used as molecular markers for breeding indigenous crops such as tef. Although high-throughput sequencing of SNPs for genetic markers is becoming more commonplace, SSR markers are still important for breeding in the developing world. Marker-assisted breeding depends on the natural variation in the population. Of the 496 pairs of primers reported for SSR markers in tef [8], only 77% were found in the current genome mainly due to its source. The SSR markers were developed from ESTs which represent only the transcribed regions while the genome sequence contains both transcribed and non-transcribed regions. Hence, the primer sequence of an SSR marker might be split into two places in the genome due to the presence of an intron. These divided markers would not be detected in the genome. Markers could also be missing from the genome either because they were not sequenced or because of natural variation between the different tef cultivars used for sequencing.

About 49600 SSR repeats (di-nucleotide or larger) or about one every 13565 bases were found in the tef genome (Additional file 2: Table S17) using MISA [52]. MISA is a tool developed to systematically search for SSRmotifs. Perl scripts provided on the MISA website to interface MISA with Primer3 [53, 54] were used to design primers for all SSRs with repeat size of three or more (Additional file 6: Table S18 for the entire genome). Several SSRs having a close proximity to a gene cluster controlling yield or drought on the genetic map [47] but not in coding regions were chosen for PCR amplification. To maximize the chance of polymorphisms, the SSRs were chosen to be in noncoding regions.

A novel SSR located near known markers on linkage group 9 was found to have differences between the Alba and Tsedey cultivars of tef, and was further investigated (Additional file 2: Figure S5A; Additional file 2: Table S19). Approximately 200 bp surrounding the SSR was sequenced for 20 tef cultivars (of which Tsedey, Dukem, Magna and Quncho are improved varieties) as well as four wild *Eragrostis* species (Additional file 2: Figure S5B). The genotypes were variable at 32 positions including indels. Addisie, Beten, Gommadie, Kaye Murri, Magna, Rosea, Tsedey and Variegata shared the same number of repeats of the CTCCT motif while Ada, Alba, Balami, Dabbi, Dukem, Enatite, Gea Lammie, Karadebi, Manyi, Quncho, Red Dabbi and Tullu Nasy, had one less (Additional file 2: Figure S6). In addition, Tullu Nasy, Alba and Balami share an indel. Of the wild species, *E. pilosa* and *E. minor* had two fewer instances of the repeat while *E. curvula* and *E. trichodes* had three fewer occurrences. The sequence alignment and the most parsimonious phylogenetic tree based on all variable positions support these relationships and place the Gommadie and Kaye Murri ecotypes, which both have a semi-compact panicle, to the same group (Additional file 2: Figure S7). A parsimony tree with the same score could be obtained by putting *E. minor* in the same clade as *E. pilosa* A to the exclusion of *E. pilosa* B.

Discovery of a novel SSR marker indicates that the genome has applications in marker-assisted breeding. This new marker was located on scaffold2788 at position 199935 only 90 kbp from the *rht1* (reduced height) gene responsible for semi-dwarf stature in wheat and rice [55]. It separates 20 tef genotypes with diverse agronomic and morphological traits [56] and four closely related wild *Eragrostis* species into two groups, while other polymorphisms in the surrounding sequence further divide the groups. The phylogenetic relationship between several natural accessions as well as two improved varieties could be determined from sequencing the locus around the SSR. Among the four improved varieties, two (Magna or DZ-01-196 and Dukem) were developed via widely practiced selection procedures while the other two (Quncho and Tsedey) were developed through introgressions between improved tef cultivars. A cross between the high-yielding Dukem variety and the white-seeded Magna variety produced Quncho, the most popular variety in Ethiopia [57]. For the region sequenced here, the Quncho sequence is identical to that of the parent Dukem and not the parent Magna. Although most of the polymorphisms occur between the different *Eragrostis* species, the tef natural accessions Kaye Murri and Ada also have point mutations.

### Genome annotation and analysis

Genes were predicted in the genome using the Maker evidence combiner [41, 42]. Over 92% of the predicted genes were supported by the Extended transcriptome. The distribution of gene lengths of the predicted genes was consistent with that of the sorghum genome (Additional file 2: Figure S2). Annotation was performed with the Swiss Institute of Bioinformatic’s Praise Annotation System, designed to use manually curated rules to combine annotation from different sources (Additional file 2: Note 3). The numbers of entries with various annotations for the different datasets can be found in Additional file 2: Table S20.

Using WindowMasker with the default settings, 14% of the genome was masked (Table 1) which is substantially lower than that expected from other grasses such as sorghum with ~ 61% [40]. RepeatMasker using the TREP database [58] as input found only 6% of the draft genome to be transposable elements, 3.9% retroelements (class I transposable elements) and 2% DNA transposons (class II transposable elements). The number of repeat elements found was small but given the genome assembly procedure used, it is expected that the number of repeats is greatly reduced due to collapsed sequence assemblies [59]. Infernal [60] with the Rfam database [61, 62] identified 80 rRNAs (99 in foxtail millet), 1184 tRNAs (577 in sorghum, 704 in foxtail millet, 1163 in maize), 570 miRNAs (159 in foxtail millet) and 834 snRNAs (382 in foxtail millet) [32, 63].

**Table 1:** **Summary of genome annotation**

Type		Number or copies	Total size (bp)	Percent of genome
**A. Repetitive Elements**				
Masked with WindowMasker			95556652	14.2
Masked with RepeatMasker			39352657	6.6
MITEs		77908	10783061	1.6
**B. Noncoding RNAs**				
rRNA		80	13580	
tRNA		1184	88295	
miRNA		570	73999	
snRNA		834	95690	
**C. Proteins**				
Dataset	Number of clusters (unigene)	Number of transcripts	Average length of CDS (bp)	Percentage of Genome
454Isotig	27756	38333	285	1.8
Extended	28113	88078	331	4.7
Maker prediction		42052	395	3.3

Number and size of A) repetitive elements, B) noncoding RNAs, and C) proteins. Repetitive elements were quantified using WindowMasker, RepeatMasker and MITE-Hunter. The non-coding RNAs were found using the Infernal software with the Rfam database. The transcripts in two assemblies (454Isotigs, Extended) and the predicted genes in the genome (Maker) are also compared.

Miniature inverted-repeat transposable elements (MITEs) are a special type of class II non-autonomous transposable elements (TE) that are abundant in the non-coding regions of the genes of many plant and animal species [64]. The number of MITEs found in the tef genome is 77908 which is 1.6% of the genome size (Table 1). Around 56000 MITEs were identified in sorghum, comprising 1.7% of the genome [40] while Oki *et al.* [65] reported 73500 MITEs in rice, comprising 5.2% of the genome.

### Abiotic stress related genes in tef

Although tef is relatively drought tolerant, moisture scarcity is among the major yield limiting factors in tef production [66] and severe drought remains a critical problem for Ethiopia [67]. One of the goals of the Tef Improvement Project is to identify genes involved in tef’s abiotic stress tolerance, in particular those for drought-tolerance, and to use them to develop more drought-tolerant cultivars. Tolerances to abiotic stresses such as tolerance to drought, salt, and water-logging are complex traits controlled by many genes. Access to the genomic sequence of tef enables the transfer of knowledge of these genes gained from well-studied crops like maize and rice to the application of molecular-supported breeding efforts in tef. In addition, the tef genome provides a new pool of abiotic stress target genes.

Twenty-six *Arabidopsis*, rice and sorghum genes known to be involved in abiotic stress response were investigated by aligning each query sequence to the tef genomic sequences (Additional file 2: Table S21). Two homeologs were sought in tef. For comparison, they were also aligned to the sorghum and foxtail millet genomes. All of the genes were found at least partially in all genomes. The percentage of query sequence nucleotides aligned to the tef genome differed for each gene and ranged from 57 to 100%. These percentages were similar to those found in foxtail millet. The percentages of the query sequences aligned to sorghum were higher than both tef and foxtail millet, not surprising given that some of the query sequences were from sorghum and that the quality of the sorghum genome is higher. For 21 of the 26 genes investigated, two homeologs were found in tef.

One of the goals of this project is to identify molecular breeding targets. To investigate which genes might have special adaptations in tef, gene families that have undergone expansion or contraction were sought as this might provide some clues to the adaptation of tef to extreme climatic conditions in general and specifically to drought tolerance. For this reason, the number of family members for each of these genes was counted in tef and the other grasses and tabulated (Additional file 2: Note 4; Table S22).

Among these genes known to be involved in drought tolerance in several plant species, we have singled out the SAL1 gene which is involved in abiotic stress tolerance in both monocts and dicots. Mutants with down-regulated or inactivated SAL1 genes have been found to have an increased drought tolerance in *Arabidopsis* [68] and wheat [69]. From Additional file 2: Table S22, SAL1 was also identified as a family in which gene duplication may play a role in the drought response of tef. For these reasons, SAL1 was chosen for detailed analysis.

**Figure 5:**
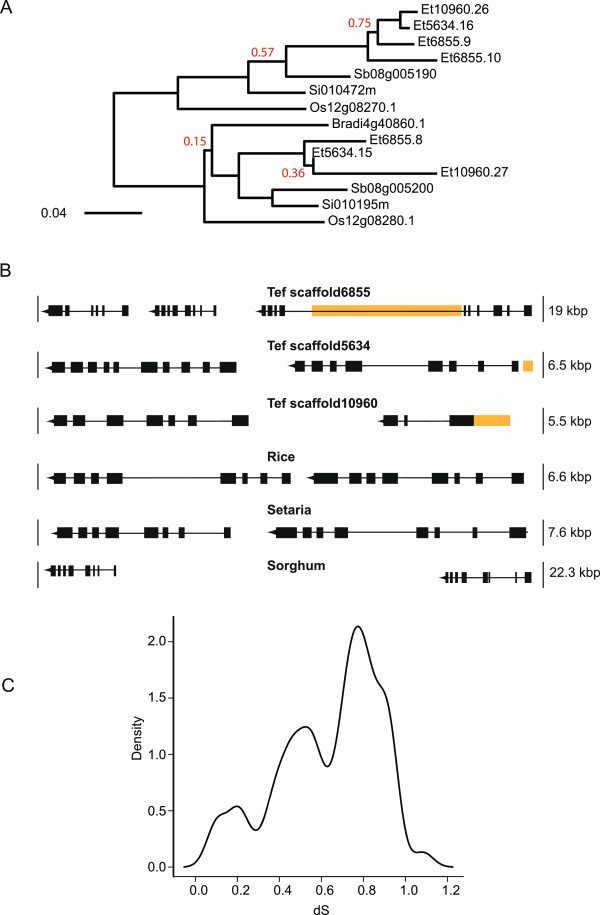
**SAL1 gene in tef and other grasses. A)** The SAL1 gene duplicated before the divergence of the grasses and has also undergone several recent duplications in one branch of the phylogenetic tree. The ML tree was constructed using the WAG protein substitution model implemented in PhyML (version 3.0). Branch support was inferred using the conservative and non-parametric Shimodaira–Hasegawa-like (SH) aLRT provided by PhyML. Only branch support values less than 0.85 are shown. Abbreviations: Et: *Eragrostis tef;* Sb: *Sorghum bicolor;* Os: *Oryza sativa;* Bradi: *Brachypodium distachyon;* Si: *Setaria italica.* **B)** Comparison of orthologous syntenic genomic regions between tef, sorghum, and setaria. The SAL1 gene appears on three scaffolds in the tef genome, twice as tandem duplicates and once as a tandem triplicate and is found as a tandem duplicate in rice, setaria and sorghum. Orange blocks indicate unsequenced regions created from the scaffolding. **C)** Distribution of Ks values for all pairwise comparisons of SAL1 gene family members.

Family members of the SAL1 gene were identified by using homology searching of the sorghum SAL1 gene in the genomes of five grasses. Seven copies or partial copies of the SAL1 gene were found in tef compared to 1–2 in the other grasses investigated (sorghum, foxtail millet, *Brachypodium* and rice). The phylogenetic relationship of the members of the SAL1 family is shown in Figure 5A. Two gene subfamilies are present, probably the result of a tandem duplication which took place before the divergence of the grasses. Both copies have been retained by all grasses with the exception of *Brachypodium* for which only one copy was found. For tef, in addition to a second homologous copy, it appears that the entire region has been duplicated yet another time as this sequence of genes appears in three scaffolds in its entirety (scaffold6855, scaffold5634, scaffold10960). In addition, on scaffold6855, SAL1 appears as a tandem triplicate (Figure 5B) indicating further gene duplication in this family. The presence of this particular tandem triplication was confirmed by designing several sets of PCR primers in the region spanning 12.5 kb and sequencing the PCR products using the Sanger method. As shown in Additional file 2: Figure S8, all three SAL1 tandem genes identified in the scaffold are present in the tef genome.Analysis of dN/dS values identified no branches of the tree evolving under positive selection. The distribution of dS values between the members of the SAL1 family examined shows a peak at 0.8 substitutions per codon which reflects the distance between the two subfamilies that have been diverging since the original duplication (Figure 5C). A peak around 0.5 substitutions per codon reflects the speciation between the most distant members of each subfamily. The smallest dS value of 0.1 was located between Et10960.26 and Et5634 indicating either a recent duplication or gene conversion. The dS value between Et6855.9 and Et6855.10 indicates that this is the most recent duplication of the triplication on scaffold6855.

In addition to the SAL1 gene, several other genes known to be involved in abiotic stress tolerance have been identified as potential targets for improvement by observing changes in the number of family members both in the genome and transcriptome constructed separately from the control, drought and waterlogged RNASeq samples. These included B-glucanase [70], SGR [71] and ERD1 [72], all involved in the response to drought (Additional file 2: Table S22). These potential candidates will be the next targets investigated in the laboratory.

### Prolamin family genes related to gluten sensitivity

Celiac disease in humans is caused by an immune response to specific amino acid sequences, called epitopes, that are present in wheat, barley and rye gluten. The family of genes responsible is the prolamin gene family [5]. Antibody-based assays have shown that tef does not contain the offending epitopes [7]. Possession of the genomic sequence allows for confirmation of these assays. A comprehensive search of all wheat, barley and rye epitopes causing immune reactions in celiac patients resulted in 96 epitopes that stimulate T-cells [5]. These 11-, 12-, 13-, 16- and 20- amino acid-long epitopes were sought in tef, *Brachypodium*, barley, rice, maize, *Setaria* and sorghum genomes as well as the wheat and rye sequences at NCBI. None of these epitopes were found outside of the wheat, barley or rye genome (Additional file 2: Note 5; Table S23) confirming that rice, maize, sorghum and tef products may be safely consumed by celiac patients.

**Figure 6:**
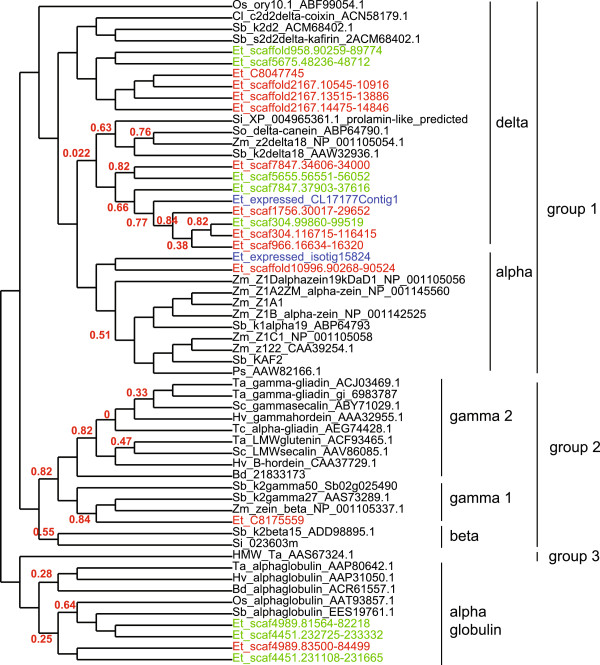
**Phylogenetic tree of prolamins in grasses.** The protein sequences included in the analysis follow those of Xu and Messing [74] with the addition of tef sequences from this work. The repeat regions were edited out of all sequences which were then aligned using MAFFT. The ML tree was constructed using the WAG protein substitution model implemented in PhyML (version 3.0). The gamma shape parameter was fit to 2.845 and the proportion of invariant sites was estimated to be 0. Branch support was inferred using the conservative and non-parametric Shimodaira–Hasegawa-like (SH) aLRT provided by PhyML. Only branch support values less than 0.85 are shown. The tree is represented here by a cladogram so there are no meaningful branch lengths. Expressed tef sequences are in blue, tef sequences with a lesion (frame shift or stop codon in the coding region) are green and the remaining tef sequences are red. The grasses included are: Bd: *Brachypodium distachyon*; Cl: *Coix lacryma*; Et: *Eragrostis tef*; Hv: *Hordeum vulgare*; Os: *Oryza sativa*; Ps: *Panicum sumatrense*; Sb: *Sorghum bicolor*; Sc: *Secale cereale*; Si: *Setaria italica*; So: *Saccharum officinarum*; Ta: *Triticum aestivum*; Tc: *Triticum compactum*; Zm: *Zea mays*.

Prolamin genes were sought in the tef genome by using BLAST [73] to search for homologs to grass prolamin protein sequences described by Xu and Messing [74]. In the tef genome, 23 sequences were found (Figure 6; Additional file 2: Table S24), often as tandem duplicates, with the majority being located on tef pseudo-chromosomes 3, 7, 9 and 10. Many of the genes were found to have stop codons or frame shifts. Two were expressed either in the transcriptome or in mass spectrometry analysis of tef [75].

As detailed in Xu and Messing [74] grass prolamins fall into three groups. Group one includes alpha- and delta- prolamins. Alpha prolamins are present in the Panicoideae (millet, maize, sorghum) but not rice or Pooideae (wheat, barley, *Brachypodium*). They are the youngest family of prolamins and thought to have originated from delta prolamins. One copy of tef alpha prolamin was found in both the genome (scaffold10996) and the transcriptome (isotig15824), indicating that it is being expressed (Figure 6; Additional file 2: Table S24). The majority of the genomic copies found in the tef genome are delta prolamins including one expressed in our transcriptome studies (CL17177Contig1). The delta group includes a tandem triplication on scaffold2167 as well as a tandem duplication on scaffold7847.

Group two of the prolamins, the largest group, contains gamma and beta zeins/kefarins in maize and sorghum as well as the S-rich prolamins (alpha- and gamma-gliadins, gamma- and beta-hordein, gamma secalin, and the LMW prolamins in wheat, barley and rye). One genomic copy of a beta-like tef sequence and one genomic copy of a gamma-like tef sequence were found. For this group, the Pooideae cluster together as do sorghum, maize and tef.

Group three, comprised of prolamins only found in Pooideae, is more closely related to alpha-globulins (non-prolamins) than to other prolamins. Tandem duplicates of two alpha-globulin genes were found on scaffold4989 and scaffold4451, both located on tef pseudo-chromosome 9.

The only tef prolamin sequences found in the literature were produced by Tatham *et al.* [75] who identified and isolated tef prolamin sequences using SDS-PAGE and HPLC. Six peaks were resolved with HPLC, for two of them (tef2 and tef6), the first 30 amino acids were sequenced. They concluded that both tef6 and tef2 were alpha prolamins based on the high similarity of their amino acid compositions to the amino acid composition of the alpha-prolamins of maize. The sequence of tef6, similar to scaffold10966, is found in the transcriptome as isotig15824 and appears to be an alpha prolamin. The 30 amino acids of Tatham’s peptide tef2 were found several times in the tef genome; the locations are tabulated in Additional file 2: Table S24. However, no prolamin genes were found in these regions when searching for homology with members of the prolamin family. Therefore the full sequence and status of tef2 remain unknown. The 30 amino acids of tef2 were also not found in the transcriptome.

Both the tef and the finger millet sequences had N-terminal deletions compared to zeins as is the case with many of the tef sequences found in this analysis. Surprising is the large amount of duplication that has occurred in the delta prolamins of tef. Seed tissue was not included in the sequencing library; therefore, it is uncertain if all of the expressed genes have been detected.

## Conclusions

The genome sequence of tef, an indigenous and economically important cereal crop in the Horn of Africa, is a valuable resource for comparative and functional genomic studies of grasses, particularly for abiotic stress tolerance and healthy nutrition for which tef can be considered as model plant. In general, the genome of tef provides a molecular basis for improvement techniques that will provide new cultivars for subsistence farmers in Ethiopia. Furthermore, the genome sequence of tef is a starting point for the exploration of the genetic diversity in tef natural accessions and mutagenized populations. It paves the way for the application of modern techniques such as EcoTILLING, Genome Wide Association Studies (GWAS), Genotyping by Sequencing (GBS) and RNASeq in harnessing the rich natural variation present in the tef germplasm.

## Methods

### DNA sample preparation

Genomic DNA was extracted from two-week-old Tsedey (DZ-Cr-37) seedlings using the Nucleospin Plant II maxi kit (Macherey-Nagel) according to the supplier’s protocol. The DNA quantity and quality were measured using an ND-1000 Spectrophotometer (Nano-Drop, USA) in which the ratio of 260/280 wavelengths was 1.87.

### Genome sequencing

Illumina sequencing reads were collected from a HiSeq2000 Machine from Fasteris, Geneva [76]. 454 reads were sequenced using the 454-FLX technology from the Functional Genomic Center, Zürich, Switzerland [27, 77] and Macrogen, Korea [78] according to standard protocols. For HiSeq2000 sequencing, various library preparations and sequencing protocols were investigated and the best results obtained using the Accuprime polymerase (Invitrogen, Carlsbad, CA) and following the protocol for high GC content.

### Genome assembly

FastQC [79] was used for quality control. Sequence pairs containing adaptor or primer sequences were removed and the sequences were trimmed such that all positions had a Phred score greater than 28. All Illumina and the single-end 454-FLX sequences were assembled into contigs using SOAPdenovo [80] with parameters ‘-L 100, -R yes, max_rd_len = 85’. The paired-end and mate-pair Illumina and 454-FLX sequences with insert sizes of 3 kb, 4 kb, 6 kb and 13 kb were then used to link the contigs into scaffolds. GapCloser was used with parameter ‘-p 31’.

### Repeat analysis

WindowMasker [81] with its default settings and the ‘-dust’ option was used to identify repetitive sequences based on n-mer frequency counts in the genome. The ‘-dust’ option identifies and masks regions of low complexity regions in addition to interspersed repeats. In addition, repeats were masked using RepeatMasker [82] with the TREP nucleotide and protein databases of plant repetitive elements [58]. MITE-Hunter [64] with the default parameters was used to find MITEs.

### Gene prediction in the genomic sequence

Gene predictions were performed using the evidence combiner, Maker, on the unmasked genome using Augustus predictions [83] with the maize matrix. The Trinity transcriptome before clustering was used as EST evidence and the complete Uniprot Swissprot database from September 2012 was used as protein homology evidence. For repetitive sequence finding, all model organisms were used from RepBase, the TREP11_beta database was used as the organism specific repeat library and the te_proteins.fasta file provided with Maker was also used. After the first iteration, SNAP [84] was trained on the Maker output from the first run and then Maker was rerun. Two such iterations were performed.

For identification of tRNA genes, tRNAscanSE version 1.3.1 was used [85]. All non-coding RNAs [60] (including tRNAs) were identified using the perl control script rfam_scan.pl version 1.0.4 with Infernal version 1.0 and the Rfam database version 11.0. In addition tRNAscan version 1.3.1 was used. A database of tef MITEs was created using MITEHunter version 11–2011 with the default parameters [64]. This database was then used in ‘RepeatMasker version 3.3.0 with options’ –nolow –no_is to find the complete set of MITEs in the tef genome.

### RNA sample preparation and sequencing for the normalized library

All RNA was extracted from the improved tef variety Tsedey (DZ-Cr-37). To construct the normalized library, leaves from two-month-old plants, roots from ten-day-old seedlings grown in vitro, and seedlings exposed for three weeks to three conditions, namely waterlogging, drought, and normal watering, were harvested. The RNA was extracted from individual samples using the RNeasy kit (Qiagen, Switzerland) according to the supplier’s protocol. The quality and quantity of RNA was quantified using ND-1000 Spectrophotometer whereby the average 260/280 ratio was 2.0 indicating good quality RNA. Five μg of RNA from each of the above five samples was pooled. Library construction and sequencing was provided by MWG (Germany). A total of one million single-end reads (equivalent to 351 Mbp) with a mode of 400 bp were collected. The data was filtered for primer and adaptor sequences and trimmed such that all positions had a Phred score greater than 28.

### Transcriptome library construction and sequencing for RNASeq

The RNA extracted from plants grown under waterlogging, drought, and normal watering conditions as described above, were sent to Fasteris (Geneva, Switzerland) for further quality testing and sequencing using Illumina HiSeq2000 with the intention of analysis for differential expression. From each of the three samples, two different libraries were prepared. Six cDNA libraries were sequenced to generate a total of 205 million single-end reads as shown in Additional file 2: Table S2. Before assembly, the reads were trimmed such that the Phred quality scores were above 28. In addition, all primer and adaptor sequences detected by FastQC were removed.

### Transcriptome sequencing and assembly

Two transcriptomes (one from the normalized library ‘454Isotigs’ and an ‘Extended’ set) were assembled. The 454-FLX sequences together with 3608 tef EST sequences (equivalent to about 1.8 Mbp) downloaded from NCBI were assembled with Newbler 2.3 [27], resulting in an assembly referred to as ‘454Isotigs’ and having 27756 Isogroups and 38437 Isotigs. Prediction of coding regions using ESTScan ‘with m = 100, N = 0, w = 60, and using the *Zea mays* scoring matrix’ resulted in 33098 predicted genes.

The RNASeq data from Illumina was assembled into two datasets, namely Illumina-Trinity and Illumina-Oases/Velvet.

#### Illumina-trinity

The nine Illumina RNASeq data sets were assembled with Trinity [28] 2012-01-25p1 using the trinity.pl wrapper and options ‘--bflyHeapSpace 15G’ and ‘--no_meryl’. In addition, the GNY1/GNY10 (control), GNY2/GNY11 (drought), and the GNY3/GNY12 (water-logging) datasets were each assembled separately using Trinity.

#### Illumina-oases/velvet

The 179148376 Illumina reads of length 90 from the 2nd and 3rd replicates of the RNASeq experiments were assembled using Velvet 1.2.03 followed by Oases 0.2.06 [86] with parameters ‘-min_trans_lgth 200’ and ‘-cov_cutoff auto’. Multiple values of k were investigated.

### Clustering of the transcriptomes

The three resulting assemblies, namely 454Isotigs, Illumina-Trinity, Illumina-Oases/Velvet, were analyzed separately as was a merge of all 3 data sets (Merge). CDHIT [87], Usearch [88] and TGICL [89] were used to cluster the Merge assembly and the Trinity assembly. Based on performance, TGICL was chosen for the clustering. With TGICL either the default parameters were used or various percent identities were employed as reported. Usearch version 5.2.32 was used with various percentage identities as reported. Cd-hit-est (version 4.5.6-2011-09-02 was used with options ‘–r 1 –M 10000 –T 6 –d 0 –n 5’ as well as several values for the percent identity (option ‘–c’). After preliminary analysis, two transcriptomes were pursued—the Extended transcriptome (the Merge assembly clustered with TGICL) and the 454-Newbler (the Newbler assembly unclustered).

### Coverage tests

Genomes and Transcriptomes were compared using blastx with E-value 1e-10. The method frac_aligned of the MapTiling Bioperl module was used to estimate the fraction of the tef Sanger sequencing and abiotic stress genes that were aligned to the genome and transcriptomes. The frac_aligned method returns the percentage of the query sequence length that is aligned. As this method filters out any hit with high-scoring segment pairs *(*HSPs*)* in more than one context (plus or minus), the coverages generated are underestimates of the total coverage.

### Proteome prediction

Prediction of coding regions of the transcriptomes was accomplished using ESTScan [90] (with m = -100, N = 0, w = 60, and using the *Zea mays* scoring matrix).

### Sequence comparison

The following databases were obtained from Phytozome [91]: *Sorghum bicolor* (Phytozome, v. 79) [40], *Zea mays* (Phytozome, v. 181) [92], Setaria italica (Phytozome, v. 164) [31, 93] and *Brachypodium distachyon* (Phytozome, v. 192) [94]. The *Oryza sativa* genome was retrieved from IRGSP (version 1.0, 2011-12-05) [95, 96].

### Phylogenetic trees

#### Waxy tree

Partial sequences of the WAXY gene from barley (*Hordeum vulgare*, X07931), bread wheat (*Triticum aestivum*, KF861808), finger millet (*Eleusine coracana*, AY508652), foxtail millet (*Setaria italica*, AB089143), maize (*Zea mays*, EU041692), *Paspalum simplex* (AF318770), pearl millet (*Pennisetum glaucum*, AF488414), proso millet (*Panicum miliaceum*, GU199268), rice (*Oryza sativa*, FJ235770.1), sorghum (*Sorghum bicolor*, EF089839), and tef (*Eragrostis tef*, AY136939) were obtained from the NCBI database. The WAXY sequences were aligned using Mafft (L-INS-I) [97] with the default settings [98, 99]. PhyML [100] was used to obtain a maximum likelihood tree using the default model of HKY85 + G. Branch support was inferred using the Shimodaira–Hasegawa-like (SH) aLRT provided by PhyML. Trees were visualized using FigTree v1.3.1 [101].

#### Supergene tree

OrthoMCL [102] was used to find homologous gene families between the transcriptome and the entire set of available grass genomes (sorghum, rice, Brachypodium, Setaria and maize). The default parameters (blastp E-value < = 10^-5^ were used. Families with only one ortholog per genome were found and their sequences were aligned for each family. Gapped regions at the beginning and at the end of the alignment were trimmed. The aligned sequences were then concatenated to form ‘supergenes’ from which the phylogeny and branch lengths were inferred.

#### SSR tree

The phylogeny of the SSR sequences was computed with DNAPars from the Phylip package [103] implemented on the T-rex server [104] and visualized with Archaeopteryx [105].

#### Prolamin tree

The sequences of the prolamins are based on Xu and Messing [74] and were downloaded from NCBI. The accession numbers of the sequences used are: ABF99054.1, ACN58179.1, ACM68402.1, 2ACM68402.1, XP_004965361.1, ABP64790.1, NP_001105054.1, AAW32936.1, NP_001105056, NP_001145560, NP_001142525, ABP64793, NP_001105058, CAA39254.1, AAW82166.1, ACJ03469.1, gi_6983787, ABY71029.1, AAA32955.1, AEG74428.1, ACF93465.1, AAV86085.1, CAA37729.1, 21833173, Sb02g025490, AAS73289.1, NP_001105337.1, ADD98895.1, 023603 m, AAS67324.1, AAP80642.1, AAP31050.1, ACR61557.1, AAT93857.1, EES19761.1. The phylogeny of the prolamins was computed using PhyML [100] as implemented on phylogeny.fr [106] and visualized using Archaeopteryx [105].

### Sequence analysis

All multiple sequence alignments were performed using Mafft v6.903b [97] unless otherwise specified. Nucleotide-peptide matches were made with the NucPepMatch package of the Darwin software system [107]. EMBOSS version 6.3.1 was used for sequence manipulation [108].

### Estimation of genome size

The distribution of k-mer frequencies was estimated with jellyfish [37] using 100 bp reads from the 300 bp insert-size library. Jellyfish count was run on fastq files converted to fasta files and options ‘-s 1000000000-C -c 4’. Jellyfish merge and histo were used to merge the count files and create a histogram, respectively. The maximum of this distribution is related to the sequencing depth (N), read length (L) and kmer length(K) via M = N * (L – K + 1)/L. The total sequencing depth divided by the real sequencing depth is an estimate of the genome size. The integer value of M from the distribution was used.

### SSR detection

The MISA perl script [109] was used to identify SSRs with the default settings: searching for repeats of mononucleotides that occur more than 10 times, dinucleotides that occur more than six times, and nucleotides of lengths 3–6 which occur more than five times. Perl scripts provided on the MISA website to interface MISA with Primer3 [53, 54] were used to design primers for all SSRs with repeat size of three or more (Additional file 6: Table S18 for the entire genome).

### Annotation

Annotation was performed using the Praise internal automated annotation platform of the Swiss Institute of Bioinformatics, a system designed to provide high quality merging of automatic annotations using manually curated rules. It includes annotation extracted from HAMAP [110], PROSITE [111], transmembrane regions, signal peptides as well as the UniProt Feature Table (FT), comments (CC), descriptions (DE) and keywords (KW). It propagates detailed functional annotation (e.g. active site positions) derived from these sources, resolves redundant or conflicting predictions and aggregates all generated annotations into UniProtKB/Swiss-Prot format entries. What could not be annotated by Praise, was then annotated by more traditional method of blasting against UniProt [112], InterPro [113] and PRIAM [114]. Blasting was performed with an E-value of 1e-09.

### Abiotic stress gene analysis

The sequences of 26 genes implicated in abiotic stress in *Arabidopsis thaliana, Oryza sativa* or *Sorghum bicolor* were downloaded from NCBI and used to find the protein sequence of the sorghum homolog from the proteome of *Sorghum bicolor* (Phytozome, version 79) using blastx. Then tblastn was used to search each sorghum abiotic stress protein sequence in the genome and transcriptomes of *Eragrostis tef, Zea mays, Sorghum bicolor, Oryza sativa, Brachypodium distrachyum* and *Setaria italica* using a blast E-value of 1e-05. The number of copies found with length greater than or equal to 70% of the length of the query sequence was recorded.

### Analysis of gluten-related genes

Gluten epitopes from wheat (*Tritium aestivum*), barley (*Hordeum vulgare*) and rye (*Secale cereal*) found in Tye-Din *et al.* [5] were sought in the Maker-predicted protein sequences of tef (*Eragrostis tef*), Brachypodium (*Brachypodium distrachyum*) version 192, barley (*Hordeum vulgare*) MIPS vesion 23 March 2012, rice (*Oryza sativa*) IRGSP version 1.0, 2011-12-05, sorghum (*Sorghum bicolor*) Phytozome version 79, foxtail millet (*Setaria italica*) Phytozome version 164 and maize (*Zea mays*) Phytozome version 181 using MUMmer 3.0 [115]. Exact matches of the 20-amino acid oligopeptide epitopes, core 16-amino acid oligopeptide epitopes, core 13-amino acid oligopeptide epitopes, core 12-amino acid oligopeptide epitopes and core 11-amino acid oligopeptide epitopes were counted. Gluten epitopes were searched in rye (*Secale cereal,* taxid:4550) and wheat (*Tritium aestivum,* taxid:4565) using blastP from NCBI BLAST [116] as the whole genomes are unavailable. Nucleotide-peptide matches were obtained with the Darwin [107] bioinformatics platform.

### Visualizazion

The circular plot showing the correspondance betweeen the genetic and physical maps was created with Circos [117]. The comparison of orthologous syntenic genomic regions of the SAL1 gene was created with CoGe [43, 45] as was the dotplot of the syntenic map in Figure 2. Trees were visualized using FigTree v1.3.1 [101]. Gepard [46] was used to create the dotplot of the A and B genomes.

### Availability of supporting data

Data, analyses and updates can be downloaded from: http://www.tef-research.org/genome.html.

The genome and maker annotations are available for viewing, blasting and download at CoGe at: https://genomevolution.org/CoGe/GenomeInfo.pl?gid=22790.

The project is registered at NCBI with BioProject Number: http://www.ncbi.nlm.nih.gov/bioproject/253673.

The sample accessions are: SAMN02872936 (for GNY7); SAMN02872919 (GNY8); GNY9 and SAMN02872920 (GNY9).

Accession numbers in the Sequence Read Archive are: SRR1463375, SRR1463376, SRR1463377, SRR1463396, SRR1463397 and SRR1463402.

## Electronic supplementary material

Additional file 1: Figure S1: Alignment of tef *KO2* A and B copies. (DOCX 21 KB)

Additional file 2: **A file with Supplementary Notes, Supplementary Figures and Supplementary Tables and references.** (PDF 802 KB)

Additional file 3: Table S7: Location of SSR markers in the tef genome. (XLSX 84 KB)

Additional file 4: Figure S4: Alignment of A and B genomes. (DOCX 2 MB)

Additional file 5: Table S12: Location of tef CNLT markers in the pseudo-chromosomes of tef ordered by linkage group. (XLSX 38 KB)

Additional file 6: Table S18: List of selected SSRs identified from scaffolds. (TXT 5 MB)

## Abbreviations

aLRT: Approximate likelihood ratio test
bp: Base pair
BLAST: Basic local alignment search tool
CC: Comments
CDS: Coding sequence
CoGe: Comparative genomics platform
DE: Descriptions
ERD1: Early responsive to dehydration stress 1
EST: Expressed sequence tags
FT: Feature table
Gbp: Giga base pair
GBS: Genotyping by sequencing
GI: Glycemic index
GWAS: Genome wide association studies
HSPs: high-scoring segment pairs
IRGSP: International rice genome sequencing project
Ka: Non-synonymous substitution rate
kbp: Kilo base pair
Ks: Synonymous substitution rate
KW: Key word
LMW: Low-molecular-weight subunit
Mbp: Mega base pair
MIPS: Munich information center for protein sequences
MISA: MIcro SAtellite identification tool
MITEs: Miniature inverted-repeat transposable elements
ML: Maximum likelihood
MYA: Million years ago
NCBI: National Center for Biotechnology Information
PAML: Phylogenetic analysis by maximum likelihood
PRIAM: PRofils pour l’Identification Automatique du Métabolisme (enzyme-specific profiles for metabolic pathway prediction)
SGR: Stay green rice
SSR: Simple sequence repeats
TE: Transposable elements
TGICL: TIGR Gene indices clustering tools
TREP: Triticeae repeat sequence database.
