# Supplementary material for: Genome and transcriptome sequencing identifies breeding targets in the orphan crop tef (Eragrostis tef)
Source: BMC Genomics. 2014 Jul 9;15(1):581. doi: 10.1186/1471-2164-15-581 (PMC4119204; doi:10.1186/1471-2164-15-581)
Supplement: Supplementary file 1 — Additional file 1: Figure S1: Alignment of tef KO2 A and B copies. (DOCX 21 KB) [file 12864_2014_6309_MOESM1_ESM.docx]

CLUSTAL format alignment by MAFFT FFT-NS-i (v6.903b)

KO2_A --------------------aatcgtaatttctatcgtcagaagtctaaaaatgctgtga

KO2_B --------------------aatcataatttcaatcaactgaggtctaaaaatgctgtga

scaffold13666 aattcgtgctcttgaatgtaaatcataatttcaatcaactgaggtctaaaaatgctgtga

scaffold8186 actttgtgttcttgaatgcaaatcgtaatttctatcgtcagaagtctaaaaatgctgtga

****.*******:***.:*:**.*****************

1 1 11 1

KO2_A ctgcacaaatttcagaaacaattccgggaa------------------------------

KO2_B ctgcacaaa-ttcagaaacaattccgggac------------------------------

scaffold13666 ctgcacaaa-ttcagaaacaattccgggactggagaaaatccagtgtatttgatgagctt

scaffold8186 ctgcacaaatttcagaaacaattccgggaa------------------------------

********* *******************.

1 1

KO2_A ------------------------------------------------------------

KO2_B ------------------------------------------------------------

scaffold13666 ttttcttcaaccaattgatctggactatggatgtttgattttcaaatatacactgaaaca

scaffold8186 ------------------------------------------------------------

KO2_A ------------------------------------------------------------

KO2_B ------------------------------------------------------------

scaffold13666 catataagttagttcttcttagggtgcagcaacagtacttctcagtggtcatggtttgtg

scaffold8186 ------------------------------------------------------------

KO2_A ------------------------------------------------------------

KO2_B ------------------------------------------------------------

scaffold13666 ttgcaaaactcaaggactatgatgttgttgaaatgtcttgtgtccgaccaaaaaacaaat

scaffold8186 ------------------------------------------------------------

KO2_A ------------------------------------------------------------

KO2_B ------------------------------------------------------------

scaffold13666 agaactcaagcttcagtcccatcttttgcaggcaatgattgcaaagttctcatccatatc

scaffold8186 ------------------------------------------------------------

KO2_A ------------------------------------------------------------

KO2_B ------------------------------------------------------------

scaffold13666 tactcggaagctatccagagcattgacagtgcttactcgtgacaaaactatggttgctac

scaffold8186 ------------------------------------------------------------

KO2_A ------------------------------------------------------------

KO2_B ------------------------------------------------------------

scaffold13666 aagcgactatggtgacttccacaaaatggtaaagcgttatattatgacgagcatgctggg

scaffold8186 ------------------------------------------------------------

KO2_A ------------------------------------------------------------

KO2_B ------------------------------------------------------------

scaffold13666 tacttctggccaggtattgatcacgacaacttttaacaccatacagtttcactttgtgtt

scaffold8186 ------------------------------------------------------------

KO2_A ------------------------------------------------------------

KO2_B ------------------------------------------------------------

scaffold13666 cttgaatgcaaatcgtaatttctatcgtcagaagtctaaaaatgctgtgactgcacaaat

scaffold8186 ------------------------------------------------------------

KO2_A --------------------acgagaaacaggatggttgatagtatgttaagcacttttc

KO2_B --------------------acgagaaacaggatggttgatagtatgttaagcacttttc

scaffold13666 ttcagaaacaattccgggaaacgagaaacaggatggttgatagtatgttaagcacttttc

scaffold8186 --------------------acgagaaacaggatggttgatagtatgttaagcacttttc

****************************************

KO2_A aatcaatgttgactgatgatccaaatactcctctgaatttccgggaagttttcaagaatg

KO2_B aatcaatgttgactgatgatccaaatactcctctgaatttccgggaagttttcaagaatg

scaffold13666 aatcaatgttgactgatgatccaaatactcctctgaatttccgggaagttttcaagaatg

scaffold8186 aatcaatgttgactgatgatccaaatactcctctgaatttccgggaagttttcaagaatg

************************************************************

KO2_A agctattccgactatctttgattcaagtaagtccacttttgttgcgcttattcgtaatgc

KO2_B agctattccgactatctttgattcaagtaagtccacttttgttgtgcttatccgtaatgc

scaffold13666 agctattccgactatctttgattcaagtaagtccacttttgttgcgcttattcgtaatgc

scaffold8186 agctattccgactatctttgattcaagtaagtccacttttgttgcgcttattcgtaatgc

******************************************** ****** ********

2 2

KO2_A ttcataagtgctagcaatattcacagttactctagctaagaactttgataaggtcttctg

KO2_B ttcataagtgctagcaatattcacagttactctagctaagaactttgataaggtcttctg

scaffold13666 ttcataagtgctagcaatattcacagttactctagctaagaactttgataaggtcttctg

scaffold8186 ttcataagtgctagcaatattcacagttactctagctaagaactttgataaggtcttctg

************************************************************

KO2_A taaacactataattgtaaaaatgatttattcttatgtttctaaactgagaatgtgcactg

KO2_B taaacactataattgtaaaaatgatttattcttatgtttctaaactgagaatgtgcactg

scaffold13666 taaacactataattgtaaaaatgatttattcttatgtttctaaactgagaatgtgcactg

scaffold8186 taaacactataattgtaaaaatgatttattcttatgtttctaaactgagaatgtgcactg

************************************************************

KO2_A caattttaggctttaggcgacgatgtaagttcagtctatgtggaagagtttgggaaggtc

KO2_B caattttaggctttaggcgacgatgtaagttcagtctatgtggaagagtttgggaaggtc

scaffold13666 caattttaggctttaggcgacgatgtaagttcagtctatgtggaagagtttgggaaggtc

scaffold8186 caattttaggctttaggcgacgatgtaagttcagtctatgtggaagagtttgggaaggtc

************************************************************

KO2_A ttatcgaaggaggaaatctaccaggccactgtggttgatatgatgatgtgtgctattgag

KO2_B ttatcgaaggaggaaatctaccaggccactgtggttgatatgatgatgtgtgctattgag

scaffold13666 ttatcgaaggaggaaatctaccaggccactgtggttgatatgatgatgtgtgctattgag

scaffold8186 ttatcgaaggaggaaatctaccaggccactgtggttgatatgatgatgtgtgctattgag

************************************************************

KO2_A gtcgactggagggacttcttcccatacctccgttggattccaaataggagctttgagaca

KO2_B gtcgactggagggacttcttcccatacctccgttggattccaaataggagctttgagaca

scaffold13666 gtcgactggagggacttcttcccatacctccgttggattccaaataggagctttgagaca

scaffold8186 gtcgactggagggacttcttcccatacctccgttggattccaaataggagctttgagaca

************************************************************

KO2_A agagtactgactacagaagcaaggcggactgcagtgatgcgagccttgatcaacgagcaa

KO2_B agagtactgactacagaagcaaggcggactgcagtgatgcgagccttgatcaacgagcaa

scaffold13666 agagtactgactacagaagcaaggcggactgcagtgatgcgagccttgatcaacgagcaa

scaffold8186 agagtactgactacagaagcaaggcggactgcagtgatgcgagccttgatcaacgagcaa

************************************************************

KO2_A aagaaaagaattgaacgtggcgaggtaacccaacagcatagtttttacagttttgtttct

KO2_B aagaaaagaattgaacgtggcgaggtaacacaacggcatagtttttacagctttgtttct

scaffold13666 aagaaaagaattgaacgtggcgaggtaacccaacagcatagtttttacagttttgtttct

scaffold8186 aagaaaagaattgaacgtggcgaggtaacccaacagcatagtttttacagttttgtttct

*****************************.****.*************** *********

2 2 2

KO2_A ttgtttggagagaaaacaaaaatgttgttcttgagttcttgactcaataaatccaatgca

KO2_B ttgtttggagag-aaacaaaaatgtt-------aattcttgactcaataaatccaatgca

scaffold13666 ttgtttggagagaaaacaaaaatgttgttcttgagttcttgactcaataaatccaatgca

scaffold8186 ttgtttggagagaaaacaaaaatgttgttcttgagttcttgactcaataaatccaatgca

************ ************* *.*************************

2 2222222 2

KO2_A ggctagaatatgttatctggacttcctgttagcagagaatacgctaaccgatgaacagtt

KO2_B ggcgagaatatcctatctggacttcctgttagcagagaatacgctaaccgatgaacagtt

scaffold13666 ggctagaatatgttatctggacttcctgttagcagagaatacgctaaccgatgaacagtt

scaffold8186 ggctagaatatgttatctggacttcctgttagcagagaatacgctaaccgatgaacagtt

*** ******* ***********************************************

2 22

KO2_A ggtaatgctggtgtgggagtcagtcatagaagctgcagatactactttggtcacgactga

KO2_B ggtaatgctggtgtgggagtcagtcatagaagctgcagatacaactttggtcacgactga

scaffold13666 ggtaatgctggtgtgggagtcagtcatagaagctgcagatacaactttggtcacgactga

scaffold8186 ggtaatgctggtgtgggagtcagtcatagaagctgcagatacaactttggtcacgactga

******************************************:*****************

KO2_A gtgggccatgtatgagatcgcgaagcacccagaaaaacaagtaagtgccgaaacttgttt

KO2_B gtgggccatgtatgagatcgcgaagcacccagagaaacaagtaa--gctgaaacttgttt

scaffold13666 gtgggccatgtatgagatcgcgaagcacccagagaaacaagtaa--gctgaaacttgttt

scaffold8186 gtgggccatgtatgagatcgcgaagcacccagaaaaacaagtaagtgccgaaacttgttt

*********************************.********** ** ***********

1 11 1

KO2_A cctattttactgatagtatcagactgttaacttcatttcttttccactccaggatcgcct

KO2_B cctagtttactgatagtatcagactgttaacttcgtttcttttccactccaggatcgcct

scaffold13666 cctagtttactgatagtatcagactgttaacttcgtttcttttccactccaggatcgcct

scaffold8186 cctattttactgatagtatcagactgttaacttcatttcttttccactccaggatcgcct

**** *****************************.*************************

1 1

KO2_A tttccaggaaatccaagaagtc--------------------------------------

KO2_B tttccaggaaatccaagaagtt--------------------------------------

scaffold13666 tttccaggaaatccaagaagtttgtggcaacgagaagactgttaacttcgtttcttttcc

scaffold8186 tttccaggaaatccaagaagtc--------------------------------------

*********************

1

KO2_A -------------------------------------tgtggcaacgagacagttactga

KO2_B -------------------------------------tgtggcaacgagacagtcactga

scaffold13666 actccaggatcgccttttccaggaaatccaagaagtctgtggcaacgagacagttactga

scaffold8186 -------------------------------------tgtggcaacgagacagttactga

***************** *****

2

KO2_A ggatcatttgccacggctggtttacttgaacgccgtgttccatgagaccctgaggcggca

KO2_B ggatcacttgccacggctggtttacttgaacgccgtgttccatgagaccctgaggcggca

scaffold13666 ggatcatttgccacggctggtttacttgaacgccgtgttccatgagaccctgaggcggca

scaffold8186 ggatcatttgccacggctggtttacttgaacgccgtgttccatgagaccctgaggcggca

****** *****************************************************

2

KO2_A tgctccagttccgctagtgcctccaagatttatccacgagaataccacattggctggcta

KO2_B tgctccagttccgctggtgcctccaagatttatccatgagaacaccaccttggctggcta

scaffold13666 tgctccagttccgctggtgcctccaagatttatccatgagaacaccaccttggctggcta

scaffold8186 tgctccagttccgctggtgcctccaagatttatccacgagaataccacattggctggcta

***************.******************** ***** *****.***********

3 1 1 1

KO2_A cgacgtaccagctggcacagaggtacgcacaacagtatcggcaagctacatgttacatat

KO2_B tgacgtaccggctggcacagaggtacgcacaaccgtatcagcaacctatatgttacatat

scaffold13666 tgacgtaccggctggcacagaggtacgcacaaccgtatcagcaacctatatgttacatat

scaffold8186 cgacgtaccagctggcacagaggtacgcacaacagtatcggcaagctacatgttacatat

********.***********************.*****.**** *** ***********

3 1 1 1 1

KO2_A aagctattccattacacaattttaaccatgaataatggagtctaaagcaatggcaattat

KO2_B atgctatttcattacataattttaaccatgaataatggagtctaaagcaatggcaattgt

scaffold13666 atgctatttcattacataattttaaccatgaataatggagtctaaagcaatggcaattgt

scaffold8186 aagctattccattacacaattttaaccatgaataatggagtctaaagcaatggcaattat

*:****** ******* *****************************************.*

1 1 1 1

KO2_A ggtagatggtcatcaacctgtatggatgcaacatgaacaagaacgattgggacgaacccg

KO2_B gctagatgatcatcaacctgtatggatgcaacatgaacaagaacgattgggatgaacccg

scaffold13666 gctagatgatcatcaacctgtatggatgcaacatgaacaagaacgattgggatgaacccg

scaffold8186 ggtagatggtcatcaacctgtatggatgcaacatgaacaagaacgattgggacgaacccg

* ******.******************************************* *******

1 1 1

KO2_A aggaatggaagccagaaaggtttctggatgggaggtttgaggctgcagacatgtacaaga

KO2_B aggaatggaagccagaaaggtttctggatgggaggtttgaggctgcagacatgtacaaga

scaffold13666 aggaatggaagccagaaaggtttctggatgggaggtttgaggctgcagacatgtacaaga

scaffold8186 aggaatggaagccagaaaggtttctggatgggaggtttgaggctgcagacatgtacaaga

************************************************************

KO2_A ctatggccttcggtgctggaaggagggcctgtgccggcagcatgcaggcgatgaacatct

KO2_B cgatggccttcggtgctggaaggagggcctgtgctggcagcatgcaggcgatgaacatct

scaffold13666 cgatggccttcggtgctggaaggagggcctgtgctggcagcatgcaggcgatgaacatct

scaffold8186 ctatggccttcggtgctggaaggagggcctgtgccggcagcatgcaggcgatgaacatct

* ******************************** *************************

1 1

KO2_A cgtgcatgtccatcgcgaggttcgtgcaagagttcgagtggaggctcaaggaagg-----

KO2_B cgtgcatgtccatcgcgaggtttgtgcaagagttcgagtggaggctcaaggaagg-----

scaffold13666 cgtgcatgtccatcgcgaggtttgtgcaagagttcgagtggaggctcaaggaaggcgacg

scaffold8186 cgtgcatgtccatcgcgaggttcgtgcaagagttcgagtggaggctcaaggaaggcgacg

********************** ********************************

1 11111

**Supplementary Figure S1. *Alignment of KO2 tef A and B copies from Sanger sequencing with scaffolds from the genomic assembly*.** The KO2 A and B copies were obtained from Sanger sequencing of tef and compared to the scaffolds obtained from the tef genome assembly. Positions marked with a star are identical in all tef sequences. At the beginning and end of the alignment scaffold8186 is identical to KO2_A while scaffold3666 is identical to KO2_B, these positions are marked with a “1”. At positions marked with a 2, both scaffolds are identical to KO2_A. In very few positions, marked with a “3” both scaffolds are identical to KO2_B. One scaffold has two long inserts not present in the Sanger sequence (yellow).
