# Supplementary material for: Genome and transcriptome sequencing identifies breeding targets in the orphan crop tef (Eragrostis tef)
Source: BMC Genomics. 2014 Jul 9;15(1):581. doi: 10.1186/1471-2164-15-581 (PMC4119204; doi:10.1186/1471-2164-15-581)
Supplement: Supplementary file 2 — Additional file 2: A file with Supplementary Notes, Supplementary Figures and Supplementary Tables and references. (PDF 802 KB) [file 12864_2014_6309_MOESM2_ESM.pdf]

Supplementary Material 1

Genome and transcriptome sequencing identifies breeding targets in the orphan crop tef (*Eragrostis tef*)

Gina Cannarozzi[1,2], Sonia Plaza-Wüthrich[1], Korinna Esfeld[1], Stéphanie Larti[1*], Yi Song Wilson[1], Dejene Girma[1,3], Edouard de Castro[4], Solomon Chanyalew[5], Regula Blösch[1], Laurent Farinelli[6], Eric Lyons[7], Michel Schneider[4], Laurent Falquet[2+], Cris Kuhlemeier[1], Kebebew Assefa[5] and Zerihun Tadele[1§]

[1]University of Bern, Institute of Plant Sciences, Altenbergrain 21, CH-3013 Bern, Switzerland

[2]Swiss Institute of Bioinformatics, Vital-IT, Quartier Sorge - Batiment Genopode, 1015 Lausanne, Switzerland

[3]Ethiopian Institute of Agricultural Research, National Biotechnology Laboratory (Holetta), P.O.Box 2003, Addis Ababa, Ethiopia

[4]Swiss Institute of Bioinformatics, Rue Michel-Servet 1, 1211 Geneva 4, Switzerland

[5]Ethiopian Institute of Agricultural Research, Debre Zeit Agricultural Research Center, P.O.Box 32, Debre Zeit, Ethiopia

[6]FASTERIS SA, Ch. du Pont-du-Centenaire 109, P.O. Box 28, CH-1228 Plan-les-Ouates, Switzerland

[7]Univerisity of Arizona, School of Plant Sciences, 1140 E. South Campus Drive, P.O. Box 210036, 303 Forbes Building, Tucson, AZ 85721-0036, USA

[§]Corresponding author

[*]Current address: University of Bern, Clinic for Parodontology, Freiburgstrasse 7, CH-3010 Bern, Switzerland

[+]Current address: University of Fribourg, Faculty of Science, Ch. du Musée 10, CH-1700 Fribourg, Switzerland

Table of Contents

Supplementary Notes

Supplementary Note 1. PCR Amplification of two amplicons from selected scaffolds
Supplementary Note 2. Comparison of tef genome to sorghum genome
Supplementary Note 3. Annotation
Supplementary Note 4. Abiotic stress
Supplementary Note 5. Gluten related genes

Supplementary Figures

Supplementary Figure S1. Alignment of tef KO2 tef A and B copies from Sanger & genome
Supplementary Figure S2. Length distribution of proteins predicted in the genome & transcriptomes
Supplementary Figure S3. Distribution of k-mer frequency in the raw sequencing reads ***Supplementary***
Supplementary Figure S4. Alignment of A and B genomes
Supplementary Figure S5. Allelic variation at a novel SSR locus found in tef
Supplementary Figure S6. Discovery of a novel SSR marker in tef and *Eragrostis* species
Supplementary Figure S7. Phylogenetic tree of natural accessions and improved varieties of tef
Supplementary Figure S8. Tandem duplications of SAL1 gene confirmed by Sanger sequencing

Supplementary Tables

Supplementary Table S1. Summary of genome sequencing data for the tef genome
Supplementary Table S2. Summary of sequencing data for the tef transcriptome
Supplementary Table S3. Summary of assembly statistics for tef the tef transcriptome
Supplementary Table S4. Percentage of genes and bases found in tef transcriptome and genome
Supplementary Table S5. Summary of assembly statistics for the tef and other genomes
Supplementary Table S6. Reads mapped to the genome assembly scaffolds greater than 1000 bp
Supplementary Table S7. Location of SSR markers in the tef genome
Supplementary Table S8. Amplification of scaffolds between CNLT markers via Sanger sequencing
Supplementary Table S9. Comparison between scaffolds and corresponding sequences from Sanger
Supplementary Table S10. Comparison between Sanger sequencing and NGS sequencing
Supplementary Table S11. Primers used to isolate agronomically important genes in tef
Supplementary Table S12. Location of tef CNLT markers in the pseudo-chromosomes
Supplementary Table S13. Divergence dates in selected grass species estimated from modal Ks values
Supplementary Table S14. Identity between aligned segments of tef A and B pseudo-chromosomes
Supplementary Table S15. Percentage identity between pairs of homeologous gene copies
Supplementary Table S16. Representation of the transcriptome in the genome
Supplementary Table S17. Summary statistics of SSR markers found in the tef genome
Supplementary Table S18. List of 22833 SSRs identified from scaffolds
Supplementary Table S19. Primers for the amplification of a novel SSR marker
Supplementary Table S20. Number of annotations found by various tools
Supplementary Table S21. Representation of abiotic stress related genes in the tef genome
Supplementary Table S22. Abiotic stress genes and their numbers in grass genomes
Supplementary Table S23. Presence of gluten epitopes and their amounts in grass genomes
Supplementary Table S24. Summary of prolamin genes found in the tef genome and transcriptomes.

Supplementary Notes

Supplementary Note 1. ***PCR Amplification of amplicons from selected scaffolds***. Three long-range PCR amplifications were made based on information from scaffolds followed by Sanger sequencing. Among these, two of them contain the region between SSR markers previously reported by Zeid *et al*. [1]. These markers were sought in the tef genome using the Darwin software system's SearchSeqDb for exact matching [2]. Two scaffolds with two CNTL markers less than 10000 bp apart were chosen for amplification between the markers. In order to amplify the part of the scaffold containing the markers, each one was subdivided into three overlapping fragments. The third long-range PCR was made in the region where three tandem duplications of SAL1 gene were identified. Each fragment was amplified by PCR, purified using the NucleoFast 96 PCR protocol provided by the manufacture (Macherey-Nagel, Oensingen, Switzerland) and sequenced (Microsynth AG, Balgach, Switzerland). The PCR reaction, in 20 μL, contained approximately 200 ng of template genomic DNA, 1x GoTaq PCR buffer (1.5 mM $MgCl_2$), 0.375 μM of each primer (forward and reverse), 0.2 mM of each dNTPs, and 1U of GoTaq polymerase (Promega, Madison, USA). Thermocycling started with a denaturation step for 2 min at 94 °C followed by 40 cycles of 20 s at 94 °C, 20 s at the appropriate annealing temperature, an elongation time between 1min 30s and 4min at 72 °C (Supplementary Table 8), and stopped after a final extension step of 72 °C for 10min.

PCR Amplification of selected SSR markers. DNA extraction: DNA was extracted following a cetyltrimethylammonium bromide (CTAB) extraction [3] when the plants were one month old. Marker amplification and detection by a polyacrylamide gel: A SSR marker was amplified from two tef ecotypes (Tsedey and Alba) with primers designed from the tef genome presented in the present study. In order to reduce genotyping cost, primers were designed based on [4]. The PCR reaction, in 10 μL, contained approximately 200 ng of template DNA, 1 x PCR buffer (1.5 mM MgCl2), 0.35 pmol of the M13-tailed forward primer (5'- CACGACGTTGTAAAACGACCTCATCTCCCACCCTCACTC), 3.50 pmol reverse primer (5'-GGTCGTTTGATCTGGGCTAC), 1.75 pmol labeled (IRD-700/800)

M13 primer (5′ -CACGACGTTGTAAAACGAC). 0.2 mM of each dNTPs, and 0.5 U of GoTaq polymerase (Promega, Dübendorf, Switzerland). Thermocycling started with a denaturation step for 2 min at 94 °C followed by 45 cycles of 20 s at 94 °C, 20 s at 50 °C, and 1 min at 72 °C, and stopped after a final extension step of 72 °C for 10 min. After PCR, samples were denatured by adding 30 μL formamide stained with bromophenol blue. Finally, 0.5 μL of the PCR products were loaded on 7% polyacrylamide gels. Gels pictures were analyzed using the program GelBuddy [5]. Eighteen other tef ecotypes as well as four other Eragrostis species (*E. curvula, E. minor, E. pilosa* and *E. trichodes*) were amplified with the primers M13-tailed forward and reverse used for the marker amplification with the same PCR conditions and the amplicons were sequenced by Sanger method with the M13 primers by Microsynth (Microsynth AG, Balgach, Switzerland).

Sequencing the entire 10 kbp region using Sanger sequencing and then aligning the scaffold to the amplicon resulted in 9,707 aligned nucleotides between CNLTs316 and CNLTs472 on scaffold2429 with 99% sequence identity. Sequencing of the other fragment of length 8,369 bp between CNLTs77 and CNLTs322 on scaffold8420 resulted in an alignment with 97% sequence identity between the tef scaffolds and the corresponding Sanger sequence. The number of N's was often poorly estimated.

Supplementary Note 2. ***Comparison of tef genome to other grasses.*** The tef genome and Maker gene predictions were uploaded to CoGe [6, 7] a platform containing many draft and whole genomes and providing numerous tools for genome alignment, comparison and visualization [8-10]. The SynMap function of CoGe aligns two genomes by using sequence similarity as well as syntenic information. First, putative genes or regions of homology are found between two genomes, then collinear sets of genes are used to infer synteny and syntenic pairs of genes are assigned. These can be used to generate dotplots of homology as in Figure 2 and Supplementary Figure S3. In addition, a host of integrated tools can then be used for genome analysis and visualization. SynMap was run with default settings including the LastZ option for Blastz [11] as well as the following parameters: Minimum number of aligned pairs=5 or 3, Maximum distance between two matches=20, Tandem duplication distance=10.

SynMap first finds regions of high homology using BLAST or Last, a much faster variant of Blast [12]. SynMap identifies collinear putative homologous sequences in two genomes using DAGChainer [13]. The SynMap function was first used to align the tef scaffolds with the *Sorghum bicolor* genome. The tef scaffolds ordered according to the sorghum genome were then downloaded as a list and their sequences joined to form artificial tef “pseudo-chromosomes”. These tef pseudo-chromosomes were used to orient the linkage groups of Zeid [1] in Figure 2 and Supplementary Table S17. Circos was used to generate the plot [14]. The Synmap function of CoGe was used to do pairwise comparisons of the following genomes: *Eragrostis tef* (Coge id 38364; current work), *Sorghum bicolor* (Coge id 38364; [15]), *Zea mays* (Coge id 333; [16]), *Oryza sativa japonica* (Coge id 3; [17]) and *Setaria italica* (Coge id 32546; with CNS PL2.0l v2.1,id2240 [18]) using the default settings.

CodeML of PAML [19] is integrated into CoGe and can be used to estimate the number of synonymous and nonsynonymous substitutions per site (Ks and Ka, respectively) for the complete set of orthologous genes between two genomes. The mode(s) of the distribution of Ks values between two genomes represents either a speciation or a genome duplication event. The ages of the modes of the peaks were estimated using a molecular clock rate of 6.5×10^{-9} synonymous substitutions per synonymous site per year [20]. These estimates can be found in Supplementary Table S16. Additionally, the Maker gene predictions were uploaded to CoGe and can there be visualized and compared to other grasses as shown for the SAL1 gene in Figure 4B. The CoGe URL for this analysis is http://genomevolution.org/r/bsyp.

Supplementary Note 3. *Annotation.* Annotation of the proteins predicted from the transcirptome was performed by the Praise (PRotein Automated annotatIon SystEm) UniProtKB/Swiss-Prot internal automated annotation platform [21]. Praise is an annotation templating system driven by sequence analysis results via manually curated context sensitive annotation templates. Templates (called annotation "rules" - UniRules) are manually curated context sensitive annotation fragments and represent a language that is interpreted by the Praise template engine. It propagates detailed functional

annotation (e.g. active site positions) derived from Prosite and HAMAP motif matches, resolves redundant or conflicting predictions (e.g. for transmembrane) and aggregates all generated annotations into UniProtKB/Swiss-Prot format entries. The Praise platform annotates fewer proteins than systems using a simple BLAST or InterPro-associated GO terms but generates high quality and more numerous annotation "elements" (Supplementary Table S21). All different annotation types were pooled by entry. When excluding non-informative matches (against hypothetical proteins), the percentage or proteins annotated drops to 57%.

Supplementary Note 4. ***Abiotic stress.*** The sequences of 27 genes implicated in abiotic stress in various grasses were downloaded from NCBI and used to find the protein sequence of the *Sorghum biocolor* homolog (Phytozome, version 79) using blastx. Then tblastn was used to search each sorghum abiotic stress protein sequence in the tef genome and transcriptomes of tef (core, extended, 454Isotigs, drought (TrinityGNY11and2), waterlogging (TrinityGNY12and3) and control (TrinityGNY10and1) and other grasses using an e-value of 1e-05. The number of copies found with length greater than or equal to 70% of the length of the query sequence was recorded.

Supplementary Note 5. ***Gluten related genes.*** Gluten epitopes from wheat (*Tritium aestivum*), barley (*Hordeum vulgare*) and rye (*Secale cereal*) [22] were searched in the Maker-predicted protein sequences of tef (*Eragrostis tef*), *Brachypodium* (*Brachypodium distrachyum*) version 192, barley (*Hordeum vulgare*) MIPS version 23 March 2012, rice (*Oryza sativa*) IRGSP version 1.0, 2011-12-05, sorghum (*Sorghum bicolor*) Phytozome version 79 and *Setaria* (*Setaria italica*) Phytozome version 164 and maize (*Zea mays*) Phytozome version 181 using MUMmer 3.0 [23]. Exact matches of the 20-amino acid oligopeptide epitopes, core 16-amino acid oligopeptide epitopes, core 13-amino acid oligopeptide epitopes, core 12-amino acid oligopeptide epitopes and core 11-amino acid oligopeptide epitopes were counted. Gluten epitopes were searched in rye (*Secale cereal*, taxid:4550) and wheat (*Triticum aestivum*, taxid: 4565) using blastP from NCBI BLAST (http://blast.ncbi.nlm.nih.gov/Blast.cgi?) as the full genomic sequences are unavailable.

Supplementary Figures

Supplementary Figure S1. ***Alignment of KO2 tef A and B copies from Sanger sequencing with scaffolds from the genomic assembly.*** The KO2 A and B copies were obtained from Sanger sequencing of tef and compared to the scaffolds obtained from the tef genome assembly. Positions marked with a star are identical in all tef sequences. At the beginning and end of the alignment scaffold8186 is identical to KO2_A while scaffold3666 is identical to KO2_B, these positions are marked with a “1”. At positions marked with a 2, both scaffolds are identical to KO2_A. In very few positions, marked with a “3” both scaffolds are identical to KO2_B. One scaffold has two long inserts not present in the Sanger sequence (yellow). This Figure is provided as a supplementary file [*Supplementary_Material_2_FigS1*]

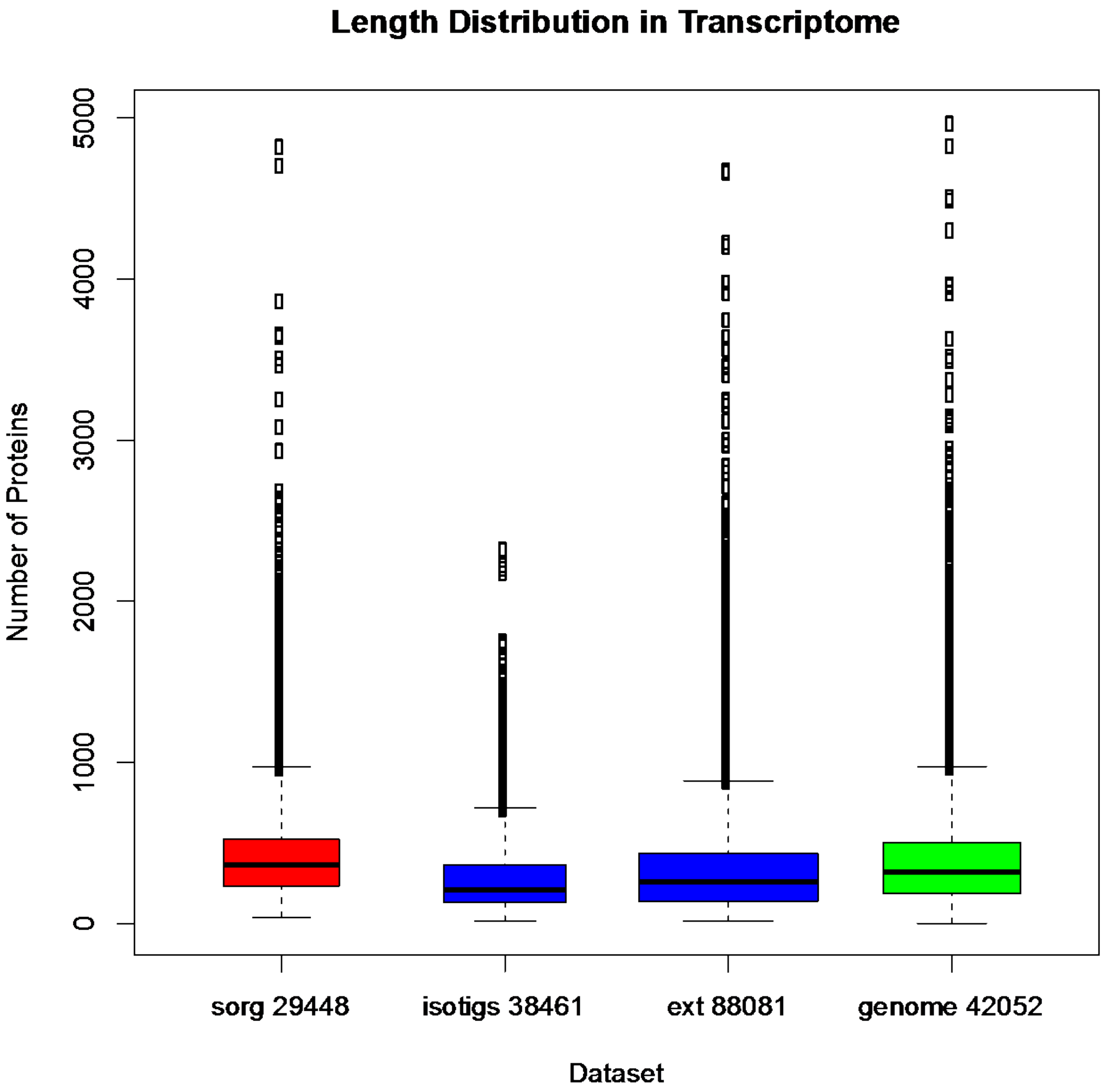

Supplementary Figure S2. ***Distribution of protein lengths in the genome and transcriptomes***. The distribution of the lengths of proteins predicted by ESTscan from the 454Isotigs (isotigs 38461) and the Extended transcriptomes (ext 88081) are compared to the distribution of the lengths of proteins predicted in the genome by the Maker evidence combiner (genome 42052) and to that of sorghum (sorg 29448). The number of proteins in each proteome is indicated.

A

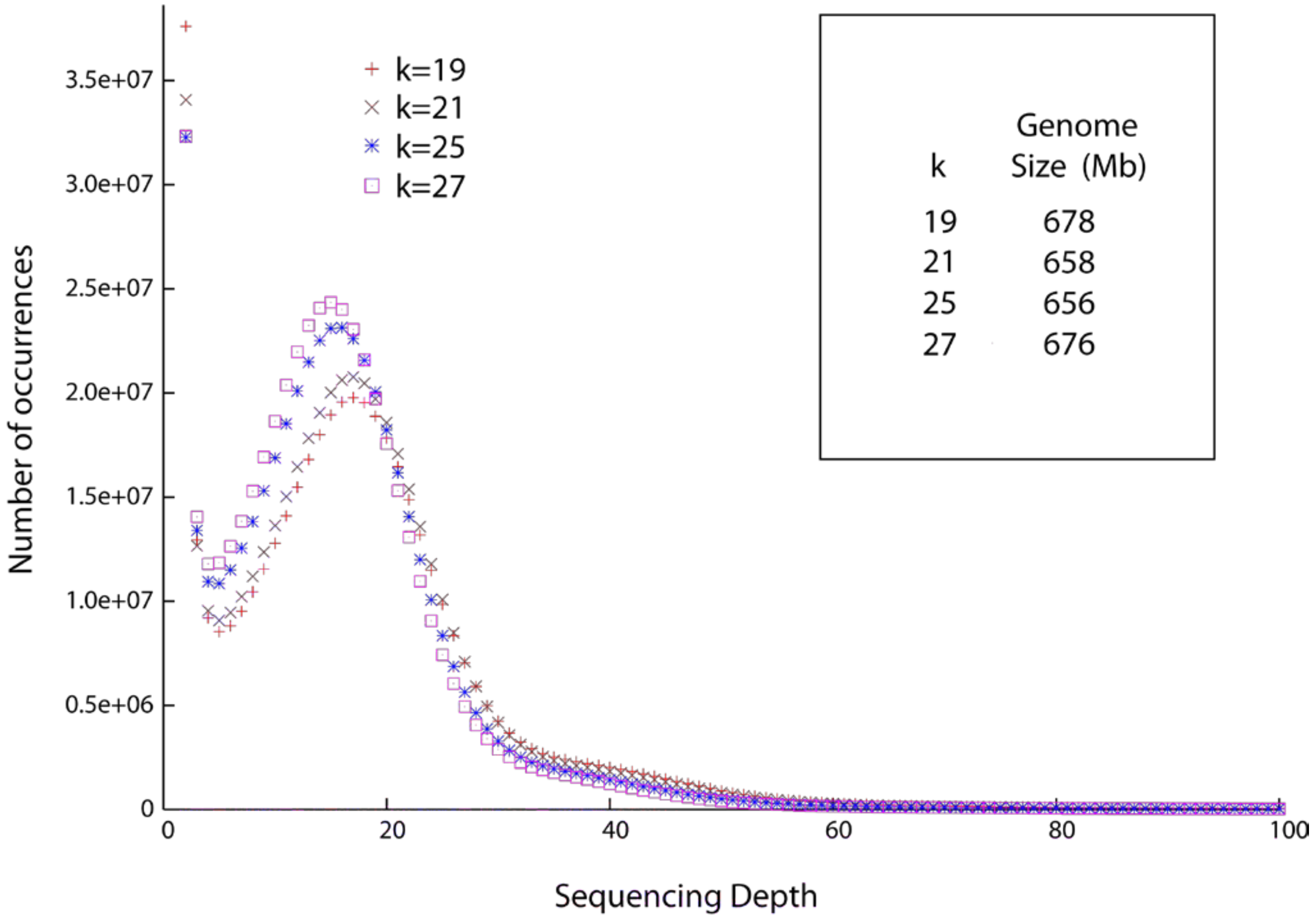

B

k	Genome Size (Mb)
19	678
21	658
25	656
27	676

Supplementary Figure S3. ***Distribution of k-mer frequency in the raw sequencing reads***. A) The distribution of k-mer frequencies was estimated with jellyfish [24] using 85 bp reads from the 300bp insert-size library. The maximum of this distribution (M) is related to the sequencing depth (N), read length (L), and kmer length (K) via M = N * (L – K + 1) / L. The total sequence length divided by the real sequencing depth is an estimate of the genome size. B) Genome size estimates for different k values.

Supplementary Figure S4. ***Alignment of A and B genomes***. Scaffolds were ordered by mapping them to individual sorghum chromosomes and were then sorted into an A and a B genome by sequenctially placing them into two groups based on overlap avoidance. A dotplot shows the correspondence between the A and B genomes. This Figure is provided as a supplementary file [*Supplementary_Material_3_FigS4*] (word file)

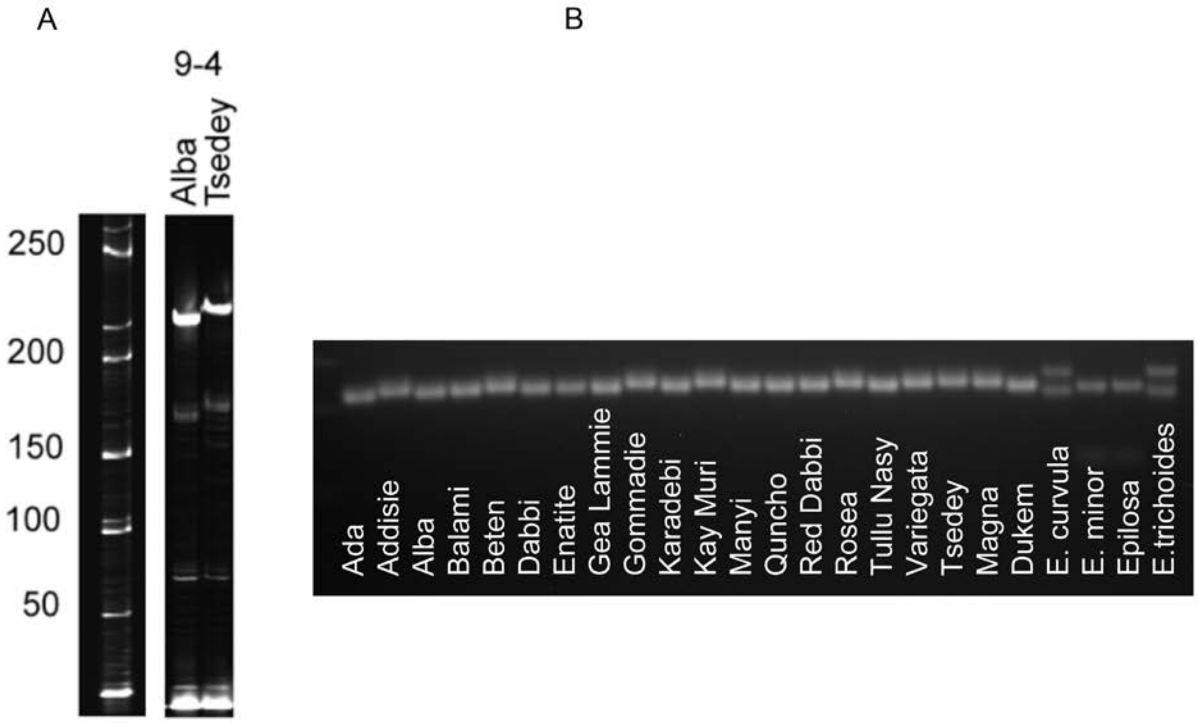

Supplementary Figure S5. ***Allelic variation at a novel SSR locus found in tef***. A) The SSR marker on linkage group 9 has a polymorphism between Alba and Tsedey cultivars of tef. B) The marker was amplified from 20 tef cultivars and four wild *Eragrostis* species.

scaffold2788_17.4	CCTCATCTCC	CACCCTCACT	CACGCCAGCC	GCATTGCACA	GATCGGGACG	G-GCTAGGGT
Addisie	CCTCATCTCC	CACCCTCACT	CACGCCAGCC	GCATTGCACA	GATCGGGACG	G-GCTAGGGT
Beten	CCTCATCTCC	CACCCTCACT	CACGCCAGCC	GCATTGCACA	GATCGGGACG	G-GCTAGGGT
Rosea	CCTCATCTCC	CACCCTCACT	CACGCCAGCC	GCATTGCACA	GATCGGGACG	G-GCTAGGGT
Variegata	CCTCATCTCC	CACCCTCACT	CACGCCAGCC	GCATTGCACA	GATCGGGACG	G-GCTAGGGT
Tsedey	CCTCATCTCC	CACCCTCACT	CACGCCAGCC	GCATTGCACA	GATCGGGACG	G-GCTAGGGT
Magna	CCTCATCTCC	CACCCTCACT	CACGCCAGCC	GCATTGCACA	GATCGGGACG	G-GCTAGGGT
Gommadie	CCTCATCTCC	CACCCTCACT	CACGCCAGCC	GCATTGCACA	GATCGGGACG	G-GCTAGGGT
Kay_Murri	CCTCATCTCC	CACCCTCACT	CACGCCAGCC	GCATTGCACA	GATCGGGACG	G-GCTAGGGT
Ada	CCTCATCTCC	CACCCTCACT	CACGCCAGCC	GCATTGCACA	GATCGGGACG	G-GCTAGGGT
Balami	CCTCATCTCC	CACCCTCACT	CACGCCAGCC	GCATTGCACA	GATCGGGACG	G-GCTAGGGT
Tullu_Nasy	CCTCATCTCC	CACCCTCACT	CACGCCAGCC	GCATTGCACA	GATCGGGACG	G-GCTAGGGT
Alba	CCTCATCTCC	CACCCTCACT	CACGCCAGCC	GCATTGCACA	GATCGGGACG	G-GCTAGGGT
Dabbi	CCTCATCTCC	CACCCTCACT	CACGCCAGCC	GCATTGCACA	GATCGGGACG	G-GCTAGGGT
Enatite	CCTCATCTCC	CACCCTCACT	CACGCCAGCC	GCATTGCACA	GATCGGGACG	G-GCTAGGGT
Gea_Lamie	CCTCATCTCC	CACCCTCACT	CACGCCAGCC	GCATTGCACA	GATCGGGACG	G-GCTAGGGT
Manyi	CCTCATCTCC	CACCCTCACT	CACGCCAGCC	GCATTGCACA	GATCGGGACG	G-GCTAGGGT
Red_Dabi	CCTCATCTCC	CACCCTCACT	CACGCCAGCC	GCATTGCACA	GATCGGGACG	G-GCTAGGGT
Dukem	CCTCATCTCC	CACCCTCACT	CACGCCAGCC	GCATTGCACA	GATCGGGACG	G-GCTAGGGT
Quncho	CCTCATCTCC	CACCCTCACT	CACGCCAGCC	GCATTGCACA	GATCGGGACG	G-GCTAGGGT
Karadebi	CCTCATCTCC	CACCCTCACT	CACGCCAGCC	GCATTGCACA	GATCGGGACG	G-GCTAGGGT
E.pilosa A	CCTCATCTCC	CACCCTCACT	CACACCAGCG	GCATTGCACA	GATCGGGACG	G-GCTAGGGT
E.pilosa B	CCTCATCTCC	CACCCTCACT	CACACCAGCC	GCATTGCACA	GATCGGGACG	G-GCTAGGGT
E.minor	CCTCATCTCC	CACCCTCACT	CACGCCAGCC	GCATTGCACA	GATCGGGACG	G-GCTAGGGT
E.curvula	CCTCATCTCC	CACCCTCACT	CACGCCAGCC	GCATTGCACA	GATCGGGACG	GAGCTAGGGT
E.trichodes	CCTCATCTCC	CACCCTCACT	CACGCCAGCC	GCATTGCACA	GATCGGGACG	GAGCTAGGGT
scaffold2788_17.4	TTGCGATTTA	TGCTCCCTCC	TCTCCTCTCC	TC-TCCTCTC	CTCTTCTCCT	CCAAGCTTTT
Addisie	TTGCGATTTA	TGCTCCCTCC	TCTCCTCTCC	TC-TCCTCTC	CTCTTCTCCT	CCAAGCTTTT
Beten	TTGCGATTTA	TGCTCCCTCC	TCTCCTCTCC	TC-TCCTCTC	CTCTTCTCCT	CCAAGCTTTT
Rosea	TTGCGATTTA	TGCTCCCTCC	TCTCCTCTCC	TC-TCCTCTC	CTCTTCTCCT	CCAAGCTTTT
Variegata	TTGCGATTTA	TGCTCCCTCC	TCTCCTCTCC	TC-TCCTCTC	CTCTTCTCCT	CCAAGCTTTT
Tsedey	TTGCGATTTA	TGCTCCCTCC	TCTCCTCTCC	TC-TCCTCTC	CTCTTCTCCT	CCAAGCTTTT
Magna	TTGCGATTTA	TGCTCCCTCC	TCTCCTCTCC	TC-TCCTCTC	CTCTTCTCCT	CCAAGCTTTT
Gommadie	TTGCGATTTA	TGCTCCCTCC	TCTCCTCTCC	TC-TCCTCTC	CTCTTCTCCT	CCAAGCTTTT
Kay_Murri	TTGCGATTTA	TGCTCCCTCC	TCTCCTCTCC	TC-TCCTCTC	CTCTTCTCCT	CCAAGCTTTT
Ada	TTGCGATTTA	TGCTCCC---	--TCCTCTCC	TC-TCCTCTC	CTCTTCTCCT	CCAAGCTTTT
Balami	TTGCGATTTA	TGCTCCC---	--TCCTCTCC	TC-TCCTCTC	CTCTTCTCCT	CCAAGCTTTT
Tullu_Nasy	TTGCGATTTA	TGCTCCC---	--TCCTCTCC	TC-TCCTCTC	CTCTTCTCCT	CCAAGCTTTT
Alba	TTGCGATTTA	TGCTCCC---	--TCCTCTCC	TC-TCCTCTC	CTCTTCTCCT	CCAAGCTTTT
Dabbi	TTGCGATTTA	TGCTCCC---	--TCCTCTCC	TC-TCCTCTC	CTCTTCTCCT	CCAAGCTTTT
Enatite	TTGCGATTTA	TGCTCCC---	--TCCTCTCC	TC-TCCTCTC	CTCTTCTCCT	CCAAGCTTTT
Gea_Lamie	TTGCGATTTA	TGCTCCC---	--TCCTCTCC	TC-TCCTCTC	CTCTTCTCCT	CCAAGCTTTT
Manyi	TTGCGATTTA	TGCTCCC---	--TCCTCTCC	TC-TCCTCTC	CTCTTCTCCT	CCAAGCTTTT
Red_Dabi	TTGCGATTTA	TGCTCCC---	--TCCTCTCC	TC-TCCTCTC	CTCTTCTCCT	CCAAGCTTTT
Dukem	TTGCGATTTA	TGCTCCC---	--TCCTCTCC	TC-TCCTCTC	CTCTTCTCCT	CCAAGCTTTT
Quncho	TTGCGATTTA	TGCTCCC---	--TCCTCTCC	TC-TCCTCTC	CTCTTCTCCT	CCAAGCTTTT
Karadebi	TTGCGATTTA	TGCTCCC---	--TCCTCTCC	TC-TCCTCTC	CTCTTCTCCT	CCAAGCTTTT
E.pilosa A	TTGCGATTTA	TGCTCCC---	-------TCC	TC-TCCTCTC	CTCTTCTACC	CCAAGCTTTT
E.pilosa B	TTGCGATTTA	TGCTCCC---	-------TCC	CC-TCCGCTC	CTCTTCTCCC	CCAAGCTTTT
E.minor	TTGCGATTTA	TGCTCCC---	-------TCC	TCATCCTCTC	CTCTTCTACC	CCAAGCTTTT
E.curvula	TTGCGATTTA	TGCTCCC---	----------	---TCCTCTC	CTCTTCTCCC	CCAAGCTTTT
E.trichodes	TTGCGATTTA	TGCTCCC---	----------	---TCCTCTC	CTCTTCTCCC	CCAAGCTTTT
scaffold2788_17.4	GGCCTCTCCC	CCTTTTCTGC	TTCGAATTGC	--TTT-TTTT	TTTTCTCGTG	CTCTCACTGC
Addisie	GGCCTCTCCC	CCTTTTCTGC	TTCGAATTGC	--TTT-TTTT	TTTTCTCGTG	CTCTCACTGC
Beten	GGCCTCTCCC	CCTTTTCTGC	TTCGAATTGC	--TTT-TTTT	TTTTCTCGTG	CTCTCACTGC
Rosea	GGCCTCTCCC	CCTTTTCTGC	TTCGAATTGC	--TTT-TTTT	TTTTCTCGTG	CTCTCACTGC
Variegata	GGCCTCTCCC	CCTTTTCTGC	TTCGAATTGC	--TTT-TTTT	TTTTCTCGTG	CTCTCACTGC
Tsedey	GGCCTCTCCC	CCTTTTCTGC	TTCGAATTGC	--TTT-TTTT	TTTTCTCGTG	CTCTCACTGC
Magna	GGCCTCTCCC	CCTTTTCTGC	TTCGAATTGC	--TTT-TTTT	TTTTCTCGTG	CTCTCACTGC
Gommadie	GGCCTCTCCC	CCTTTTCTGC	TTCGAATTGC	--TTT-TTTT	TTTTCTCGTG	CTCTCTCTGC
Kay_Murri	GGCCTCTCCC	CCTTTTCTGC	TTCGAATTGC	--TTT-TTTT	TTTTCTCGGG	CTCTCTCTGC
Ada	GGCCTCTCCC	CCTTTTCTGC	TTCGAATTGC	--TTT-TTTT	TTTTCTCGTG	CTCTCACTGC
Balami	GGCCTCTCCC	CCTTTTCTGC	TTCGAATTGC	--TTT-TTTT	TTTTCTCGTG	CTCTCACTGC
Tullu_Nasy	GGCCTCTCCC	CCTTTTCTGC	TTCGAATTGC	--TTT-TTTT	TTTTCTCGTG	CTCTCACTGC
Alba	GGCCTCTCCC	CCTTTTCTGC	TTCGAATTGC	----T-TTTT	TTTTCTCGTG	CTCTCACTGC
Dabbi	GGCCTCTCCC	CCTTTTCTGC	TTCGAATTGC	----T-TTTT	TTTTCTCGTG	CTCTCACTGC
Enatite	GGCCTCTCCC	CCTTTTCTGC	TTCGAATTGC	----T-TTTT	TTTTCTCGTG	CTCTCACTGC
Gea_Lamie	GGCCTCTCCC	CCTTTTCTGC	TTCGAATTGC	----T-TTTT	TTTTCTCGTG	CTCTCACTGC
Manyi	GGCCTCTCCC	CCTTTTCTGC	TTCGAATTGC	----T-TTTT	TTTTCTCGTG	CTCTCACTGC
Red_Dabi	GGCCTCTCCC	CCTTTTCTGC	TTCGAATTGC	----T-TTTT	TTTTCTCGTG	CTCTCACTGC
Dukem	GGCCTCTCCC	CCTTTTCTGC	TTCGAATTGC	----T-TTTT	TTTTCTCGTG	CTCTCACTGC
Quncho	GGCCTCTCCC	CCTTTTCTGC	TTCGAATTGC	----T-TTTT	TTTTCTCGTG	CTCTCACTGC
Karadebi	GGCCTCTCCC	CCTTTTCTGC	TTCGAATTGC	----T-TTTT	TTTTCTCGTG	CTCTCTCTGC
E.pilosa A	GGCCTCTCCC	CCTTTTCTGC	TTCGAATTGC	CTTTTCTTTT	TTTTCTCGTG	CTCTCGCTGC
E.pilosa B	GGCCTCTCCC	CCTTTTCTGC	TTCGAATTGC	TTTTT-TTTT	TTTTCTCGTG	CTCTCGCTGC
E.minor	GGCCTCTCCC	CCTTTTCTGC	TTCGAATTGC	CTTTC-TTTT	TTTTCTCGTG	CTCTCGCTGC
E.curvula	GGCCTCTCCC	CCTTTTCTGC	TTCGAATTGC	----T-TTTT	TTTTCTCGTG	CTCTCGCTGC
E.Trichodes	GGCCTCTCCC	CCTTTTCTGC	TTCGAATTGC	----T-TTTT	TTTTCTCGTG	TTCTCGCTGC

```
scaffold2788_17.4      TGGCTACTTT GGTCGTTTGA TCTGGGCTAC
Addisie                TGGCTACTTT GGTCGTTTGA TCTGGGCTAC
Beten                  TGGCTACTTT GGTCGTTTGA TCTGGGCTAC
Rosea                  TGGCTACTTT GGTCGTTTGA TCTGGGCTAC
Variegata              TGGCTACTTT GGTCGTTTGA TCTGGGCTAC
Tsedey                 TGGCTACTTT GGTCGTTTGA TCTGGGCTAC
Magna                  TGGCTACTTT GGTCGTTTGA TCTGGGCTAC
Gommadie               TGGCTACTTT GGTCGTTTGA TCTGGGCTAC
Kay_Murri              TGGCTACTTT GGTCGTTTGA TCTGGGCTAC
Ada                    TGGCTACTTT GGTCGTTTGA TCTGGGCTAC
Balami                 TGGCTACTTT GGTCGTTTGA TCTGGGCTAC
Tullu_Nasy             TGGCTACTTT GGTCGTTTGA TCTGGGCTAC
Alba                   TGGCTACTTT GGTCGTTTGA TCTGGGCTAC
Dabbi                  TGGCTACTTT GGTCGTTTGA TCTGGGCTAC
Enatite                TGGCTACTTT GGTCGTTTGA TCTGGGCTAC
Gea_Lamie              TGGCTACTTT GGTCGTTTGA TCTGGGCTAC
Manyi                  TGGCTACTTT GGTCGTTTGA TCTGGGCTAC
Red_Dabi               TGGCTACTTT GGTCGTTTGA TCTGGGCTAC
Dukem                  TGGCTACTTT GGTCGTTTGA TCTGGGCTAC
Quncho                 TGGCTACTTT GGTCGTTTGA TCTGGGCTAC
Karadebi               TGGCTACTTT GGTCGTTTGA TCTGGGCTAC
E.pilosa_A             TGGCTAC-TT GGTCGTTTGA TCTGGGCTAC
E.pilosa_B             TGGCTAC-TT GGTCGTTTGA TCTGGGCTAC
E.minor                TGGCTAC-TT GGTCGTTTGA TCTGGGCTAC
E.curvula              TGGCTAC-TT GGTCGTTTGA TCTGGGCTAC
E.trichodes             TGGCTAC-TT GGTCGTTTGA TCTGGGCTAC
```

Supplementary Figure S6. ***Discovery of a novel SRR marker in 20 tef and four closely-related wild Eragrostis species***. The multiple sequence alignment shows several polymorphisms between tef cultivars and wild *Eragrostis* species. The corresponding part of scaffold2788 from the genomic sequence is also included for comparison. The alignment is variable at 32 sites, of which 25 sites are informative for parsimony. Variable positions are highlighted in yellow.

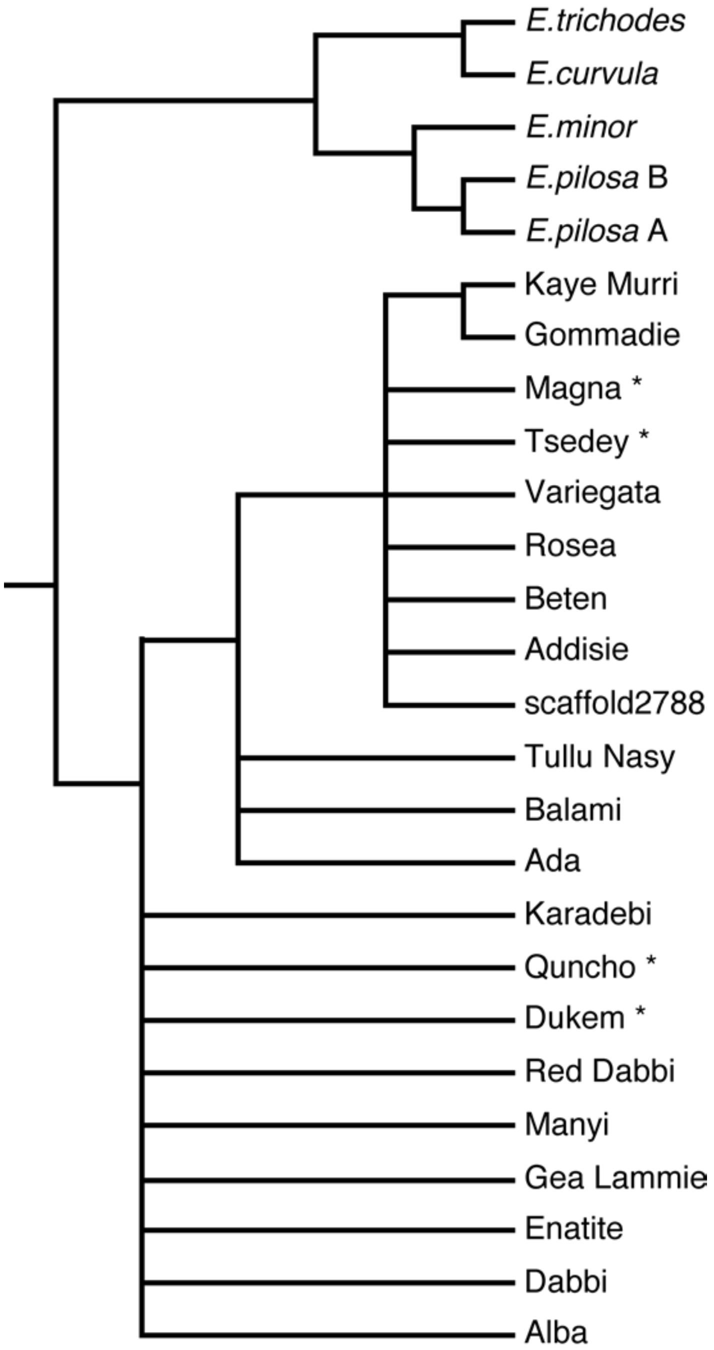

Supplementary Figure S7. ***Phylogenetic Tree of natural accessions and improved varieties of tef as well as wild Eragrostis species.*** One of four most parisomonious phylogenetic trees shows the relationship between the 20 tef cultivars and four closely related wild species. *E. pilosa* had two PCR amplicons of similar size which were sequenced and are labeled A and B. They could be the homeologs of the allotetraploid. The * represents improved varieties.

A

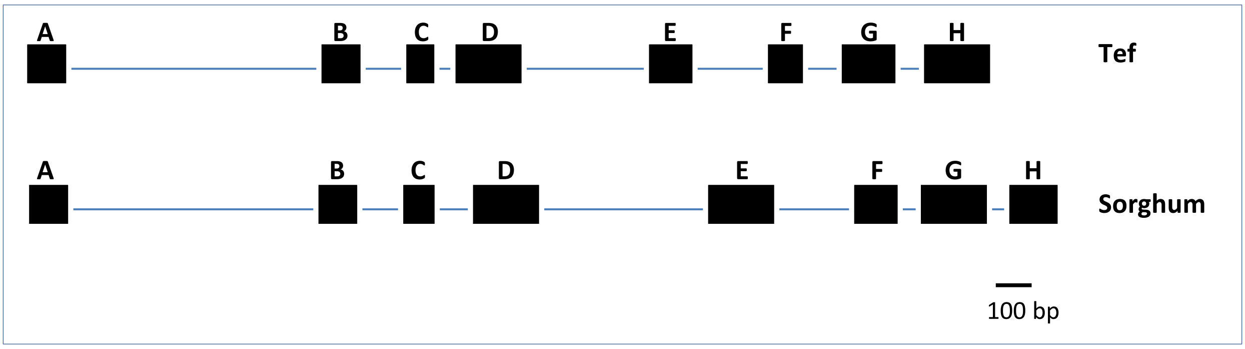

B

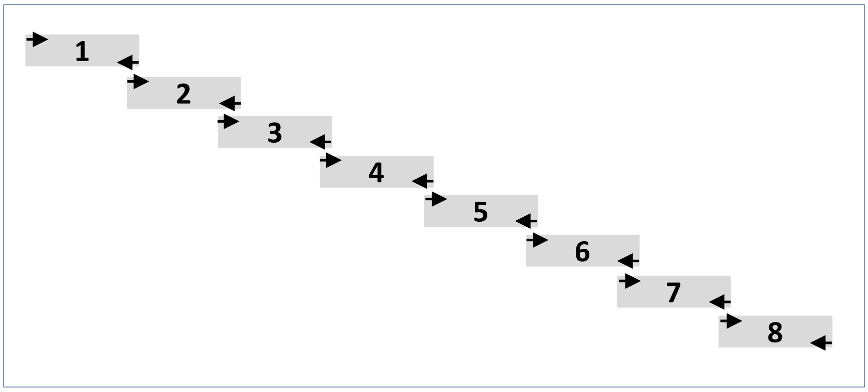

C

PCR products	Forward primer	Reverse primer (5'..3')	Expected amplicons (bp)
1	CTACATACTTCGAGTCCAGTC	CTTAGGTGACACCTGGCAGA	325
2	ACATGGCTGCGCGCTTGG	GATCCAATTTAGTGCAGTC	2144
3	GGAGTTGCTGTCCTCTCTAGT	GCAACTAAAAGGCTGTGCAGT	1718
4	TAAAGGCTGTCCAGGAAACTA	CAGCTTGGCGAGTGATGTAA	1822
5	AAATAAATCACAGCCGCACA	TGGAGTGCGATGCTTCATAG	2052
6	TCCGCTTTTCTGAGTCCATT	GCTTCATCTTCGTGTCGTGA	2058
7	TGCCAGGTTACTTGGTCTCC	TTTTCCACCATCAATCGCAG	1229
8	AATGATGGCGCTCAAGAAAT	TGAGATGAGGGGAACCATGT	1936

D

ccatactctctccatccttccattctttaatttccatgggtctcggcccattcctacggcccctatcattgcctcggccc 80
atttatgtaaactacaggcagggctcgtgatccgtttctcaccaattgggctcgttcatcctctttctttctcgcccaca 160
ccgctacatacttcgagtccagctcagctgcacgcacacctgacatggctgcgcgcttgggcctcctcccgcacgccgcg 240
ctccgcctcgcctccgcctcgtcctcctcctcccgagctgtcgccccgtctcgcgctcgcctcccttatcctcctctgcc 320
cttccccacctaccgccgcgccgcctccgcagctccgccgcacctccattcccgccgctcacacctcgcgccgcgcgcc**A 400**
TGTCGTCGTCGTCAGGGAACCCATACGCCGCGGAGCTCGCCGCCGCCAAGAAGGCCGTCACCCTCGCCGCAAGCCTCTGC 480
CAGgtgtcacctaaggccctaacaccctccctcgctccttagctcgcttctgctccctccccacctcgtgctgtgcggcc 560
ctcactgccgctggaagtgctgagctgttcttgtttttttttcctctctccctaccttccactgtcagtgctcactgct 640
caggagttgaggagtagctctgggggttcaccattaaccgctgctttgggttttaaattgcactcagtgataagataaat 720
ttgccttcctgcttcagtctgaatgaatgcttgtctcatgtgcgcgagttatggagaggaaacgagcgccagagggtaac 800
atagtgttcgttatgtcagcataatactttttttagggatatcaactgttgcaacgcgctcctggtttattggtctccaaa 880
tttaatgctggtttaggatagtcttggctatgataaccgctttaccaccactgttggagcactgaatttggaggtggaac 960
caattcgagtcggataaatgaatagcatcgtttagctgcattttggatttatttttattgttctgtgtttttgttcctcc 1040
tcag**ACTGTGCAACAAGACATTGTGGAGTCTGACGTTCAGTCTAAGCCGGACAAAAGTCCTGTCACAGTGGCCGATTATG** 1120
gtaagtgcttatcctgcaaatgtacgtctgcaagtggctgcatttcttcttggtactgaattgttgatgctagtgctaa 1200
aacaggaacttgaagtcagctgagtaacttttttatagaaatgcgcatctgagtaaaatttctaaatttttgtggagagtt 1280
tattgcattgggattagctaggggtgaaaacaacattagggatgatatgctgttctgttcatcagtcattattaatgtat 1360
gaaacttgttctttttcccaacttactgtttacctgttcacacattcag**GATCTCAAATATTGGTGAGTCTCATCTTGAA 1440**
GATGGAAGCACCAGGCTCATTTTCATTGGTGGCTGAGGAggtacttattacccttttttattcacttgtttacatatagat 1520
ttcatgagtgcttttgtaattaactggcaccagatgcgtgtgtag**GATTCGGAAGAATTGAGAAAAGATGGTGCTGAAGA 1600**
AATATTGGAAAATGTTAGAGTTAGGGTACTGGACGACTTGATTCCTCCAACCCTAGATGGGTGGGTATATCTATATCCAA 1680
CATACATGGGCCTCTATATGGGCCAACACACTCCAACACCCCCCCGCAGTCTGACCAGCCGGCCATGGCGGCGGCGGCGG 1760
CTAGGGTTAGGGTTGGGTAGGGAGAGAGAGAGGCTCTGATACCATGTTAGAGTTAGGGTACTGGACGACTTGATTCCTCC 1840
AACCCTAGATGGGTGGGTATATCTATATCCAACATACATGGGCCTCTATATGGGCCAACACACTCCAAAGAAAATATTAC 1920
CGATCTTGTGAATGAAACTATCTTTGATGATGGTTCATATAACATTTCATTTTCTAAGGATGGTATCCTCTCTGCGATTG 2000
ATGATGGAAAGTCCAAGGGAGGTCCATCCGGGCGACATTGGGTGCTTGATCCGATCGATGGGACTAAAGGgtcagtttaa 2080
taacttaaagtgacaggttttagctccttgttctataaatgacagattattaagttagtattgcttcttgctttcgtatg 2160
ttttattatatcataaaggtacagataccaaacatttttttttgttccaaagaatggagttgctgtcctctctagtgta 2240
tgaatactagaatgaattcatttagtgttatgtattcctttatcattgacgtttaacaatcatttctctcttgttaggtt 2320
tagtcgagactgcactaaattggatcgagaaattactatgacatagtcatgaactggatacaacatgctaaatgacatgt 2400
agttcaacaagtttagagaagagcaacccgcattttttttaaaaaaaaagagagaagtttagagcagagtggcagacca 2480
tgtgtgattcagtttccaaatcacaagtgttaaccgatcatattggttgttaccttactgcattgaatattgtactttgc 2560
ttctttcctataccatattctaagttctaacttagcattgtaatatgatcagtcatgaagtcatacacacgtgtcagcag 2640
tttatactgtctaaaaaaggattgttttcagacattatttctttctttttattggtaatataattctgtaccgcctttt 2720
gctatcactatcttgttaccatgctaataaatttcctttcctcatccaaagtttctttttgtaacttgaatcag**TTTCTT 2800**
AAGGGGTGACCAATATGCAATTGCGCTGGCTTTGCTTGATGAAGGTAAAGTTGTTTTGGGTGTATTGGCTTGTCCAAATC 2880
TTCCTTTGTCATCAATAAGCAACCTCAATGGTAGCTCTTTGGGAGATCAAGTCGGTGCCCTGTTTTCTGCTACAATTGGT 2960
TGTGGGGCAGAAGTAGAGTCCCTATATGGCTCTCCGCCGCAGAAGgtacatttattaattggtgtgatgatttaaaccca 3040
atctgaattataattcttgtttagcttttgatcttactgaatttcgaattttgatatatttttaacttctatctgtaatt 3120
tcag**ATTAGTGTTTGTTCCATTGGCAATCCAGTTGACGCTTCTTTCTTCGAATCCTATGAAAGTGCTCACTCCTTGCACG 3200**
ATCTGACTAGCTCTATTGCAAAGgttggtcccaagctattctttgcttagtccaggtctatgggagttagtgaagtaatc 3280
agtggttcattggtatcag**AAACTTGGAGTCCAAGCTCCTCCTGTTAGAATTGACAGCCAAGCAAAATATGGTGCTCTGG 3360**
CCAGAGGCGATGGTGCTATTTACTTGCGCTTTCCGCACAAAGGTTACAGAGAGAAGATATGGGACCATGCAGCCGGCTCA 3440
ATTGTTGTCACAGgtatgaagccctcccatatgtttttcccctcataaatatgattcaaagagttatatttatttacatt 3520
tcctatctcttatgtttttgcag**AAGCTGGAGGTATAGTAACAGATGCCTCAGGAAAAGACTTGGATTTCTCCAAAGGAA 3600**
GGTTTCTTGACCTTGACACTGGCATCATTGCAACAAACAAGCAGTTGATGCCATCGCTCCTAAAGGCTGTCCAGGAAACT 3680
CAGAAGCTGGAGGTATAGtaacagatgcctcaggaaaagacttggatttctccaaaggaaggtttcttgaccttgacact 3760
ggcatcattgcaacaaacaagcagttgatgccatcactcctaaaggctgtccaggaaactattaaggagcaaaaccaggc 3840
tgcttccctcttgtagcagatatagcgactagcgatcaatgaaatgtttgttgcaatactgtttcatatattgtactgca 3920
cagccttttagttgcattacactatcatgttgtactatgtttataaaatctacctagtggctgatcaggcaccattcttg 4000
tttatctttaatagtggtagaagtttccgtttgcttctgtatacacttctgaataatgtaacttggttaaaaaaacattt 4080
agaaaattgtgaatctgcacgcatcagtatagttgacttggtatgactattcattttgaaccatggccaactttattat 4160
gatattctgcggactgcttgcagtttatcaccagtaattatccagcacctcttatcttcaaacatctgaactgaactcca 4240
ctgagttcagatagcctgctgcatctggaggtttgcttttgtggccaacatctttcaacatttaatcacgaagtgtttac 4320
atgttatacaagaaatgcttaccatacaacactcattgattcctcaatactaccggcatatcaagaatatgagcatgctc 4400
gttaacaggacagggcaccaacataaaaggttgaccatcatatcctccgcgcgacacaagtagcaggcattatggtcaag 4480
caatgatctgatagagaatgttgacactggcatttacatgataaacatgtacagaaaatcaaagtagcatcgattactag 4560
ggaaaagatgcaaagtgtaatcaactatgggaaggtttgccttttctgagtcaaatttttatgcagtagctcattgcat 4640

Sal1:
copy 1

attgcttctttttatcatgttatataatatttcttatgtttggatgtccatgagaaagaaatagaaaatttatgcaattt 4720
aaaatttgcaaacagatataaaacggagcgaatgtgaaccacacccgccacctccattggcgcctgccatgtgaacggct 4800
gcgacggagcgaattggcatcgcagatggcacctcgaggcagcaggttcatcgaggaagcaaattaaagttttattgtac 4880
cagatgcatggctgctcctaggcggcggccaattcaggctcatgcgacaaagctaggtgtaggtggaccggacggggaag 4960
gaggagcggatgtgcgccacggcggcgaactgcaggcgcggaccgcgcggtgaccatgtgaatccgtcaagtccgagcat 5040
ttcgttcgtgaggggaagcagagcggaaagaccgcagcatgcagttaccaaagcacccttagttacaattgattaatggt 5120
tgggattaattcaccggggaggaacctctagttcccctcaagcaccgtagcaaatctcatatctgcatacctccaaccct 5200
agaattctttggtatggatcagattcattcaaaataggggaagaagaaaccatgttccaaataggggaagaagaaaccaa 5280
atagaaaataaatcacagccgcacatcacaaccaccgcgaggtccacgagaccaagagccgcacatcacaactaccgcga 5360
ggtccacgagaccacgaggtccacgacggaccaagccgaggtcttgttgaaaaacagaggccgccgctgccgcccactct 5440
gccctgagttgagttcccctgttttacatcactcgccaagctgccactccgtccccgtccccgtccccagcc**ATGTCTCA 5520**
GCCATCAGAGGTCGCCGGCAACCCGTACGGGGCCGAGCTCGCGGCCGCCAAGAAGGCCGTCGCCCTCGCTGCCCGCCTTT 5600
GCCAGgtttcctcacagatccctctctgttttccgccatgcccttccatatgggatccggtttggagttggatggtatat 5680
ttggtggagtcctacagctgatcacaccaagaagatgtaggaaaattttaggctccgggtggcatgtagtctttctgttt 5760
ctgacttgatgtagctttgcattgaggaaaagaaggcagagccggtagtcggtagatatccctctaggcctgtctttctt 5840
caatgtgcttgattagtagaaaatgcacagatgtttatgatgccctgtaggccattacattgaggtttctgatagtaagc 5920
tcattgaaaatccatcttggtagataaacatctgatgaaacagttcactgtagttattttctggataattattggattct 6000
tgcttatgtcactgcattgcctgattctttccgttctttgtttcattgtgttttcag**AGGGTGCAGCGGGACATCTTGCA 6080**
GTCCGACGTCCAATCAAAGGCTGACAGAACTCCTGTGACAGTTGCGGATTATGgtacctgcgtatactggagtttctgta 6160
atttatgaaataattaacttggatatcatgatcatcatgtgttatccttcagttctagttaacataatatggattcgttt 6240
caaacatagaatgaaacatgcaggactcttgcttattatgtacttttgacttaattgatacttttgggtttctgaccag**G 6320**
ATCTCAAGTATTGGTAAGTTTGGTCCTGAAGATGGAATTACCTTCTCACTCTTTTTCTATGGTGGCTGAAGAGgtgaccc 6400
gtcctttgtttcgttactgtaattgatcaccgaagtactttcctgtaactaatttgtgacatgttctcgcag**GATTCAAA 6480**
AGACTTGAGAAACGATGGAGCTCAAGAAATTCTAGAACACATCACTAATCTTGTGAATGAAACCGTCGTAAGTGATGGTT 6560
CATACAAAGTTTCGTTATCTAAGGATGATGTACTCACTGTGATTGATGGTGGGAAATCGGAGGGAGGTCCATCTGGGCGA 6640
CATTGGATATTGGATCCAATAGATGGAACTAAAGGgtgagtttgataactcattgtttcattttcgaccacagattatag 6720
cttatccagatgtactcagggcgaacaaaagtagatttatcaaattagactggcatactcgagtgacattcttttatgac 6800
caatgaatagatcatctgatttgctagatcttatttcactcaaaaagtaaaaactaacacagttggaaatcagaatatta 6880
ggtaagcgtggataagaactcaaaaaagctataatgcagctatcagttcgaaattcttattgggaaaacttgatgcag**TT 6960**
TCATAAGGGGAGACCAATATGCAGTTGCACTTGGGTTACTTGATGAGGGCAAAGTTGTTTTGGGTGTGTTGGGATGTCCA 7040
AATCTTCCATTAAAATCATCAAGAAAAAACAATGGTGGCTCTTCTGGGGATGTAGTTGGCTCCCTTTTTTCTGCTACAAT 7120
TGGTTGTGGAGCTGAAGTTGAGGCCTTAGAGGGATCTAATCCAGAAAAGgtaataaatgttttcatttgattcatgaatc 7200
atatccacttccgcttttctgagtccatttctttcttatttgctcgtttttttaactcacattctag**ATTAGTGTTTGC 7280**
TCTATCAACAATCCAGTCGATGCCTCATTTTTTGAATCCTATGAAGCATCGCACTCCAAGCGTGATTTGACTAGTTCCAT 7360
CGCTGAggttggtcctttgcaatgatttctttgtctagaggtcttatgagatatatttttgaatcctttatttttatgt 7440
tattaga**AACTTGGCGTCGAAGCTCCTCCAGTTAGAATGGATAGCCAGGCCAAGTATGGTGCTCTAGCTCGTGGCGATGG 7520**
TGCCATTTTTCTCCGCATTCCACATAAAGGTTACAAAGAAACGGTTTGGGATCATGCAGCAGGGTCAATTGTTGTCACAG 7600
Gtacatagttctacaaagagatacttggttgaacaactaaatgatcctttttctaactgcacccatttctgataatttgc 7680
ag**AAGCTGGTGGCATGGTAAAAGATGCATCAGGAAATGATTTGGATTTCTCCAAAGGCAGACATCTTGATCGTGACAGAG 7760**
GCATTATTGCAACAAATAAATATTTGATGCCTCTAGTTTTGAAAGCAGTCCAAGAGGCTATTAAAGAGGAACAGCAGGCT 7840
GCTGCGCTCGTGTAGcagtccatatattggcaccaaaatctccaatcggaataccagtttacacaacattgtgtacaaac 7920

Sal1: copy 2

aaagtacaacttatcattgtaccatacactgtaataacttgtgatttgtgatccattgacggcactgtggatgcaaatat 8000
agccagtctgcctcagcctatgtttctgcattgtttatcctttttttagcaatcttaggccaaattgcatgagtgatttca 8080
ttattatggttcaataaatataagagtgagtccagtgccaactggtctgatagataaatgattctgagtattctattttt 8160
aataaatatagggaacaatatgtttggtacatatatgaggaaaaaaatagcatcagtgacagaagttccagagactgggg 8240
ctgagttctgttgtgttctgctaaatatcaatgtgtatttaatatgatgctatattgagatttagattaatttttttgga 8320
tattggaccacgaaagatgaaatccaggtatttgacctttcatgcctacttttcatttctacacggtttgtggtttgtt 8400
ttttgagtcaaaattttcatgttgtagtagctatttgcatgtagtttcttcgttttggtttcactaatttagcacatgtt 8480
agagtaagtatgccgctaattgagtacaaaaaagaaggatattacgacattttaatggtttttatgttagggcaattttg 8560
gtgaaaactttctcccacagccacaaaaagcaagtctggaggtgctgtgaggaagaactttggagaaagtaggggccttt 8640
ggttggctttggactttttaaaaagcaaaagcactttgaaaagaagaaccaaacacaccccagtatgtccatgggaaaga 8720
aatagaagttttatgcgattgaaaattagcaaatataaaaaatccctcactaagaaaatctggcccggaaacaatatctg 8800
cgtacctcgagccctagctatttttttttcgagaatcgagccctacctttcttaggtattgccggtatatatgtacattca 8880
gtcacatcacaagttcacaaccactaccgcaactaaccaaagcctagttgttattgcaaaaaacagaggccgccgccaac 8960
tgtctttgagttccctcccctgtttgacttcactcgccaagcccactccctctgtcatagtccccgtaccccgcc**ATGTC 9040**
GCAGCCTCCGGAGGTCGCCGGCAACCCGTACGGGGCCGAGCTCGCGGCCGCCAAGAAGGCCGTCTCCCTCGCTGCCCGCC 9120
TCTGCCAGgttacttggtctccccacagatgttttatcgttatctgttttccgcccttcccttccttccagggtccgttc 9200
tagactctggagttggatttggtacatttgctgtagtcctaggacagatcacgacacgaagatgaagctaaatttcaagc 9280
cagatggcatgtttttttttcttgacttgatggaactttgcattgaggaaaagaaggtagaaccggaagatatccctcc 9360
aggactgtcttttattttgagacaatagcgttttaaccatgattctacactctaattgcgttttcaccatcgattttca 9440

Sal1: copy 3

gattttacaagatcaccattgattttgaaaactgatggtaaaatcgcaaaacaattttgcaagatcaccatcgcttttc 9520
gaaaaatctgaaaaccaatggtgaaaacacaattagagtttgaaaggatggatgttttgcagttgcccctttattttttt 9600
atgtgtttgattggtagaagatgcacagatttttatgatgccctgtaggctattgcatgggattctcatgttaggctgat 9680
tgagaatccatcttggtagataaacatctgatgaaacagttcactgtggtattatctgaataattgttttggattggatt 9760
ggattcctgcatagcgtcactgcgctaacctgattctttatgatctttgttacgctctgtttttcag**AGGGTGCAGCGGG 9840**
ACATCTCGCAGTCCGATGTTCAATTAAAGAGAAACAGAACTCCTGTGACAGTGGCGGAttatggtacataagcatactgg 9920
agttgctgtaatttataaaacaaataacatggatatcatgataatcatgtgttctccttcatctctagttagcatagtat 10000
ggatgcctgtcaaaagtaaaatgaatcatgcataaatctttcttattatgtacttttgattctgtgaacag**GATCTCAAG 10080**
TATTGGTAAGTCTGGTCCTGAAGTTGGAATTACCTTCTCACTCCTTTTCCTTGATGGCTGAAGAGgtaccccgtcctttg 10160
ttccgttgatgtattagattgtcaaagcactttcctgtaattaatttgtcaaatgtactctcgcag**GATTCAGAAGACTT 10240**
GAGAAATGATGGCGCTCAAGAAATTCTGGATCACATCACTAATCTTGTGAATGAAACCATCGTAAGTGATAATTCATACA 10320
AAGTTTTGTTATCTAAGGACGATGTACTCGCTGCGATTGATGGTGGAAAATCCAAGGGAGGTCCATCTGGACGACATTGG 10400
ATATTGGATCCAGTAGATGGAACTAAAGGgtgagttagaaaattctccatttcattttcgtccacaaataatagcttgtc 10480
tcacagtcagtctcacagatgtactcaggatgaactaaaatagatttaccaaattagactggcatactcgagtgacgtcc 10560
tttatgaccaatgtataactcatatctgatttgctagatcttatgtactctaagttaaaattaacacggttgtaagctga 10640
gtagaatattagttaagcatggatgagtaagaacctaaaacagctataatgcggctatcagctcgaaatttttattgtga 10720
agacttgatgcag**CTTCATAAGGGGGGACCAATATGCAGTTGTACTTGGGTTACTTGATGAGGGAAAAGTTGTTTTGGGT 10800**
GTATTGGGATGTCCAAATCTTCCGTTAAAATCATCAAGCAAACACAATGGTGGCCCTTCTGGAGATCTAGTTGGCTCCCT 10880
CTTTTCTGCTACGATTGGTTGTGGAGCTGAAGTTGAGGCCTTAGGGGGGTCTAACCCAGAAAAGgtaacaaatatttttc 10960
atttgattcaaaatcatacccactttcacttctctggttccatttctttcttatttgctcagtttttctaacccacttg 11040
cattctag**ATTAGTGTCTGCACTATCAGCAATCCAATCGACGCCTCATTTTTTGAATCCTATGAAGCATCACACTCCAAA 11120**
CGTGATTTGACTAGCTCCATAGCGGAGgttggtccttggaaattatttcctttttctagagatcttatgaagtatattga 11200
taaaccctctattttatgtaattag**AAACTTGGTGTCCACGCTCCTCCTATTAGAATGGATAGCCAGGCCAAGTATGGTG 11280**
CTTTAGCACGTGGTGATGGTGCCATTTTTCTGCGCATTCCACATAAGGGTTACAGAGAAACGGTTTGGGATAATGCGGCG 11360
GGGACAATTGTTGTCACAGgtacatagttctacaaagcagataattaggtgaactactaaatgatcctttttctaactgc 11440
gccccattcctgatatttgcag**AAGCTGGTGGCATGGTAAAAGACGCATCAGGAAATGATTTGGATTTCTCCAAAGGCAG 11520**
ACGTCTTGATCGTGACAGAGGAATTATTGCAACAAATAAATATTTAATGCCACTAGTTTTGAAAGCAGTCCAAGAGACTA 11600
TTAAAGAGGAACAGCAAGCTGCTTCGCTCATGTAGcagtaacgtattggcaccaaaatatccaatcatgataccagttta 11680

Sal1: copy 3

cacaacctcatgtacaaataaagaacaacttctcaatttactatatacactgttataactagtgagttgtgagtcatgtg 11760
acgccacatgtcatttgtctgaataaatggatgcaaatatatccaacctgcctcagcctatctatctgttgtttagcttc 11840
ctatagccatcttagtctgaattggctcagaaaaataatttagtgagtccgtccagtgccatgcaactggtctgatggat 11920
aaatgattctgattgttctatcattaaaaaatatagagaacaacatatttggtacatatttgaggaacaaaatagcatca 12000
gtgacagaagtgtcatagattggggttgagttctggtgttctgtttaatatcagtttatttagtacgatgcaatgttgag 12080
ttgtaggttatttttttgtggatattggcccacaaaagatgaaatccaggtatctgaaactttcgtgcctagtttttattt 12160
tctactcggtacatggttcccctcatctcagcacatctctaagttctcatctcttcactcatttcgcaaaagtatatttc 12240
tcttttcaaaccaagtcttgaatcttgtgattagtttctagactttagacactaaattctggattgatat

Supplementary Figure S8. ***Tandem duplication of SAL1 gene confirmed by Sanger sequencing.*** A) Structure of the SAL1 gene in tef and sorghum. B) Eight primer sets were designed to do PCR amplification and Sanger sequencing of the region on scaffold6855 where tandem duplications of the SAL1 gene was found. C) Sequences of primers used to amplify the region of the SAL1 tandem triplication on scaffold6855. D) The piece of scaffold6855 containing the tandem triplication. Sanger sequencing confirmed the genomic sequence containing three SAL1 genes in a tandem arrangement. Despite repeated attempts, one region (exons 2-4 of the first copy in blue) was not confirmed due to failure of the PCR amplification. Exons 1 and 5-8 were found. Exons are shown in uppercase and bolded.

Supplementary Tables

Supplementary Table S1. ***Summary of genome sequencing data for the tef genome***.

**Sequencing platform**	**Provider**	**Type of sequence**	**Insert size (bp)**	**Amount of sequence (Mbp)**	**Fold coverage of tef genome**
Roche 454-FLX	Macrogen, Korea	Mate-pair	3000	504	
	Functional Genomics Center Zurich, Switzerland	Single		1651	
		Mate-pair	3000	332	
			13000	450	
			6500	2343	
	Subtotal for 454-FLX			5282	7
Illumina HiSeq2000	Fasteris, Switzerland	Paired-end (2x100bp)	300	17004	
		Mate-pair (2x50bp)	3000	3452	
				9900	
		Single read (50bp)		693	
		Single read (100bp)		3472	
	Subtotal for Illumina			34521	44
Total					51

Supplementary Table S2. ***Summary of sequencing data for the tef transcriptome*.**

**Treatments**	**Technology**	**Provider**	**Replication**	**Library**	**Length after trimming (bp)**	**Number of reads**	**Number of bases after trimming (bp)**
Normalized Library	454-FLX	MWG Germany			329	1 065 255	**351108762**
Normal watering	Illumina HighSeq 2000	Fasteris Geneva	1	GNY1	70	6817359	477215120
			2	GNY1	90	18854229	1696880610
			3	GNY10	90	35853151	3226783590
			**Subtotal**			**61524739**	**5400879330**
Drought			1	GNY2	70	9381762	656723340
			2	GNY2	90	22252802	2002752180
			3	GNY11	90	40710706	3663963540
			**Subtotal**			**72345270**	**6323439060**
Waterlogging			1	GNY3	70	9413900	658973000
			2	GNY3	90	23625313	2126278170
			3	GNY12	90	37 852 175	3406695750
			**Subtotal**			**70891388**	**6191946920**
Total						205826652	18267374072

Supplementary Table S3. ***Summary of assembly statistics for the tef transcriptome***.

**Genome statistics**	**Tef assemblies**				**Sorghum**
	**Library**	**454Isotigs**	**Extended**	**Maker Predicted Genes**	
Assembly type		**Newbler**	Oases/Velvet/ Trinity		
Number of Bases (Mb)		40.8	126.7	61.3	43.7
Number of transcripts		38333	88078	42052	29448
Number of clusters		27756	28113		
N50 (bp)		1314	1902	1843	1769
Maximum size (bp)		10525	19219	14922	14671
Mean size (bp)		1064	1439	1458	1489
Percentage of reads mapped to transcriptome	GNY10	69.0	96.3		
	GNY11	67.4	95.9		
	GNY12	69.8	96.4		

Supplementary Table S4. ***Percentage of genes and bases found in tef transcriptome and genome***. Transcripts were compared with blastn with e-value 1e-10. The number of genes with a homolog found and the percentage of query bases in genes with a homolog that were aligned are reported.

**Genome**	**Assembly**		**% of Genes and bases found in tef genome assemblies**		
			**454Isotigs**	**Extended**	**GNY98ter_41. Closed (genomic)**
Tef	454Istotigs	Genes	-	99.4	99.9
		Bases	-	99.1	99.6
	Extended	Genes	72.5	-	99.3
		Bases	23.4	-	96.6
	Maker	Genes	72.4	92.7	100
		Bases	49.2	85.6	99.6
Sorghum	Transcripts	Genes	58.9	60.6	91.8
		Bases	38.4	80.9	57.8

Supplementary Table S5. ***Summary of assembly statistics for tef and other genomes***.

Genome		Number of scaffolds	Number of bases including N's (Mbp)	Number of bases without N's (Mbp)	Percentage of total genome size with N (without N)	N50 (bp)	Reference
Tef	contigs	4277022	-	482.2	66	832	Current work
	scaffolds	405558	710.8	455.9	97 (62)	66083	
	closed	405558	672.8	561.2	92 (77)	84898	
	Scaffolds greater than 1000 bp	14057	619.8	496.3	84 (67)	97605	
	Scaffolds synmapped to sorghum 3 genes[1]	3165	396.3	327.6	54 (45)	131533	
	Scaffolds synmapped to sorghum 5 genes[1]	2468	345.9	281.3	38	138303	
Cacao (*Theobroma cacao*)			326		76[2]	473000	[25]
Cucumber (*Cucumis sativus*)			243		66[2]	226000	[26]
Date palm (*Phoenix dactylifera*)			381		58[2]	30500	[27]
Foxtail millet (*Setaria italica)*			423		83[2]	1000000	[18]
		597	402.4		79[2]	12300000	[28]
Watermelon (*Citrullus lanatus*)			353		83[2]	2378000	[29]

[1] These sets are a subset of scaffolds mapped via synteny (using Coge's SynMap function) to the sorghum genome.The subset is labeled with the number of genes (3 or 5) that must be successfully mapped to assign the scaffold to the subset.
[2]Genome size of cacao is 430 Mbp, cucumber is 367 Mbp, date palm is 658 Mbp, foxtail millet is 510 Mbp and watermelon is 425 Mbp.

Supplementary Table S6. ***Percentage of reads mapped to genomic scaffolds greater than 1000 bp in length***.

Name of sequencing run	Library type	Approximate insert size (bp)	Number of reads	Reads mapped (%)	
				**Single reads**	**Paired reads**
GNY7 A	Single	-	34733967	64.3	unpaired
GNY7 B	Single	-	13878241	74.5	unpaired
GNY8 A	Mate-pair	4000	68636522	80.2	8.10
GNY8 B	Mate-pair	4000	8802636	82.3	8.28
GNY9 A	Paired-end	300	198008846	89.8	75.15
GNY9 B	Paired-end	300	170047686	83.8	72.8

Supplementary Table S7. ***Location of SSR markers [1] in the tef genome***. The name, location and complete sequence of the 592 SSR markers is listed. This Table is provided as a supplementary file [*Supplementary_Material_4_TableS7*] (excel file)

Supplementary Table S8. ***Amplification of scaffolds between CNLT markers via Sanger sequencing***. Position and sequence of the primers used for the amplification are listed as well as the annealing temperature and the elongation time used for the PCR.

**Scaffold name**	**PCR amplicon**	**Primer name**	**Primer sequence (5'…3')**	**Scaffold Position**	**Annealing temperature (°C)**	**Elongation time (sec.)**
2429	A	CNTLs316_F	ACGAGCGAGCTATCAATGGT	124,655	55	240
		S2429_R1	CCATCAAAGGAAGAGGGTGA	128,684		
	B	S2429_F2	CGAACAGCTTGGACATAGCA	127,327	53	210
		S2429_R2	ACTGCACCAAAAATGGGAAG	130,643		
	C	S2429_F3	ACAAGAACACCCGAATCGAA	130,033	55	240
		CNTLs472_R	GGGCTTGGATGGTACAAACA	134,508		
8420	A	CNTLs77_F	GGTAGGCCTTCCATTCCTTG	74,323	55	210
		S8420_R1	TCACTGCACTAGATTGGATATGAA	77,978		
	B	S8420_F2	GAAAATGATGGTGCCAAATG	75,918	53	240
		S8420_R2	GACCTGCTGAGGAGGAACAG	78,064		
	C	S8420_F3	GTTGGCATAGATCGGCTTGT	77,944	55	165
		CNTLs322_R	TTTTTCCATCAATCCCGTTC	80,487		

Supplementary Table S9. ***Sequence comparison between two tef scaffolds and correspon-ding sequences from Sanger sequencer.***

PCR amplicon				Scaffold name	Alignment of scaffold with Sanger sequence		
Name	Forward primer	Reverse primer	Length (bp)		length of alignment (bp)	Non-gapped or N position	
						Number	Percent Identity
A	CNLT316	CNLT472	9707	2429	9707	8740	99.3
B	CNLT77	CNLT322	8175	8420	8369	5767	96.5

Supplementary Table S10. ***Comparison between Tef Sanger sequencing and Tef NGS sequences for genes of agronomically important traits identified from other organisms.*** The choice of name, A or B, is arbitrary. The origin of each homeolog is unknown.

Trait	Gene	Organism [Reference]	Tef Sequenced by Sanger		Tef NGS sequences compared to Sanger sequences			
			Size (bp)	Copy	Scaffold name	Aligned (%)	Nucleotide identity (bp)	Nucleotide identity (%)
Plant height	BRI1	Rice [30]	726	A	2444	100	699	96.3
	CYP90B2	Rice [31]	852	A	2176	62.0	508	59.6
			811	B	2176	80.3	647	79.8
	CYP724B1	Rice [31]	2099	A	587	95.6	1968	93.7
			2079	B	9182	98.7	2043	98.3
	CYP90D2	Rice [32]	204		4982	100	202	99.0
	GA20ox2	Rice [33]	1374	A	21728	66.7	908	66.1
			1349	B	2025	82.0	1097	81.3
	KO2	Rice [34]	1596	A	8186	100	1594	99.9
			1459	B	13666	100	1447	99.2
	Tua1	Finger millet [35]	2652	A	867	100	2614	98.6
			2612	B	868	98.0	2552	97.7
	Tua2	Finger millet [35]	1876	A	2744	99.7	1857	99.0
			1884	B	3288	100	1882	99.9
	HTD1	Rice [36]	3354	A	3190	89.6	2990	89.1
			1176	B	2740	99.4	1166	99.1
	RHT1	Wheat [37]	490		C7801583	58.4	285	58.2
Seed yield	CKX2	Rice [38]	306		231	100	304	99.3
Grain size	GW2	Rice [39]	1281	A	2262	100	1276	99.6
			1275	B	3560	100	1248	97.9
	SW5	Rice [40]	503		3316	84.1	393	78.1
Drought tolerance	ERA1	Arabidopsis [41]	1408	A	2224	99.3	1398	99.3
			1356	B	520	100	1337	98.6
	LEA3	Rice [42]	510		6095	94.3	478	93.7
Herbicide tolerance	ALS	Rice [43]	300	A	6774	100	299	99.7
**Total**			**33532**			**93.9**[1]	**31192**	**92.5**

[1]Weighted average

Supplementary Table S11. ***Primers used to isolate agronomically important genes in tef*.**

Traits of interest	Gene from other organism			Tef amplicon (partial clone)	
	**Gene name (accession number)**	**Organism**	**Reference**	**PCR primers (5'…3')**	**Size (bp)**
Plant height	BRI1 (NP_001044077)	Rice	[30]	F:GCTCTCCCTCTCCTTCAACC R:CAGCCTGGATCTCCTTGAAC	2363
	CYP90B2 (AB206579)	Rice	[31]	F:CTTCTTTCTCCCCTTCATCCTCCTTGC R:TCCATGCTCATTATGTTCTTCGCCATC	852
	CYP724B1 (AB158759)	Rice	[31]	F:GGTGTTTAAGTCCCATCTGT R:CTGAAGATACCGGAATAGTTG	2100
	GA20ox2 (AB077025)	Rice	[33]	F:CGAGGAGATGAAGGAGCTGT R:CCAGGTGAAGTCCGGGTA	1375
	KO2 (NM_001064444)	Rice	[34]	F:GACTATGGTGACTTCCACA R:TCGCCTTCCTTGAGCCTCCA	1459
	RHT1 (AJ242531)	Wheat	[37]	F:ATGGAAGCGCGAGTACCAAG R:ACCACCGGTAAGGAGATCG	490
	TUA1 (AF008120)	Finger millet	[35]	F:ACCATGAGGGAGTGCATCTCGAT R:AATTCCCCGCTTTGCTACTGGGT	2652
Seed yield	CKX2 (HQ018816)	Rice	[38]	F:TTGCCATCCATATCTATGAGTC R:ATTTTAGCAACCTCATGCCACT	306
Grain size	GW2 (EF447275)	Rice	[39]	F:TCATCGAAGCACAGTTGAGG R:CATGATGAGCTTCTGCTAGAGAA	1159
Drought tolerance	ERA1 (NM_123392.1)	Arabidopsis	[41]	F:ATCTCGGGCGAAACTTTCAT R:CCCTCAGTCCACCCTCCAGT	1408

Supplementary Table S12 ***Location of tef CNLT markers in the pseudo-chromosomes of tef ordered by linkage group***. A translocation between sorghum and tef can be seen between linkage group 3 and tef pseudo-chromosomes 3 and 9. This Table is provided as a supplementary file [*Supplementary_Material_5_TableS12*] (word file).

Supplementary Table S13. ***Divergence dates in selected grass species estimated from modal Ks values***. The molecular dating estimates were computed from dS values obtained from CoGe [6, 7, 19] and compared to the estimates generated from the rht1 and sd1 genes [44]. In CoGe, each analysis is assigned a unique URL so that the workflow can be later recalled. The complete URL for these analyses is http://genomeevolution.org followed by the directory name given in the table. For example, http://genomeevolution.org/r/8jtc is the unique URL for the tef vs. tef analysis.

**Genotypes compared**		**CoGe URL directory name**	**Number of homologs compared**	**Ks CoGe (substitutions/site)**	**Divergence date (MYA) (upper and lower 95% confidence interval)**	
**Species**	**Subfamily [clan]**				**Current work**	**Ref. [44]**
Zea mays vs Eragrostis tef	Panicoideae *vs* Chloridoideae	/r/8jge		0.50	38.4 (21 – 55)	36.47 (20.64 – 50.54)
Zea mays vs Setaria italica	Panicoideae [Andropogoneae *vs* Paniceae]	/r/8i6i		0.30	23.1	26.86 (13.70 – 38.71)
Zea mays vs Zea mays	Both Panicoideae [Andropogoneae]	/r/8oqm		0.15	11.5 (0.07 – 23.9)	
Zea mays vs Sorghum bicolor	Both Panicoideae [Andropogoneae]	/r/8i70		0.15	11.5	14.20 (6.57 – 22.04)
Eragrostis tef 1 vs Eragrostis tef 2	Both Chloridoideae	/r/8jtc	5460	0.05	3.8 (0.07 – 46.9)	6.38 (1.51 – 11.77)
Eragrostis tef vs Sorghum bicolor	Chloridoideae *vs* Panicoideae	/r/8i5x	20466	0.47	36.1 (19.8 – 46.9)	
Eragrostis tef vs Setaria italica	Chloridoideae *vs* Panicoideae	/r/8i6k	22627	0.43	33.1 (17.8 – 41.8)	
Eragrostis tef vs Oryza sativa japonica	Chloridoideae *vs* Bambusoid/Ehrhartoideae	/r/8i6m	15066	0.58	44.6 (23.9 – 53.4)	
Sorghum bicolor vs Setaria italica	Panicoideae [Andropogoneae *vs* Paniceae]			0.27	20.8	
Sorghum bicolor vs Oryza sativa japonica	Panicoideae *vs* Bambusoid/Ehrhartoideae	/r/8dwo		0.57	43.8	
Setaria italica vs Oryza sativa japonica	Panicoideae *vs* Bambusoid/Ehrhartoideae	/r/8i6j		0.55	42.3	

Supplementary Table S14. ***Percentage identity between aligned segments of tef A and B pseudo-chromosomes excluding N's and gaps***. The 10 pseudo-chromosomes of the tef genome were obtained by aligning tef scaffolds to the 10 chromosomes of sorghum.

Pseud-chromosome	**Length of aligned sequences**	**Number of gaps**	**Number of N's**	**Differences (bp)**	**Identity**	
					**Length (bp)**	**(%)**
chr01	43978092	34644789	369333	632213	8331757	93.0
chr02	29271836	24005475	205936	394902	4665523	92.2
chr03	34558096	25833263	364178	570247	7790408	93.2
chr04	26121030	19274356	276664	445661	6124349	93.2
chr05	8158758	7453746	30211	56401	618400	91.6
chr06	18988905	14327081	199922	314472	4147430	93.0
chr07	14474253	11554596	133263	210105	2576289	92.5
chr08	9963694	8606835	41645	102451	1212763	92.2
chr09	19670963	15353494	180513	306436	3830520	92.6
chr10	18631979	14286623	159945	305230	3880181	92.7
Total	223817606			3338118	43177620	92.8

Supplementary Table S15. ***Percentage nucleotide identity between pairs of homeologous gene copies obtained from Sanger sequencing.***

**Gene name**	**Length of alignment (bp)**	**CDS**		**Non-CDS**	
		**Number of sites**	**Percentage Identity**	**Number of sites**	**Percentage Identity**
CYP724B1	2103	326	98.4	1777	93.8
CYP90B2	874	487	97.3	387	70.3
ERA1	1489	203	89.6	1286	85.3
GA20ox2	1448	459	96.5	989	71.6
GW2	1340	225	96.4	1115	81.8
KO2	1596	332	96.6	1264	86.5
Tua1	2687	1354	97.2	1333	75.4
Tua2	1884	1041	98.2	843	93.3
**Total/average**	13421	4427	96.3	8994	82.2

Supplementary Table S16. ***Representation of the transcriptome in the genome***. The percentage of bases of each transcriptome found in the genome and the number of full-length copies of the transcripts in the genome using blastn e-value 1e-10 are tabulated.

<table>
<tr><th rowspan="3">Genome</th><th rowspan="3">Assembly</th><th colspan="2">Percentage of base pairs found in tef genome</th><th colspan="5">Number of genes aligned to tef genome</th></tr>
<tr><th rowspan="2">Copy 1</th><th rowspan="2">Copy 2</th><th colspan="2">Copy 1</th><th colspan="2">Copy 2</th><th rowspan="2">Total genes aligned</th></tr>
<tr><th>100%</th><th>80-99%</th><th>100%</th><th>80-99%</th></tr>
<tr><td rowspan="2">Tef</td><td>454Isotigs</td><td>95.7</td><td>86.3</td><td>18834</td><td>17878</td><td>6727</td><td>22888</td><td>38461</td></tr>
<tr><td>Extended</td><td>91.7</td><td>75.5</td><td>35377</td><td>41787</td><td>8886</td><td>43426</td><td>88081</td></tr>
<tr><td>Sorghum</td><td></td><td>56.8</td><td>49.4</td><td>165</td><td>3764</td><td>73</td><td>2347</td><td>29448</td></tr>
<tr><td colspan="9">Copy 1 and copy 2 are defined as the tef sequences which aligns with the highest number of query sequences bases and the second highest, respectively</td></tr>
</table>

Supplementary Table S17. ***Summary statistics of SSR markers found in the tef genome obtained by MISA*** (http://pgrc.ipk-gatersleben.de/misa/). A selected SSR was used for PCR amplification and sequencing in different tef varieties and wild *Eragrostis* species.

Results of Microsatellite search	
Total sequences examined (number of scaffolds)	405558
Total bp examined	672766097
Total number of SSRs discovered	162124
Number of sequences containing SSRs	23431
Number of sequences containing more than 1 SSR	9024
Number of SSRs present in compound formation	12999
Distribution of different repeat type classes	
Size of the repeat unit	Number of SSRs
1	110513
2	27880
3	19116
4	2008
5	2179
6	428

Supplementary Table S18. ***List of 22,833 selected Simple Sequence Repeats (SSRs) identified from scaffolds based on the search for tandem repeats***. Only SSRs with 3 or more repeated units were chosen. For each SSR marker forward and reverse primers were designed 50-100 bp up-stream and down-stream from the repeat position. This Table is provided as a supplementary file [*Supplementary_Material_6_TableS18*] (word file).

Supplementary Table S19. ***Primers for the amplification of a novel SSR marker***. The marker was amplified and found to vary in different ecotypes of tef. Forward and reverse primers were designed 50-100 bp up-stream and down-stream from the repeat position, respectively.

**SSR name**	**Scaffold name**	**Tandem repeats in the tef genome**			**SSR primers designed (5'-3')**	
		**Type**	**Length (bp)**	**Sequence**	**Forward primer**	**Reverse primer**
SSR3.3	4255	(AAG)6	18	AAGAAGAAGAAGAAGAAG	GGGAAGAGGAGTGTACAGA	CCCTGGCAACTGCTTTAAGA
SSR9.4	2788	(CTCCT)5	25	CTCCTCTCCTCTCCTCTCCTCTCCT	CTCATCTCCCACCCTCACTC	GTAGCCCAGATCAAACGACC

Supplementary Table S20. ***Number of annotations found by various tools***.

Type	Number of Entries of this type		
	**Extended transcriptome**	**454Isotigs**	**Maker predictions**
Total Proteins in dataset	88081	38461	42052
Total Proteins after ESTScan	70861		NA
Entries in Annotation File	68614	33027	42052
Database Reference (DR) annotations	22952	6453	7286
DR GO	16322	15	6
DR InterPro	19522	0	0
DR PROSITE	12997	6453	7286
Feature Table (FT) annotations	31010	33027	42052
FT Transmembrane	17316	5677	8555
FT Domains	7093	4746	5659
FT CHAIN	4529	33027	42052
FT Signal annotation	4482	1253	5833
FT Repeats	3452	2268	3250
FT Active Site	1723	671	1050
Entries with a Description (DE)	68614	33027	42052
Entries with no match	33241	0	0
Entries labeled putative	23415	844	1404
Entries with EC number	4329	768	1245
Comment block (CC)	49866	19292	26054
Keyword (KW)	49878	17954	31560

Supplementary Table S21. ***Representation of abiotic stress related genes in the tef genome.*** Percentage identity of selected protein sequences from rice, sorghum and *Arabidopsis thaliana* proteins found in the tef genome using tblastn. Genes having a second copy with more than 60% of the query aligned have two entries for tef.

**Type of abiotic stress**	**Gene Name**	**Query protein size (a.a.)**	**Organism**	**Reference**	**Name of tef scaffold**	**% of query aligned in**		
						**Tef**	**Sorghum**	**Setaria**
Drought tolerance	ABA receptor_py15_like	219	Sorghum	[45]	133<br>4259	68.0<br>68.0	98.6	98.6
	AP37	240	Sorghum	[46]	8580	75.8	100	87.1
	B glucanase	498	Sorghum	[47]	3771<br>3735	95.7<br>93.8	98.0	97.6
	bZIP23	257	Sorghum	[48]	447<br>446	100<br>100	100	100
	bZIP46	322	Sorghum	[48]	2300<br>2301	93.2<br>84.8	100	100
	C4 methyl oxidase	291	Sorghum	[49]	2443<br>7989	94.5<br>91.0	91.1	74.2
	ERA1	451	Sorghum	[50]	2224<br>520	94.2<br>93.3	100	94.0
	LEA3	201	Sorghum	[42]	7398<br>6095	88.1<br>79.6	100	56.2
	P5CS	714	Sorghum	[51]	2922<br>6139	91.2<br>88.8	95.8	88.8
	SAL1	410	Sorghum	[52]	1676<br>5634	75.6<br>74.1	80.5	80.5
	SGR	293	Sorghum	[53]	2056	78.5	78.5	78.5
	SNAC1	312	Sorghum	[54]	3685<br>8561	100<br>77.8	100	100
	Soluble Acid Invertase	677	Sorghum	[55]	2162<br>1346	75.9<br>73.5	99.8	100
Drought- & salt-tolerance	Glutamate Synthase	2169	Sorghum	[56]	2557<br>3578	95.5<br>95.5	93.7	98.4
	DREB1A	238	Sorghum	[57]	3759<br>2724	99.6<br>71.8	100	99.6
	Hardy	255	Arabidopsis	[58]	3009	57.2	97.6	89.4
Drought- & cold-tolerance	ABF3	339	Sorghum	[57]	1256	71.7	91.1	90.2
Drought-, cold- & salt-tolerance	COIN	381	Sorghum	[59]	3522<br>14478	99.7<br>96.3	99.2	99.2
Drought-, salt- & heat-tolerance	ERD1	936	Sorghum	[60]	4959<br>2435	92.6<br>92.6	92.6	92.6
Drought- & submergence-tolerance	SUB1A	225	Sorghum	[48, 61]	5655	74.2	92.4	91.1
Heat-tolerance	HSF7	358	Rice	[62]	3078<br>15326	94.4<br>92.7	100	95.0
Submergence tolerance	SK1	344	Sorghum	[48]	1860<br>11546	59.3<br>59.3	100	59.6
	SK2	333	Sorghum	[48]	2923<br>2035	69.7<br>68.8	88.3	69.7
Heavy metal tolerance	LSI1	295	Rice	[63]	6057<br>12826	94.9<br>90.8	95.6	95.6
Al-tolerance	MATE	626	Sorghum	[64]	404<br>5709	78.9<br>67.2	85.5	73.5
Salt-tolerance	NHX1	435	Rice	[65]	4380<br>5054	97.0<br>85.5	100	94.3

Supplementary Table S22. ***Abiotic stress genes and their numbers in grass genomes***. Number of copies of genes known from the literature to be implicated in abiotic stress have been counted in the genome of tef (*Eragrostis tef)*, sorghum (*Sorghum bicolor)*, rice (*Oryza sativa), Brachypodium distrachyum* and foxtail millet (*Setaria italica)*. For each genome, the number of matches having a length greater than or equal to 70% of the length of the query sequence is shown. Sources for indicated genes in different species are *B. distachyon* [66], *S. italica* [28], *Z. mays* [16], *S. bicolor* [15], *O. sativa* [67], *H. vulgare* [68], *S. cereal* [68], *T. aestivum* [68], and for *E.tef* (the current work). Abbreviations: E.t. = *Eragrostis tef;* S.b. = *Sorghum bicolor;* O.s. = *Oryza sativa;* B.d. = *Brachypodium distrachyum;* S.i. = *Setaria itálica. ([1] = flooding, [2] = drought, [3] = control)*

Trait/tolerance	Gene Name	Reference	Protein query search (hits >70%)												
			Genome					Transcriptome							
			E.t.	*S.b.*	*O.s.*	*B.d.*	*S.i.*	*E.t.*	*S.b.*	*O.s.*	*B.d.*	*S.i.*	*E.t.*	*E.t.*	*E.t.*
Drought	ABA_receptor_py15_like	[45]	0	2	2	2	2	5	2	2	3	0	0	0	0
	AP37	[46]	2	3	3	2	3	6	3	3	3	3	0	0	0
	B_glucanase	[47]	46	10	11	5	9	83	61	74	55	90	6	0	7
	bZIP23	[48]	5	2	2	2	3	5	2	3	2	3	3	3	3
	bZIP46	[48]	4	5	4	3	5	13	6	5	8	6	0	0	0
	C4_methyl_oxidase	[49]	8	5	3	4	3	10	4	6	7	7	3	3	2
	ERA1	[50]	2	1	1	1	1	4	2	2	2	2	0	0	0
	LEA3	[42]	2	1	0	0	0	3	3	0	0	0	0	0	0
	P5CS	[51]	7	3	2	2	2	26	5	4	7	6	0	0	0
	SAL1	[69]	6	2	2	2	2	16	5	5	3	4	0	0	0
	SGR	[53]	2	2	2	2	2	7	2	2	2	2	2	0	0
	SNAC1	[54]	3	4	4	2	3	19	5	3	6	3	0	0	0
	Soluble_acid_invertase	[55]	4	5	4	4	5	18	14	9	12	16	0	0	0
Drought/salt	Glutamate_synthase	[56]	5	3	3	2	3	3	3	5	4	3	0	0	0
	DREB1A	[57]	3	3	4	4	4	10	7	4	12	6	0	0	0
	HARDY	[58]	0	3	3	3	4	4	3	3	2	6	0	0	0
Drought/cold	ABF3	[57]	2	4	3	3	3	7	5	4	6	5	0	0	0
Drought/cold/salt	COIN	[59]	5	3	2	1	2	5	3	4	4	4	0	0	0
Drought/salt/heat	ERD1	[60]	10	5	4	5	4	35	12	8	15	15	3	0	2
Drought/submergence	SUB1A	[48, 61]	2	2	2	2	2	2	2	2	2	2	0	0	0
Heat	HSF7	[62]	10	4	6	4	3	20	7	6	8	7	5	5	4
Sub-mergence	SK1	[48]	0	2	0	0	0	3	2	0	0	0	0	0	0
	SK2	[48]	0	2	0	0	0	4	3	3	2	3	0	0	0
Heavy metal	LSI1	[63]	17	6	9	5	7	35	28	31	24	33	4	2	5
Al	MATE	[64]	2	2	2	0	2	13	11	6	10	8	0	0	0
Salt	NHX1	[65]	8	4	2	4	4	24	8	7	6	11	3	4	2

Supplementary Table S23. ***Presence of wheat, barley and rye gluten epitopes and their amounts in grass genomes***. Epitopes of lengths 20, 16, 13, 12 and 11 from wheat, barley and rye from [22] have been searched in several grass genomes. The epitopes were found only in wheat, barley and rye. No epitope was found in tef, sorghum, setaria, bracypodium, rice or mays. Sources for *B. distachyon*, *S. italica*, *Z. mays*, and *S. bicolor* were Phytozome; for *O. sativa* was IRGSP*;* for *H. vulgare* was MIPS; *S. cereal* and *T. aestivum* were NCBI; and for *E.tef* was current work. Columns with gray shading show three species with gluten reaction while the other columns indicate six species with no gluten reaction. Sources for indicated genes in different species are *B. distachyon* [66], *S. italica* [28], *Z. mays* [16], *S. bicolor* [15], *O. sativa* [67], *H. vulgare* [68], *S. cereal* [68], *T. aestivum* [68], and for *E.tef* (the current work). ([1] = taxid:4550, [2] = taxid:4565)

Query gluten epitope			Number of counts in								
Length	Source	Number analyzed	**Brachypodium (*B. distachyon*)**	**Foxtailmillet (*S. italica)***	**Maize (*Z. mays)***	**Rice (*O. sativa)***	**Sorghum (*S. bicolor*)**	**Tef (*E. tef)***	**Barley (*H. vulgare)***	**Rye (*S. cereal)*[1]**	**Wheat (*T. aestivum*)[2]**
20 aa	Barley	30	0	0	0	0	0	0	13	0	3
	Rye	29	0	0	0	0	0	0	0	157	373
	Wheat	37	0	0	0	0	0	0	2	18	465
core 16 aa	Barley	8	0	0	0	0	0	0	6	0	0
	Rye	2	0	0	0	0	0	0	0	16	27
	Wheat	8	0	0	0	0	0	0	0	111	216
core 13 aa	Barley	2	0	0	0	0	0	0	1	0	0
	Rye	0	0	0	0	0	0	0	0	0	0
	Wheat	0	0	0	0	0	0	0	0	0	0
core 12 aa	Barley	17	0	0	0	0	0	0	13	20	166
	Rye	25	0	0	0	0	0	0	5	222	889
	Wheat	25	0	0	0	0	0	0	8	111	893
Core 11 aa	Barley	3	0	0	0	0	0	0	3	0	0
	Rye	2	0	0	0	0	0	0	0	2	0
	Wheat	4	0	0	0	0	0	0	0	7	49

Supplementary Table S24. Summary of prolamin genes found in the tef genome and transcriptomes.

[1] = Obtained by blasting sequences to Xu & Messing [70]; [2] = found by search with 31 aa long-expressed sequence tef6 of Tatham, corresponds to isotig15824, trinity comp65133_c0_seq1; [3] = found blasting transcript CL17177Contig1 in genome; [4] = found blasting the 30 amino acids of the tef2 sequence of Tatham in the genome; fs = frame shift, stop = stop codon.

Tef scaffold	Tef pseudo chromosome	Location on scaffold	Status	E-value	Tef transcripts expressed
**Similar to alpha-globulin**[1]					
4989	9	81564-82218	fs	7e-22	
4989	9	83500-84499	Ok	3e-28	
4451	9	231665-231108	fs	3e-22	
4451	9	232725-233332	fs	3e-22	
**Alpha-type**[2]					
10996		99860-99519	ok		Tef6, isotig15824
1514	1	1116-1033	stop		
**Delta-type**[1]					
958	10	90259-89774	fs	7e-08	
7847	3	34606-34000		2e-06	comp48369_c0_seq1, CL17177Contig1
7847	3	37903-37616	stop	7e-09	
5675		48236-48712	fs	1e-09	
5655	3	56551-56052	fs	3e-06	
304	3	99860-99519	stop	6e-08	
304	3	116715-116415	ok	1e-06	
2167		10545-10916	ok	2e-12	
2167		13515-13886	ok	2e-12	
2167		14475-14846	ok	2e-12	
**Delta-type**[3]					
5655	3	38971-38630	stop		
1756	3	30017-29652	ok		
3719		4671-4442	fs		
966		16634-16320	ok		
11		217494-217175	fs		
**Others**[4]					
3597	9	35548-35637		9e-15	Tef2
3597	9	38915-39004		9e-15	Tef2

3597	9	43692-43781		1e-10	
7998		18885-18974		1e-14	Tef2
C6520620		100-11		3e-13	
14811		331-420		7e-11	
5216		25716-25805		4e-10	
5216		1127-1038	stop	1e-08	
13371		333-422	stop	3e-09	
1297	2a	66103-66017		5e-09	
1101		127324-127238		1e-08	

References

1. Zeid M, Belay G, Mulkey S, Poland J, Sorrells ME: **QTL mapping for yield and lodging resistance in an enhanced SSR-based map for tef**. *TAG Theoretical and applied genetics Theoretische und angewandte Genetik* 2010, **122**(1):77-93.
2. Gonnet GH, Hallett MT, Korostensky C, Bernardin L: **Darwin v. 2.0: an interpreted computer language for the biosciences.** *Bioinformatics* 2000, **16**(2):101-103.
3. Murray MG, Thompson WF: **Rapid Isolation of High Molecular-Weight Plant DNA**. *Nucleic Acids Res* 1980, **8**(19):4321-4325.
4. Schuelke M: **An economic method for the fluorescent labeling of PCR fragments**. *Nat Biotechnol* 2000, **18**(2):233-234.
5. Zerr T, Henikoff S: **Automated band mapping in electrophoretic gel images using background information**. *Nucleic Acids Res* 2005, **33**(9):2806-2812.
6. Lyons E, Freeling M: **How to usefully compare homologous plant genes and chromosomes as DNA sequences**. *The Plant Journal* 2008, **53**(4):661-673.
7. Lyons E, Pedersen B, Kane J, Alam M, Ming R, Tang HB, Wang XY, Bowers J, Paterson A, Lisch D *et al*: **Finding and Comparing Syntenic Regions among Arabidopsis and the Outgroups Papaya, Poplar, and Grape: CoGe with Rosids**. *Plant Physiol* 2008, **148**(4):1772-1781.
8. Altschul SF, Gish W, Miller W, Myers EW, Lipman DJ: **Basic Local Alignment Search Tool**. *J Mol Biol* 1990, **215**(3):403-410.
9. Haas BJ, Delcher AL, Wortman JR, Salzberg S: **DAGchainer: a tool for mining segmental genome duplications and synteny.** *Bioinformatics* 2004, **20**(18):3643-3646.
10. Tang HB, Lyons E, Pedersen B, Schnable JC, Paterson AH, Freeling M: **Screening synteny blocks in pairwise genome comparisons through integer programming**. *Bmc Bioinformatics* 2011, **12**.
11. Schwartz S, Kent WJ, Smit A, Zhang Z: **Human–mouse alignments with BLASTZ**. *Genome* 2003, **13**(1):103-107.
12. Frith MC, Hamada M, Horton P: **Parameters for accurate genome alignment**. *Bmc Bioinformatics* 2010, **11**.
13. Haas BJ, Delcher AL, Wortman JR, Salzberg SL: **DAGchainer: a tool for mining segmental genome duplications and synteny**. *Bioinformatics* 2004, **20**(18):3643-3646.
14. Krzywinski M, Schein J, Birol I, Connors J, Gascoyne R, Horsman D, Jones SJ, Marra MA: **Circos: an information aesthetic for comparative genomics**. *Genome Res* 2009, **19**(9):1639-1645.
15. Paterson AH, Bowers JE, Bruggmann R, Dubchak I, Grimwood J, Gundlach H, Haberer G, Hellsten U, Mitros T, Poliakov A *et al*: **The Sorghum bicolor genome and the diversification of grasses**. *Nature* 2009, **457**(7229):551-556.
16. Schnable PS, Ware D, Fulton RS, Stein JC, Wei FS, Pasternak S, Liang CZ, Zhang JW, Fulton L, Graves TA *et al*: **The B73 Maize Genome: Complexity, Diversity, and Dynamics**. *Science* 2009, **326**(5956):1112-1115.
17. Goff SA: **A Draft Sequence of the Rice Genome (Oryza sativa L. ssp. japonica)**. *Science (New York, NY)* 2002, **296**(5565):92-100.
18. Zhang G, Liu X, Quan Z, Cheng S, Xu X, Pan S, Xie M, Zeng P, Yue Z, Wang W *et al*: **Genome sequence of foxtail millet (Setaria italica) provides insights into grass evolution and biofuel potential**. *Nat Biotechnol* 2012, **30**(6):549-554.
19. Yang Z: **PAML 4: phylogenetic analysis by maximum likelihood**. *Molecular biology and evolution* 2007, **24**(8):1586-1591.

20. Gaut BS, Morton BR, McCaig BC, Clegg MT: **Substitution rate comparisons between grasses and palms: Synonymous rate differences at the nuclear gene Adh parallel rate differences at the plastid gene rbcL**. *Proc Natl Acad Sci U S A* 1996, **93**(19):10274-10279.
21. Pedruzzi I, Rivoire C, Auchincloss AH, Coudert E, Keller G, de Castro E, Baratin D, Cuche BA, Bougueleret L, Poux S *et al*: **HAMAP in 2013, new developments in the protein family classification and annotation system.** *Nucleic Acids Res* 2013:584-589.
22. Tye-Din JA, Stewart JA, Dromey JA, Beissbarth T, van Heel DA, Tatham A, Henderson K, Mannering SI, Gianfrani C, Jewell DP *et al*: **Comprehensive, Quantitative Mapping of T Cell Epitopes in Gluten in Celiac Disease**. *Science Translational Medicine* 2010, **2**(41):41ra51-41ra51.
23. Delcher AL, Salzberg SL, Phillippy AM: **Using MUMmer to identify similar regions in large sequence sets.** *Current protocols in bioinformatics / editoral board, Andreas D Baxevanis [et al]* 2003, **Chapter 10**:Unit-Uni3.
24. Marcais G, Kingsford C: **A fast, lock-free approach for efficient parallel counting of occurrences of k-mers**. *Bioinformatics* 2011, **27**(6):764-770.
25. Argout X, Salse J, Aury J-M, Guiltinan MJ, Droc G, Gouzy J, Allegre M, Chaparro C, Legavre T, Maximova SN *et al*: **The genome of Theobroma cacao**. *Nat Genet* 2010, **43**(2):101-108.
26. Huang S, Li R, Zhang Z, Li L, Gu X, Fan W, Lucas WJ, Wang X, Xie B, Ni P *et al*: **The genome of the cucumber, Cucumis sativus L.** *Nat Genet* 2009, **41**(12):1275-1281.
27. Al-Dous EK, George B, Al-Mahmoud ME, Al-Jaber MY, Wang H, Salameh YM, Al-Azwani EK, Chaluvadi S, Pontaroli AC, DeBarry J *et al*: **De novo genome sequencing and comparative genomics of date palm (Phoenix dactylifera)**. *Nat Biotechnol* 2011, **29**(6):521-527.
28. Bennetzen JL, Schmutz J, Wang H, Percifield R, Hawkins J, Pontaroli AC, Estep M, Feng L, Vaughn JN, Grimwood J *et al*: **Reference genome sequence of the model plant Setaria**. *Nat Biotechnol* 2012, **30**(6):555-561.
29. Guo S, Zhang J, Sun H, Salse J, Lucas WJ, Zhang H, Zheng Y, Mao L, Ren Y, Wang Z *et al*: **The draft genome of watermelon (Citrullus lanatus) and resequencing of 20 diverse accessions**. *Nat Genet* 2012, **45**(1):51-58.
30. Yamamuro C, Ihara Y, Wu X, Noguchi T, Fujioka S, Takatsuto S, Ashikari M, Kitano H, Matsuoka M: **Loss of function of a rice brassinosteroid insensitive1 homolog prevents internode elongation and bending of the lamina joint**. *The Plant cell* 2000, **12**(9):1591-1606.
31. Sakamoto T, Morinaka Y, Ohnishi T, Sunohara H, Fujioka S, Ueguchi-Tanaka M, Mizutani M, Sakata K, Takatsuto S, Yoshida S *et al*: **Erect leaves caused by brassinosteroid deficiency increase biomass production and grain yield in rice**. *Nat Biotechnol* 2006, **24**(1):105-109.
32. Hong Z, Ueguchi-Tanaka M, Umemura K, Uozu S, Fujioka S, Takatsuto S, Yoshida S, Ashikari M, Kitano H, Matsuoka M: **A rice brassinosteroid-deficient mutant, ebisu dwarf (d2), is caused by a loss of function of a new member of cytochrome P450**. *The Plant cell* 2003, **15**(12):2900-2910.
33. Rieu I, Ruiz-Rivero O, Fernandez-Garcia N, Griffiths J, Powers SJ, Gong F, Linhartova T, Eriksson S, Nilsson O, Thomas SG *et al*: **The gibberellin biosynthetic genes AtGA20ox1 and AtGA20ox2 act, partially redundantly, to promote growth and development throughout the Arabidopsis life cycle**. *The Plant journal : for cell and molecular biology* 2008, **53**(3):488-504.

34. Itoh H, Tatsumi T, Sakamoto T, Otomo K, Toyomasu T, Kitano H, Ashikari M, Ichihara S, Matsuoka M: **A rice semi-dwarf gene, Tan-Ginbozu (D35), encodes the gibberellin biosynthesis enzyme, ent-kaurene oxidase**. *Plant Mol Biol* 2004, **54**(4):533-547.
35. Yamamoto E, Zeng LH, Baird WV: **alpha-tubulin missense mutations correlate with antimicrotubule drug resistance in Eleusine indica**. *Plant Cell* 1998, **10**(2):297-308.
36. Zou JH, Zhang SY, Zhang WP, Li G, Chen ZX, Zhai WX, Zhao XF, Pan XB, Xie Q, Zhu LH: **The rice HIGH-TILLERING DWARF1 encoding an ortholog of Arabidopsis MAX3 is required for negative regulation of the outgrowth of axillary buds**. *Plant J* 2006, **48**(5):687-696.
37. Peng JR, Richards DE, Hartley NM, Murphy GP, Devos KM, Flintham JE, Beales J, Fish LJ, Worland AJ, Pelica F *et al*: **'Green revolution' genes encode mutant gibberellin response modulators**. *Nature* 1999, **400**(6741):256-261.
38. Ashikari M, Sakakibara H, Lin SY, Yamamoto T, Takashi T, Nishimura A, Angeles ER, Qian Q, Kitano H, Matsuoka M: **Cytokinin oxidase regulates rice grain production**. *Science* 2005, **309**(5735):741-745.
39. Song XJ, Huang W, Shi M, Zhu MZ, Lin HX: **A QTL for rice grain width and weight encodes a previously unknown RING-type E3 ubiquitin ligase**. *Nat Genet* 2007, **39**(5):623-630.
40. Shomura A, Izawa T, Ebana K, Ebitani T, Kanegae H, Konishi S, Yano M: **Deletion in a gene associated with grain size increased yields during rice domestication**. *Nat Genet* 2008, **40**(8):1023-1028.
41. Pei ZM, Ghassemian M, Kwak CM, McCourt P, Schroeder JI: **Role of farnesyltransferase in ABA regulation of guard cell anion channels and plant water loss**. *Science* 1998, **282**(5387):287-290.
42. Xiao B, Huang Y, Tang N, Xiong L: **Over-expression of a LEA gene in rice improves drought resistance under the field conditions**. *Theoretical and Applied Genetics* 2007, **115**(1):35-46.
43. SATHASIVAN K, HAUGHN GW, MURAI N: **Molecular-Basis of Imidazolinone Herbicide Resistance in Arabidopsis-Thaliana Var Columbia**. *Plant Physiol* 1991, **97**(3):1044-1050.
44. Smith SM, Yuan Y, Doust AN, Bennetzen JL: **Haplotype Analysis and Linkage Disequilibrium at Five Loci in Eragrostis tef**. *G3 (Bethesda)* 2012, **2**(3):407-419.
45. Santiago J, Rodrigues A, Saez A, Rubio S, Antoni R, Dupeux F, Park S-Y, M rquez JA, Cutler SR, Rodriguez PL: **Modulation of drought resistance by the abscisic acid receptor PYL5 through inhibition of cladeA PP2Cs**. *The Plant Journal* 2009, **60**(4):575-588.
46. Oh SJ, Kim YS, Kwon CW, Park HK, Jeong JS, Kim JK: **Overexpression of the Transcription Factor AP37 in Rice Improves Grain Yield under Drought Conditions**. *Plant Physiol* 2009, **150**(3):1368-1379.
47. Akiyama T, Pillai MA: **Molecular cloning, characterization and in vitro expression of a novel endo-1,3-beta-glucanase up-regulated by ABA and drought stress in rice (Oryza sativa L.)**. *Plant Sci* 2001, **161**(6):1089-1098.
48. Fukao T, Xiong LZ: **Genetic mechanisms conferring adaptation to submergence and drought in rice: simple or complex?** *Current Opinion in Plant Biology* 2013, **16**(2):196-204.
49. Guo PG, Baum M, Grando S, Ceccarelli S, Bai GH, Li RH, von Korff M, Varshney RK, Graner A, Valkoun J: **Differentially expressed genes between drought-tolerant and drought-sensitive barley genotypes in response to drought stress during the reproductive stage**. *J Exp Bot* 2009, **60**(12):3531-3544.

50. Wang Y, Ying JF, Kuzma M, Chalifoux M, Sample A, McArthur C, Uchacz T, Sarvas C, Wan JX, Dennis DT *et al*: **Molecular tailoring of farnesylation for plant drought tolerance and yield protection**. *Plant J* 2005, **43**(3):413-424.
51. Choudhary NL, Sairam RK, Tyagi A: **Expression of Delta(1)-pyrroline-5-carboxylate synthetase gene during drought in rice (Oryza sativa L.)**. *Indian Journal of Biochemistry & Biophysics* 2005, **42**(6):366-370.
52. Wilson PB, Estavillo GM, Field KJ, Pornsiriwong W, Carroll AJ, Howell KA, Woo NS, Lake JA, Smith SM, Harvey Millar A *et al*: **The nucleotidase/phosphatase SAL1 is a negative regulator of drought tolerance in Arabidopsis**. *The Plant journal : for cell and molecular biology* 2009, **58**(2):299-317.
53. Jiang H, Li M, Liang N, Yan H, Wei Y, Xu X, Liu J, Xu Z, Chen F, Wu G: **Molecular cloning and function analysis of the stay green gene in rice**. *Plant J* 2007, **52**(2):197-209.
54. Hu H, Dai M, Yao J, Xiao B, Li X, Zhang Q, Xiong L: **Overexpressing a NAM, ATAF, and CUC (NAC) transcription factor enhances drought resistance and salt tolerance in rice**. *Proc Natl Acad Sci U S A* 2006, **103**(35):12987-12992.
55. Andersen MN, Asch F, Wu Y, Jensen CR, Naested H, Mogensen VO, Koch KE: **Soluble invertase expression is an early target of drought stress during the critical, abortion-sensitive phase of young ovary development in maize**. *Plant Physiol* 2002, **130**(2):591-604.
56. Kalamaki MS, Alexandrou D, Lazari D, Merkouropoulos G, Fotopoulos V, Pateraki I, Aggelis A, Carrillo-Lopez A, Rubio-Cabetas MJ, Kanellis AK: **Over-expression of a tomato N-acetyl-L-glutamate synthase gene (SlNAGS1) in Arabidopsis thaliana results in high ornithine levels and increased tolerance in salt and drought stresses**. *Journal of Experimental Botany* 2009, **60**(6):1859-1871.
57. Oh SJ, Song SI, Kim YS, Jang HJ, Kim SY, Kim M, Kim YK, Nahm BH, Kim JK: **Arabidopsis CBF3/DREB1A and ABF3 in transgenic rice increased tolerance to abiotic stress without stunting growth**. *Plant Physiol* 2005, **138**(1):341-351.
58. Karaba A, Dixit S, Greco R, Aharoni A, Trijatmiko KR, Marsch-Martinez N, Krishnan A, Nataraja KN, Udayakumar M, Pereira A: **Improvement of water use efficiency in rice by expression of HARDY, an Arabidopsis drought and salt tolerance gene**. *Proc Natl Acad Sci U S A* 2007, **104**(39):15270-15275.
59. Liu K, Wang L, Xu Y, Chen N, Ma Q, Li F, Chong K: **Overexpression of OsCOIN, a putative cold inducible zinc finger protein, increased tolerance to chilling, salt and drought, and enhanced proline level in rice**. *Planta* 2007, **226**(4):1007-1016.
60. Nakashima K, Kiyosue T, YamaguchiShinozaki K, Shinozaki K: **A nuclear gene, erd1 encoding a chloroplast-targeted Clp protease regulatory subunit homolog is not only induced by water stress but also developmentally up-regulated during senescence in Arabidopsis thaliana**. *Plant J* 1997, **12**(4):851-861.
61. Xu K, Xu X, Fukao T, Canlas P, Maghirang-Rodriguez R, Heuer S, Ismail AM, Bailey-Serres J, Ronald PC, Mackill DJ: **Sub1A is an ethylene-response-factor-like gene that confers submergence tolerance to rice**. *Nature* 2006, **442**(7103):705-708.
62. Liu J-G, Qin Q-l, Zhang Z, Peng R-H, Xiong A-s, Chen J-M, Yao Q-H: **OsHSF7 gene in rice, Oryza sativa L., encodes a transcription factor that functions as a high temperature receptive and responsive factor**. *Bmb Reports* 2009, **42**(1):16-21.
63. Ma JF, Tamai K, Yamaji N, Mitani N, Konishi S, Katsuhara M, Ishiguro M, Murata Y, Yano M: **A silicon transporter in rice**. *Nature* 2006, **440**(7084):688-691.
64. Magalhaes JV, Liu J, Guimaraes CT, Lana UGP, Alves VMC, Wang YH, Schaffert RE, Hoekenga OA, Pineros MA, Shaff JE *et al*: **A gene in the multidrug and toxic compound extrusion (MATE) family confers aluminum tolerance in sorghum**. *Nat Genet* 2007, **39**(9):1156-1161.

65. Ohta M, Hayashi Y, Nakashima A, Hamada A, Tanaka A, Nakamura T, Hayakawa T: **Introduction of a Na+/H+ antiporter gene from Atriplex gmelini confers salt tolerance to rice**. *Febs Lett* 2002, **532**(3):279-282.
66. Vogel JP, Garvin DF, Mockler TC, Schmutz J, Rokhsar D, Bevan MW, Barry K, Lucas S, Harmon-Smith M, Lail K *et al*: **Genome sequencing and analysis of the model grass Brachypodium distachyon**. *Nature* 2010, **463**(7282):763-768.
67. Ouyang S, Zhu W, Hamilton J, Lin H, Campbell M, Childs K, Thibaud-Nissen F, Malek RL, Lee Y, Zheng L *et al*: **The TIGR Rice Genome Annotation Resource: Improvements and new features**. *Nucleic Acids Res* 2007, **35**:D883-D887.
68. Sayers EW, Barrett T, Benson DA, Bryant SH, Canese K, Chetvernin V, Church DM, DiCuccio M, Edgar R, Federhen S *et al*: **Database resources of the National Center for Biotechnology Information**. *Nucleic Acids Res* 2009, **37**(Database issue):D5-15.
69. Wilson PB, Estavillo GM, Field KJ, Pornsiriwong W, Carroll AJ, Howell KA, Woo NS, Lake JA, Smith SM, Millar AH *et al*: **The nucleotidase/phosphatase SAL1 is a negative regulator of drought tolerance in Arabidopsis**. *Plant J* 2009, **58**(2):299-317.
70. Xu JH, Messing J: **Amplification of prolamin storage protein genes in different subfamilies of the Poaceae - Springer**. *Theoretical and Applied Genetics* 2009, **119**(8):1397-1412.
