# Supplementary material for: Genome and transcriptome sequencing identifies breeding targets in the orphan crop tef (Eragrostis tef)
Source: BMC Genomics. 2014 Jul 9;15(1):581. doi: 10.1186/1471-2164-15-581 (PMC4119204; doi:10.1186/1471-2164-15-581)
Supplement: Supplementary file 4 — Additional file 4: Figure S4: Alignment of A and B genomes. (DOCX 2 MB) [file 12864_2014_6309_MOESM4_ESM.docx]

Chromosome 1


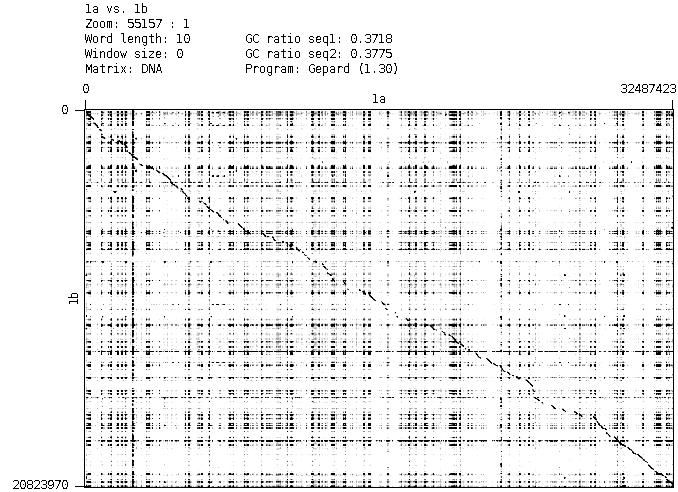


Chromosome 2


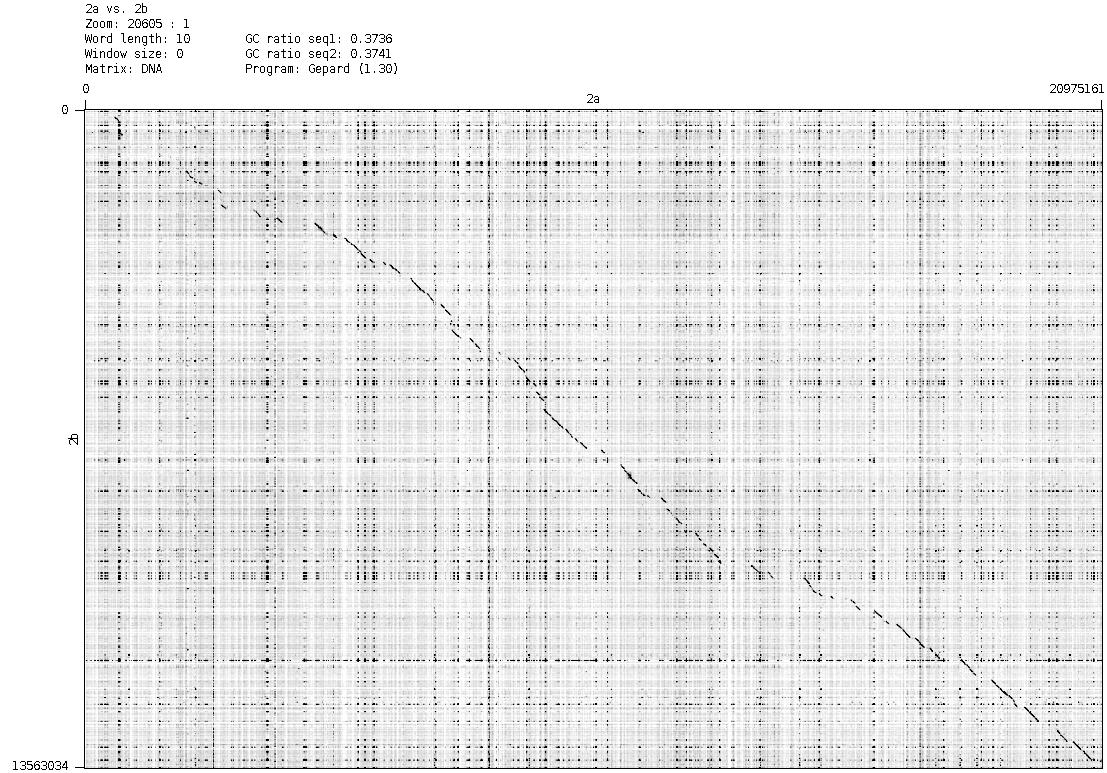


Chromosome 3


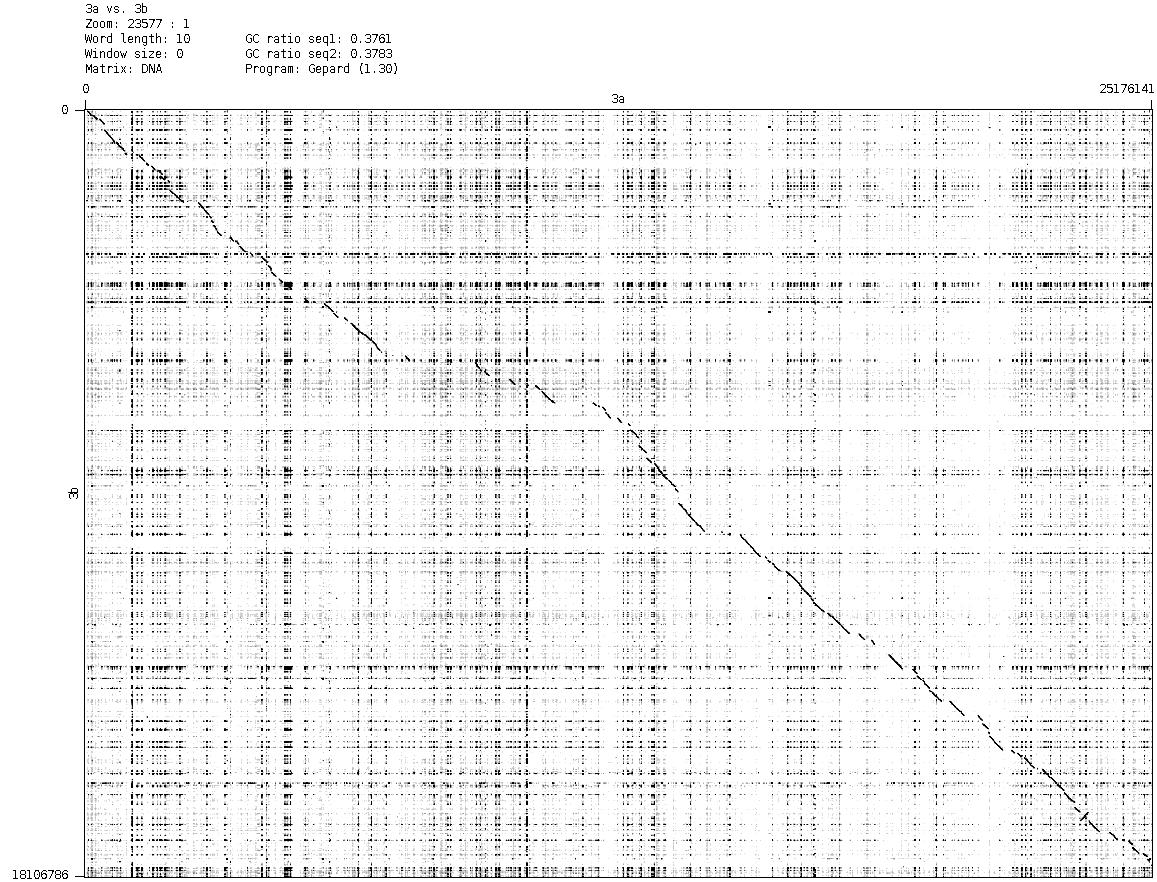


Chromosome 4


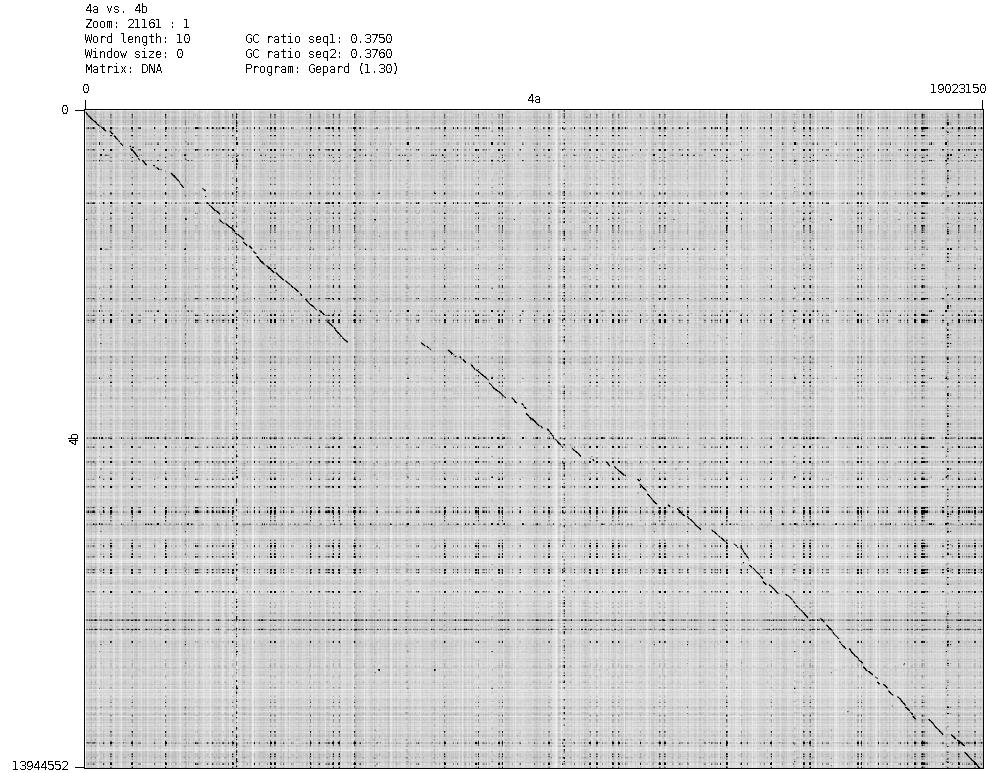


Chromosome 5
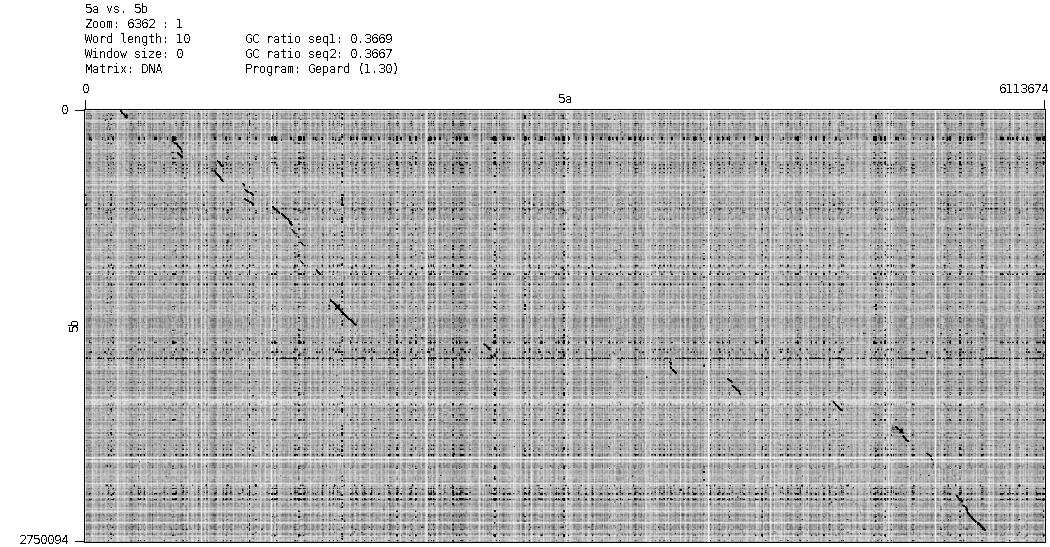


Chromosome 6


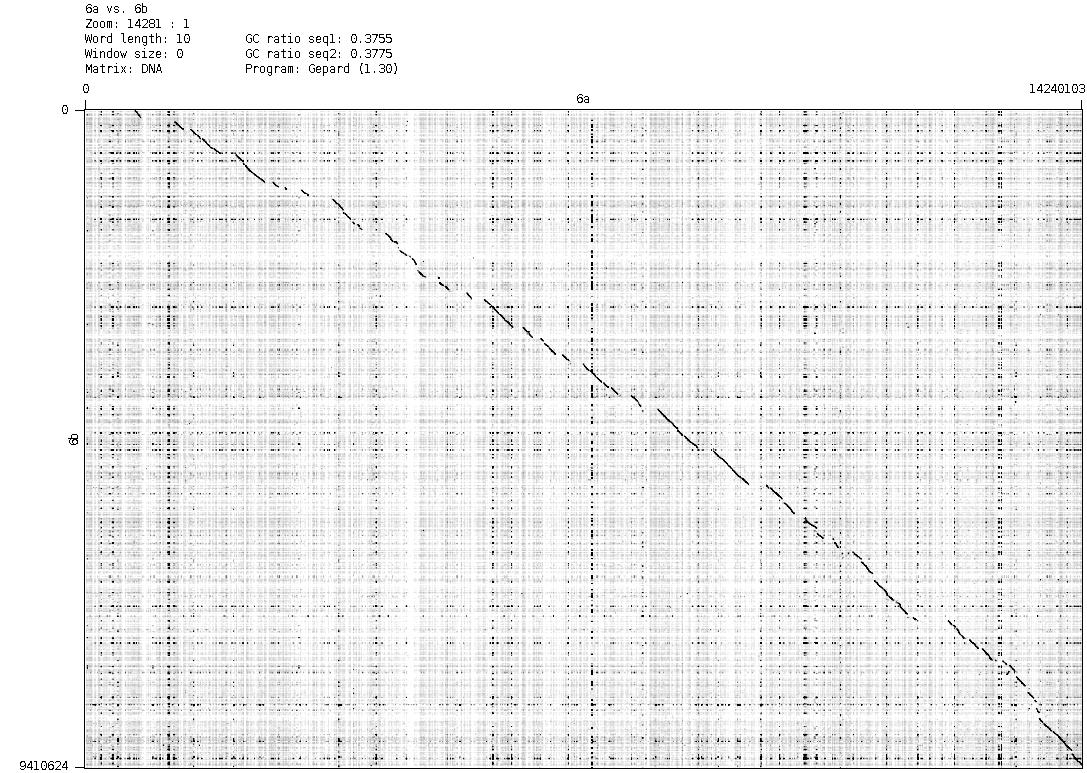


Chromosome 7


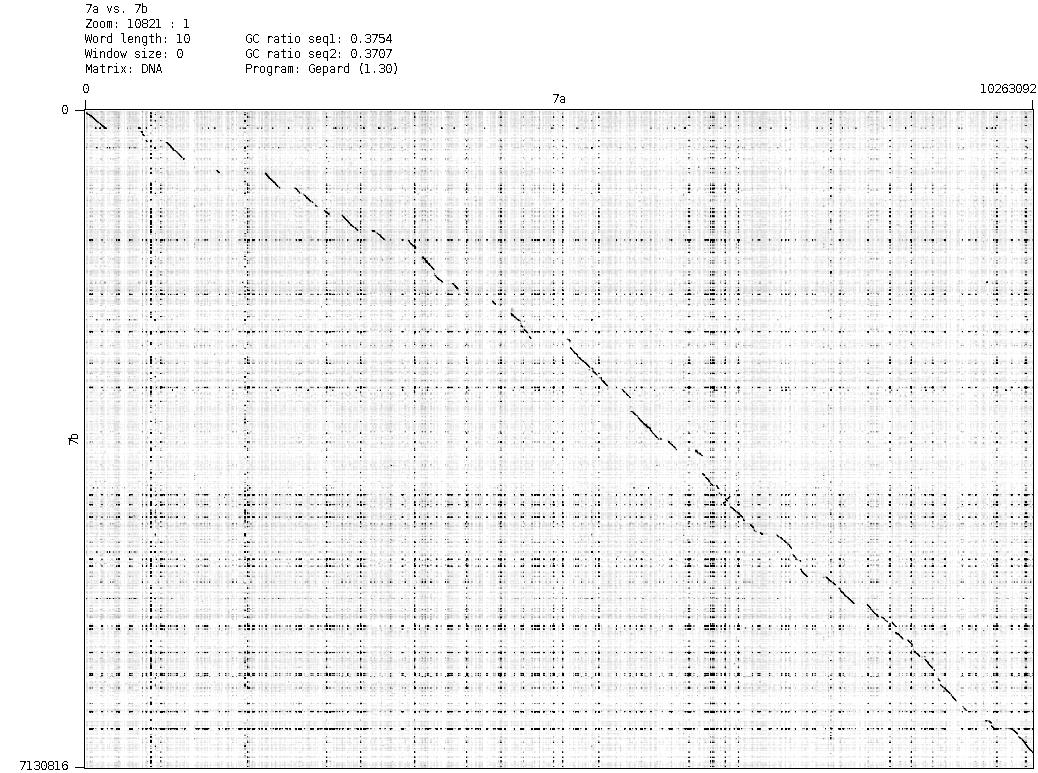


Chromosome 8


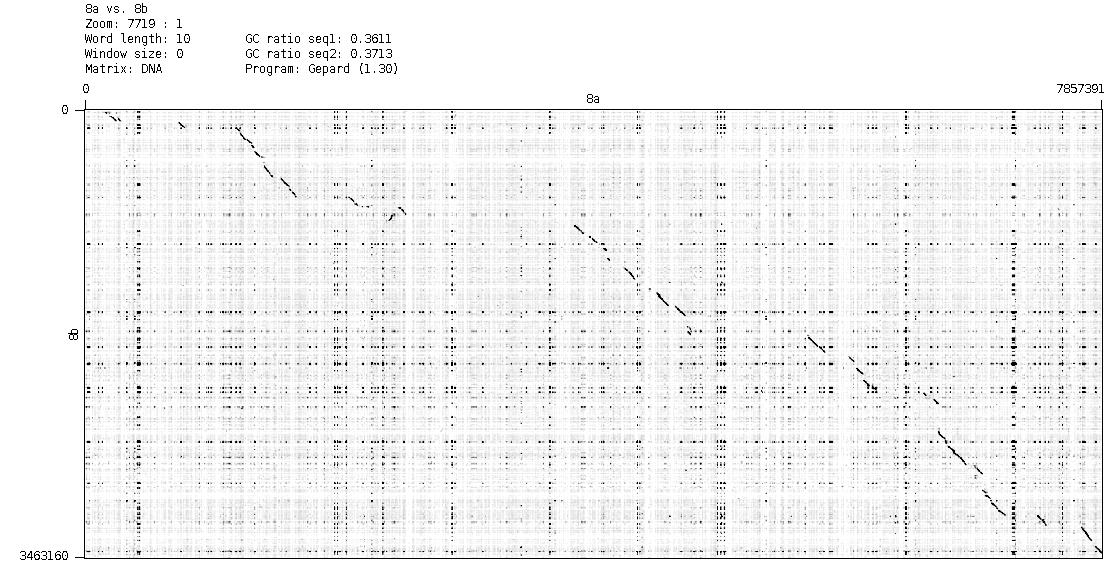


Chromosome 9


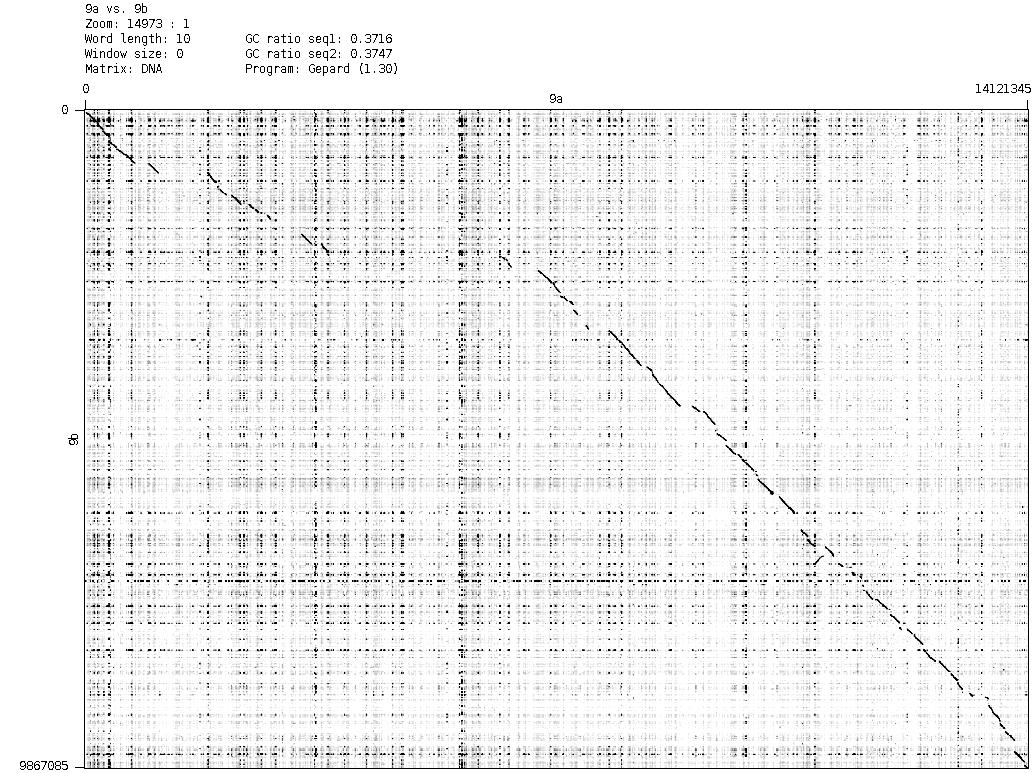


Chromosome 10


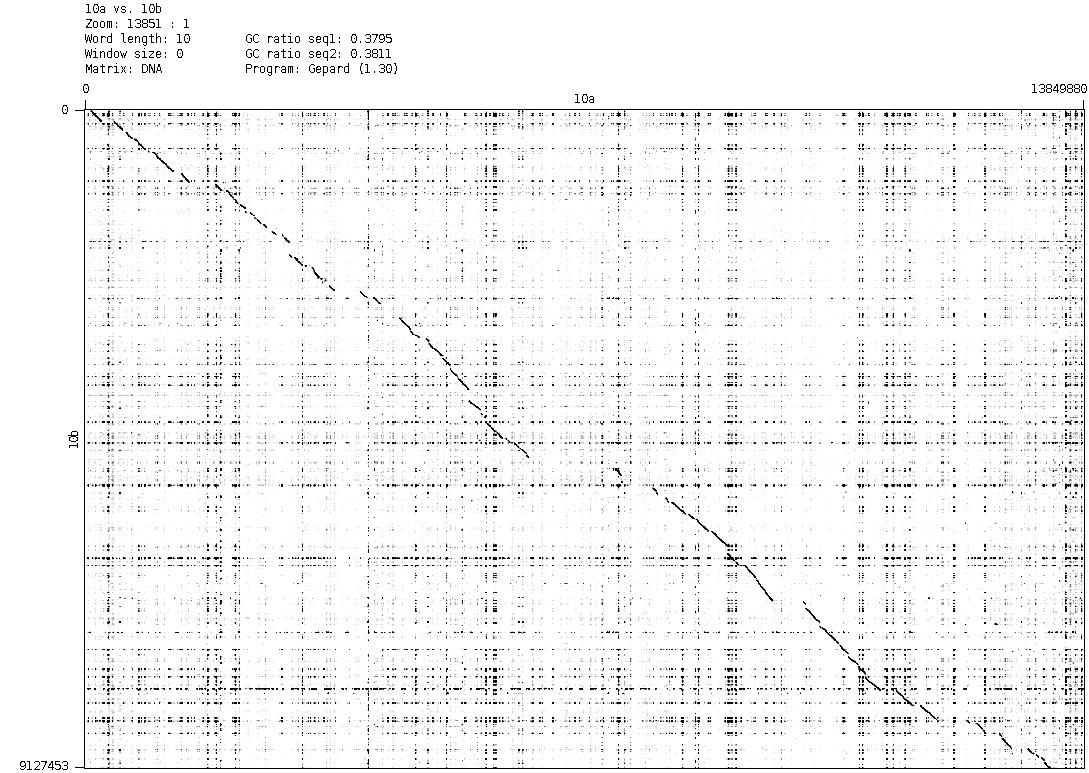


**Supplementary Figure S2. *Alignment of A and B genomes***. Scaffolds were ordered by mapping to sorghum to individual sorghum chromosomes and were then sorted into an A and a B genome by to avoid overlap. A dotplot shows the correspondence between the A and B genomes.
